# Inconsistency in Abnormal Functional Connectivity Across Datasets of ADHD-200 in Children With Attention Deficit Hyperactivity Disorder

**DOI:** 10.3389/fpsyt.2019.00692

**Published:** 2019-09-27

**Authors:** Zhi-Wei Zhou, Yan-Tong Fang, Xia-Qing Lan, Li Sun, Qing-Jiu Cao, Yu-Feng Wang, Hong Luo, Yu-Feng Zang, Hang Zhang

**Affiliations:** ^1^Institute of Psychological Sciences, College of Education, Hangzhou Normal University, Hangzhou, China; ^2^Center for Cognition and Brain Disorders and the Affiliated Hospital, Hangzhou Normal University, Hangzhou, China; ^3^Zhejiang Key Laboratory for Research in Assessment of Cognitive Impairments, Hangzhou, China; ^4^Institute of Mental Health, The Sixth Hospital, Peking University, Beijing, China

**Keywords:** attention deficit hyperactivity disorder, resting-state fMRI, multi-site dataset, ADHD-200, functional connectivity

## Abstract

Many studies have shown abnormal functional connectivity in children with attention deficit hyperactivity disorder (ADHD) by using resting-state functional magnetic resonance imaging (rs-fMRI). However, few studies illustrated that to what extent these findings were consistent across different datasets. The present study aimed to assess the consistency of abnormal functional connectivity in children with ADHD across the four datasets from a public-assess rs-fMRI ADHD cohort, namely, ADHD-200. We employed the identical analysis process of previous studies and examined a few factors, including connectivity with the seed regions of the bilateral dorsal anterior cingulate cortex, bilateral inferior frontal gyrus, and bilateral middle frontal gyrus; connectivity between default mode network and executive control network; stringent and lenient statistical thresholds; and the ADHD subtypes. Our results revealed a high inconsistency of abnormal seed-based connectivity in children with ADHD across all datasets, even across three datasets from the same research site. This inconsistency could also be observed with a lenient statistical threshold. Besides, each dataset did not show abnormal connectivity between default mode network and executive control network for ADHD, albeit this abnormal connectivity between networks was intensively reported in previous studies. Importantly, the ADHD combined subtype showed greater consistency than did the inattention subtype. These findings provided methodological insights into the studies on spontaneous brain activity of ADHD, and the ADHD subtypes deserve more attention in future studies.

## Introduction

Attention deficit hyperactivity disorder (ADHD) is one of the most common neurodevelopmental disorders in children (1). It is a highly heterogeneous disease characterized by varying degrees of inattention, hyperactivity, and impulsivity (2, 3). The pathogenesis of ADHD is incompletely understood, and a promising trend is the application of resting-state functional magnetic resonance imaging (rs-fMRI). rs-fMRI, measuring spontaneous brain activity, is easy to be implemented. It provides a consistent approach for clinical investigations; and two major measurements, that is, voxel-wise metrics and functional connectivity, were pervasively used in the rs-fMRI investigations on ADHD.

Voxel-wise metrics mainly include amplitude of low-frequency fluctuation (ALFF) (4), regional homogeneity (ReHo) (5), and degree centrality (DC) (6). The analytic process of the three metrics is similar across studies, thus helping to identify critical regions related to ADHD across fMRI studies (7, 8). By using these metrics, abnormal activity for ADHD was identified. As compared with typical developing children (TDC), ADHD showed decreased ALFF and ReHo in the brain areas of the right inferior frontal gyrus (rIFG), sensorimotor cortex, and anterior cingulate cortex (4, 9, 10). Decreased DC for ADHD was observed in the bilateral pallidum (11). These results were usually acquired based on the data from a single dataset. Recently, our research group further examined these results on multiple datasets. Abnormal brain activities in frontal-striatal areas and frontal-parietal areas were identified through the metrics of ALFF, ReHo, and DC. Notably, none of the three metrics showed consistent results across the multiple datasets, even with a lenient threshold (*p* < 0.05, cluster size > 10 voxels) (12). These findings suggest that it is necessary to re-assess the results of abnormal spontaneous brain activity of ADHD.

Functional connectivity was one another widely used measurement in the rs-fMRI studies on ADHD. Functional connectivity was defined as the correlation between the time course of a particular brain region (seed region) and all other voxels in the brain (13), and it provides detailed information about interregional relationships. In the functional connectivity explorations, three frontal regions (dorsal anterior cingulate cortex (dACC), right middle frontal gyrus (rMFG), and rIFG) identified by Weissman et al. (14) were believed to be important for the presence of ADHD (14). The three regions exhibited negative connectivity with precuneus and posterior cingulate cortex (PCC), and ADHD adults showed decreased functional connectivity between dACC and precuneus/PCC than did TDC (15). This finding was further reproduced by an investigation on ADHD children (16), and it was also believed as one biomarker of ADHD (17). Aside from the seed-based functional connectivity, the connectivity between the default mode network (DMN) and executive control network (ECN) was discussed extensively. ECN and DMN tend to be active or inactive during cognitively demanding tasks (18). DMN consists of anterior and medial prefrontal cortex (PFC), the precuneus, and the angular gyrus (19, 20). It is active when individuals are engaged in internally focused tasks including autobiographical memory retrieval, envisioning the future, and conceiving the perspectives of others (21). ECN mainly includes lateral PFC, dorsal parietal cortex, sensorimotor cortex, subcortical areas, and the cerebellum (19, 22). This network is usually activated during the performance of externally oriented tasks, so it is also termed as task-positive network (23). ECN and DMN show negative and positive connectivity with PCC (18, 24, 25). The negative connectivity between the two networks has been widely reported, and clinical evidences indicated the methylphenidate improves the symptoms of ADHD and meanwhile increases the negative connectivity between ECN and DMN (23).

The seed-based functional connectivity and connectivity between ECN and DMN inform our understanding about the pathological mechanism of ADHD. However, to what extent the functional connectivity results are consistent across individual datasets remains unclear. To address this issue, the present study examined the abnormal functional connectivity based on the datasets of ADHD-200. ADHD-200 is one of the most widely used multi-site MRI cohorts of ADHD, and it contains 10 independent datasets from eight different sites (26). These datasets provide rs-fMRI and anatomical MRI data of both ADHD and TDC. The consistency of seed-based functional connectivity and the negative connectivity between ECN and DMN were first examined across individual datasets. Moreover, ADHD involves three subtypes, that is, inattention, hyperactivity/impulsivity, and combined (27). It was observed that children with different ADHD subtypes showed differences in spontaneous brain activity (28, 29). Thus, a single subtype may show greater consistency of abnormal spontaneous brain activity than the mixed subtypes of ADHD. Therefore, analyses based on the subtypes were also involved in the present study.

## Methods and Materials

### Participants and Data Acquisition

The data we used in this study are publicly available from the ADHD-200 Consortium (http://fcon_1000.projects.nitrc.org/indi/adhd200). The ADHD-200 cohort contains both functional and anatomical MRI data contributed by eight institutions. Each dataset was approved by the research ethics review boards of each institution. Signed informed consent was obtained from all participants or their legal guardian before participation.

**Figure 1:**
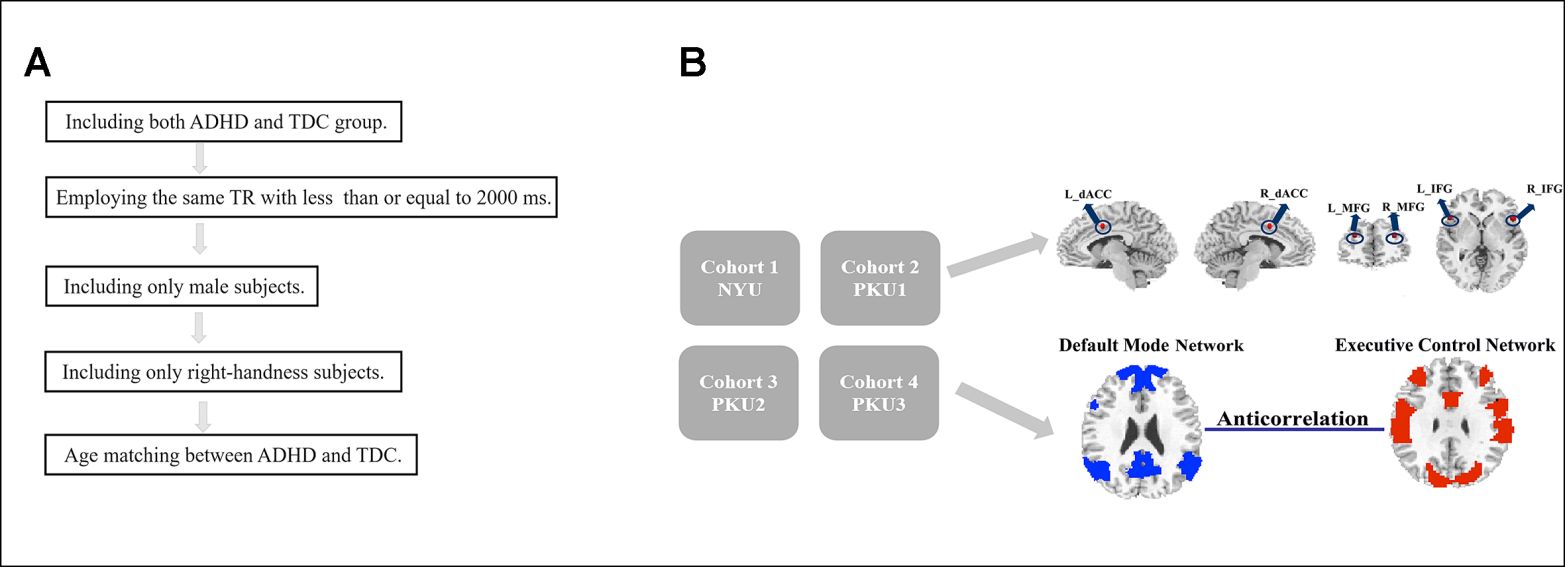
Flowchart of date exclusion **(A)** and data analysis **(B)**.

The datasets were first selected according to the following criteria (**Figure 1A**): (1) Including both ADHD and TDC groups; so the data from BU, University of Pittsburgh, and Washington University were excluded. (2) Employing the same time of repetition (TR) of ≤2,000 ms across the datasets. According to this criterion, Kennedy Krieger Institute (KKI) (TR = 2,500 ms) and Oregon Health & Science University (OHSU) (TR = 2,500 ms) were excluded. Then, the datasets of NYU, PKU1, PKU2, and PKU3 were included in the study, and data from *NeuroImage* (TR = 1,960 ms) were also excluded because the TR of *NeuroImage* was not the same as the TR of the other four datasets. The PKU2 and PKU3 datasets only had male subjects, so the female subjects in NYU and PKU1 datasets were excluded to remove potential confounding effect of gender on the results. Left-handed subjects were also excluded from each dataset. There is no significant difference in age between children with ADHD and TDC across all datasets in the present study. After case-by-case age matching between ADHD and TDC, 58 subjects from NYU, 30 from PKU1, 56 from PKU2, and 38 from PKU3 were included in the current study. Demographic information of all subjects is summarized in **Table 1**.

**Table 1:** Demographic information of each dataset in current study.

	NYU		PKU1		PKU2		PKU3	
	ADHD	TDC	ADHD	TDC	ADHD	TDC	ADHD	TDC
***N***	29	29	15	15	28	28	19	19
***Gender (male)***	29	29	15	15	28	28	19	19
***Age (year)***	12.1 ± 2.9	12.2 ± 2.8	11.2 ± 2.3	11.6 ± 1.5	12.7 ± 1.7	11.7 ± 1.8	13.2 ± 1.3	13.3 ± 1.0
***IQ***	106 ± 16.0	115.3 ± 14.3	101.7 ± 12.4	123.0 ± 14.2	111.5 ± 12.7	121.6 ± 12.2	102.7 ± 10.4	111.7 ± 12.7
***Subtype***	19/10/0	–	6/9/0	–	12/16/0	–	7/12/0	–

*Data are presented as mean ± SD. C, ADHD-combined; I, ADHD-inattention; H, ADHD-hyperactive/impulsive. ADHD, attention deficit hyperactivity disorder; TDC, typical developing children.*

Medications were withheld for at least 24 h prior to scanning. More detailed demographic characteristics of the participants of the four datasets can be seen in http://fcon_1000.projects.nitrc.org/indi/adhd200. The rs-fMRI data of the four datasets were acquired from three scanners, with TR of 2,000 ms for all. PKU1 and PKU2 used the same scanner, but scanning parameters were slightly different. The detailed parameters are listed in **Supplementary Material Table S1**.

### Data Processing

The preprocessing was carried out using the Data Processing Assistant for Resting-State fMRI (DPARSF) (30), which is based on the Statistical Parametric Mapping (SPM8) (http://www.fil.ion.ucl.ac.uk/spm) and Resting-State fMRI Data Analysis Toolkit (REST) (31) (http://www.restfmri.net). The first 10 time points were removed for signal stabilization and participant adaptation. And then, the number of left time points for NYU, PKU1, PKU2, and PKU3 is 170, 230, 230, and 230, respectively. The unified number of time points (first 170 volumes) was employed for all of the four datasets. Slice timing correction and image realignment to correct head motion were followed. The head motion criteria, that is, head motion <3 mm translation or <3° rotation in any direction, were employed, referring to previous studies (32–34), and all subjects met these criteria. Individual structural images were segmented after co-registered to functional images. Then, functional images were spatial normalized to Montreal Neurological Institute (MNI) template (re-sampled into 3 × 3 × 3 mm^3^) and smoothed with an 6 × 6 × 6 full-width-at-half-maximum (FWHM) Gaussian kernel. The head motion parameter measured by Friston-24 model, global signal effect, white matter (WM), and cerebrospinal fluid (CSF) signals were further regressed out as nuisance covariates. The time course of each voxel was linearly detrended and band-pass filtered with frequencies ranging from 0.01 to 0.08 Hz.

### Seed-Based Functional Connectivity Analyses

The functional connectivity analyses were performed based on the seed regions of interest (ROIs), including R_dACC, R_IFG, and R_MFG. The three seed regions were defined as a sphere with a 6-mm radius (34 voxels) centered at the coordinates reported by Weissman et al. (14) converted to MNI space (R_dACC: *x* = 8, *y* = 7, *z* = 38; R_IFG: *x* = 34, *y* = 45, *z* = 23; R_MFG: *x* = 49, *y* = 19, *z* = 0). Moreover, asymmetry of the human brain was mentioned intensively (35, 36), so we also performed the functional connectivity analyses in the contralateral ROIS, and the coordinates were converted to MNI space (L_dACC: *x* = −8, *y* = 7, *z* = 38; L_IFG: *x* = −34, *y* = 45, *z* = 23; L_MFG: *x* = −49, *y* = 19, *z* = 0). The mean time course of each seed region was extracted as reference time course. Then, the functional connectivity was calculated as Fisher’s *Z*-transformed Pearson correlation coefficient between the reference time course and the time course of each voxel in the brain.

### Connectivity Between ECN and DMN

A public DMN mask created by Yeo et al. (37) was employed in the analysis (37), and regions showing time course negative correlation with the DMN were identified as ECN (*p* < 0.001, Gaussian random field (GRF) corrected). Then, the mean time courses were extracted based on the ECN and DMN masks in each subject. These masks were unified for the subjects of all the datasets, and therefore, there was no network difference across the datasets. The network connectivity was further calculated through the Pearson correlation between the mean time course of DMN and the mean time course of ECN.

To validate the analysis with public network masks, we performed another network construction analysis. In accordance with previous studies (38, 39), DMN/ECN was identified as regions showing positive/negative time course correlation with PCC (*p* < 0.001, GRF corrected), and the networks were constructed for each dataset. Functional connectivity with the seed region of PCC was calculated [6-mm spherical ROI centered at MNI coordinates: *x* = 1, *y* = −55, *z* = 17, reported by Vincent et al. (40)] (see details in **Supplementary Materials 2.6**). Then, the connectivity between ECN and DMN was re-calculated and compared between ADHD group and control group in each dataset. Since the networks were constructed for each dataset, the similarities and differences of the networks across all of the four datasets were further examined by the measurement of percentage of overlap (**Figure S40** and **Table S4**). Moreover, the effects of various analytical methods on the constructed networks were further assessed by the measurement of the Dice coefficient (**Figure S41** and **Table S5**).

### Statistical Analysis

Functional connectivity maps of the seed regions of R_dACC, R_IFG, R_MFG, L_dACC, L_IFG, and L_MFG were compared between the groups of children with ADHD and TDC. Two-sample *t*-tests were performed on each dataset. The comparison result for each dataset was corrected for multiple comparisons (*p* < 0.005, GRF corrected).

At the same time, to reduce the possibility of false-negative results, a more lenient threshold (*p* < 0.05, cluster size > 10 voxels) was also used for each dataset.

We also performed the analyses of standardized effect size (SES) based on Cohen’s *d*, which is calculated in the following equation (41):

$$Cohen's\;d=\frac{{\bar{X}}_{ADHD}-{\bar{X}}_{ADHD}}{S_{ALL}}$$

(1) $$S_{ALL}=\sqrt{\frac{(n_{ADHD}-1)S_{ADHD}^{2}+(n_{TDC}-1)S_{TDC}^{2}}{n_{ADHD}+n_{TDC}\;-2}}$$

According to equation of independent two-sample *t*-test, as follows,

(2) $$t=\frac{{\bar{X}}_{ADHD}-{\bar{X}}_{TDC}}{\sqrt{\frac{(n_{ADHD}-1)S_{ADHD}^{2}+(n_{TDC}-1)S_{TDC}^{2}}{n_{ADHD}+n_{TDC}-2}(\frac{1}{n_{ADHD}}+\frac{1}{n_{TDC}})}}$$

The relationship of Cohen’s *d* and* t*-value can be obtained, as follows:

(3) $$Cohen's\;d=t\sqrt{\frac{n_{ADHD}+n_{TDC}}{n_{ADHD}\cdot n_{TDC}}}$$

According to Equation (3), we transformed* t* maps into SES map for each dataset. The same threshold was applied to the SES maps of each dataset. A high level SES of 0.8 was used, which corresponded to* t* = 3.08, 2.16, 2.96, and 2.50 (*p* = 0.003, 0.039, 0.005, and 0.017) for NYU, PKU1, PKU2, and PKU3, respectively.

To view the consistency of results, the thresholded *t* maps and SES maps were binarized and overlapped among the four datasets. The number of overlapped voxels across four and three datasets was quantified using the Dice overlap coefficient (42), where the voxel number of intersection was divided by the total voxel number of all the datasets.

As for the connectivity between ECN and DMN, the individual correlation coefficient between the time courses of two networks was transformed by Fisher’ *Z* transformation. Then, a two-sample *t*-test of the Fisher’ *Z*-transformed correlation coefficients was performed between the ADHD group and TDC group in each dataset.

### Analysis on Subtypes

In the current study, there were two ADHD subtypes in all of the four datasets, that is, the ADHD combined subtype and the ADHD inattention subtype. The information on subtypes was provided by ADHD-200 directly. According to the diagnostics illustration provided by ADHD-200 (http://fcon_1000.projects.nitrc.org/indi/adhd200/), PKU1, PKU2, and PKU3 used the ADHD Rating Scale (ADHD-RS) IV to determine the ADHD subtypes, and NYU used Conners’ Parent Rating Scale-Revised, long version (CPRS-LV), to identify the ADHD subtypes. There were 19, 6, 12, and 7 participants with the ADHD combined subtype, and 10, 9, 16, and 12 participants with the ADHD inattention subtype in NYU, PKU1, PKU2, and PKU3, respectively. Each subtype matched the health control with age. All the above analysis procedures were repeated based on the data of each ADHD subtype.

## Results

### Seed-Based Functional Connectivity Across Datasets

**Figure 2:**
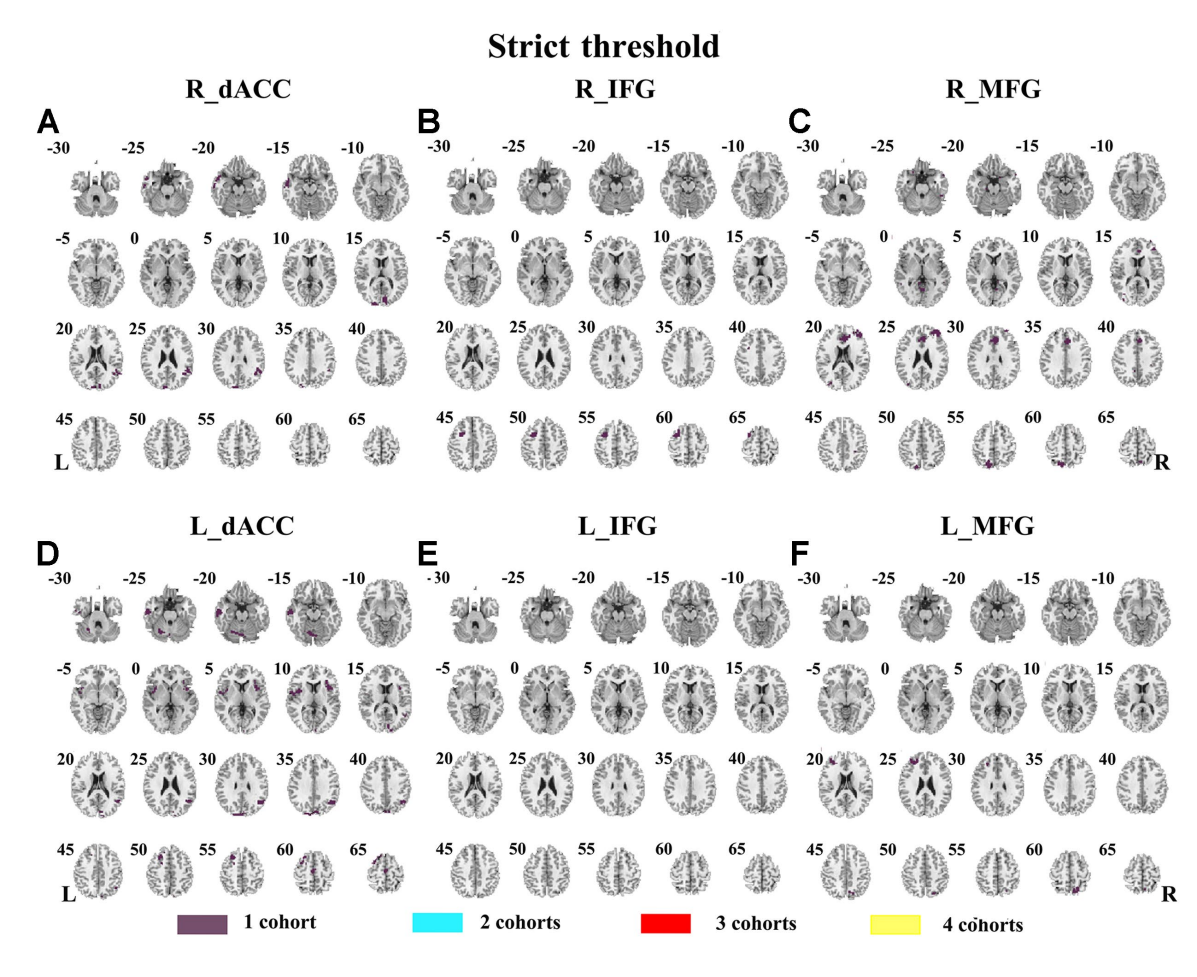
The overlapped results of abnormal functional connectivity for ADHD across the four datasets. The functional connectivity was assessed with the seed regions of R_dACC, R_IFG, R_MFG, L_dACC, L_IFG, and L_MFG. **(A–F)** indicate the results with stringent (*p* < 0.005, GRF corrected). Purple indicates the regions detected in only one of the four datasets. Mint, red, and yellow indicate the regions detected in two, three, and four datasets, respectively. ADHD, attention deficit hyperactivity disorder; dACC, dorsal anterior cingulate cortex; IFG, inferior frontal gyrus; MFG, middle frontal gyrus; GRF, Gaussian random field.

**Figure 3:**
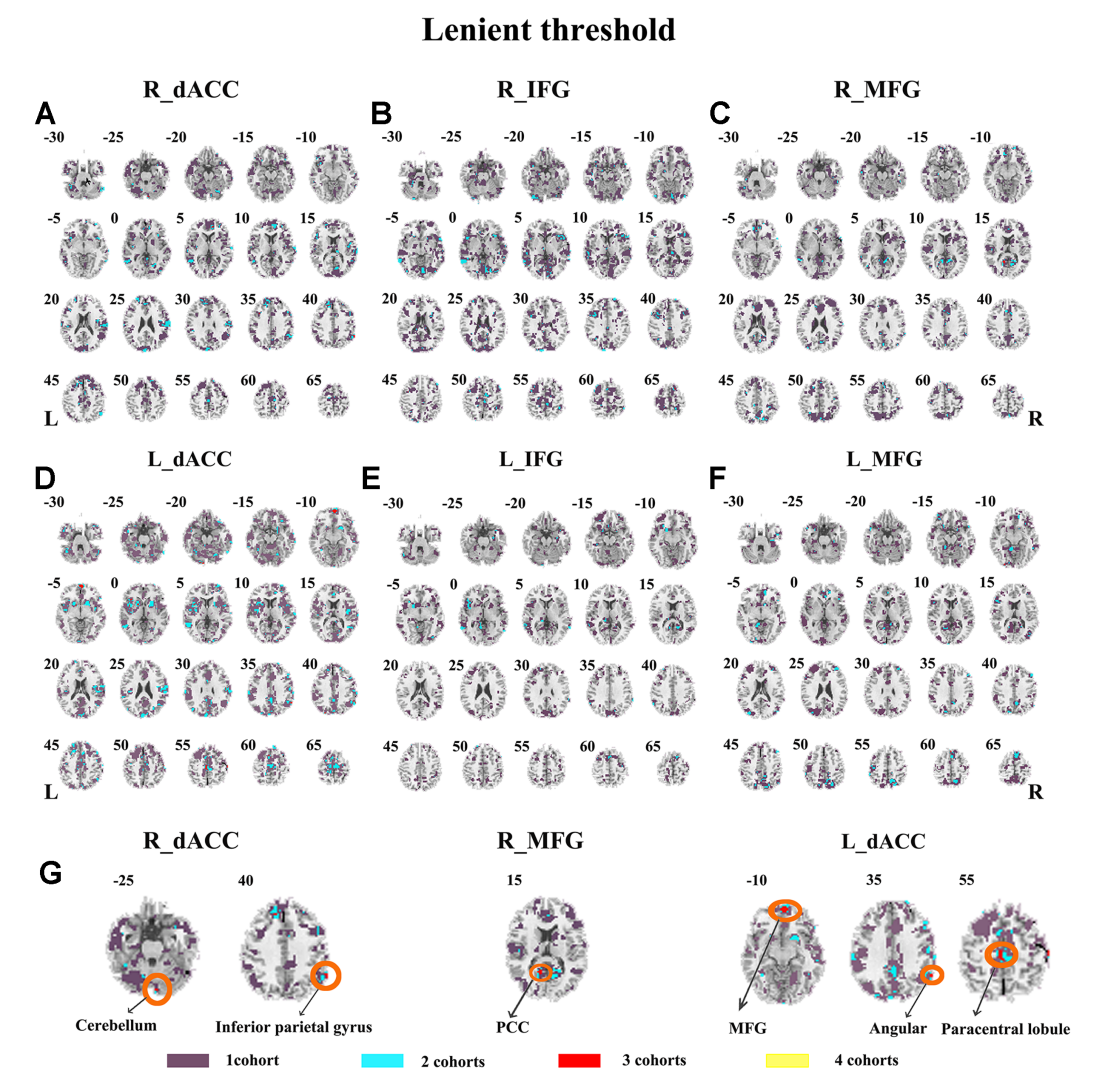
The overlapped results of abnormal functional connectivity for ADHD across the four datasets. The functional connectivity was assessed with the seed regions of R_dACC, R_IFG, R_MFG, L_dACC, L_IFG, and L_MFG. **(A–G)** indicate the results with lenient (*p* < 0.05, cluster size > 10) thresholds. Purple indicates the regions detected in only one of the four datasets. Mint, red, and yellow indicate the regions detected in two, three, and four datasets, respectively. ADHD, attention deficit hyperactivity disorder; dACC, dorsal anterior cingulate cortex; IFG, inferior frontal gyrus; MFG, middle frontal gyrus.

Abnormal seed-based functional connectivity examined in each dataset is shown in **Supplementary Material Figures S1–S6**, and the overlapped results across the four datasets for each seed region are shown in **Figure 2**. No overlapped abnormal functional connectivity was observed from three or four datasets. Even using a more lenient threshold, several voxels showed overlapped abnormal functional connectivity from three or four datasets. Using R_dACC as the seed, we observed seven overlapped voxels of NYU, PKU1, and PKU3 in the left cerebellum. Using R_MFG as the seed, we observed that 13 voxels overlapped from NYU, PKU1, and PKU3 in the PCC. When using L_dACC as the seed, we observed 26 overlapped voxels of NYU, PKU1, and PKU2 in the right medial orbital frontal gyrus and 16 voxels in the left paracentral lobule (see details in **Figure 3** and **Table 2**).

**Table 2:** Clusters showing overlaps for three/four datasets and contained maximal of overlapped voxels.

Seed	Number of overlapped cohorts	Region	L/R	BA	Number of overlapped voxels	Dice
R_dACC	3	Cerebellum	R	–	2	0.0002
		Inf. parietal gyrus	R	40	4	0.0003
R_MFG	3	Post. cingulate cortex	L	29	13	0.0050
L_dACC	3	Med. Orb. frontal gyrus	L/R	10/11	26	0.0057
		Angular	R	40	5	0.0011
		Paracentral lobule	L	6	16	0.0034

*Med., medial; Inf., inferior; Post., posterior; L, left; R, right; dACC, dorsal anterior cingulate cortex; IFG, inferior frontal gyrus; MFG, middle frontal gyrus. The threshold was p < 0.05, and cluster size > 10 voxels for each cohort.*

**Figure 4:**
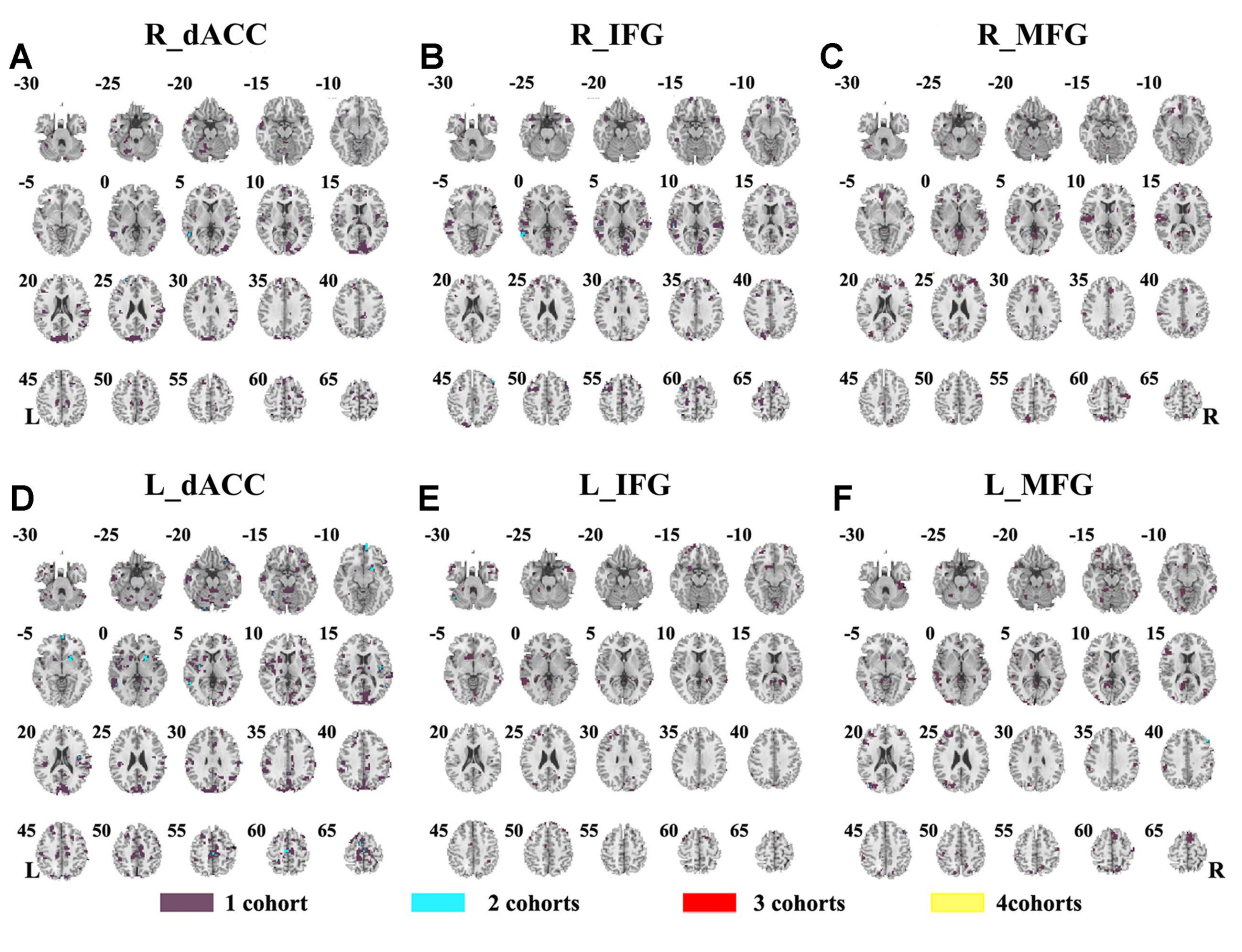
The overlapped effect size results of the individual dataset. The threshold of effect size was set at 0.8 for each dataset. **(A–F)** indicate the results by using R_dACC, R_IFG, R_MFG, L_dACC, L_IFG, and L_MFG as the seed regions, respectively. Purple indicates the regions detected only in one of the four datasets. Mint indicates the regions detected in two datasets. Red indicates the regions detected by only three datasets. Yellow indicates the regions detected in four datasets. dACC, dorsal anterior cingulate cortex; IFG, inferior frontal gyrus; MFG, middle frontal gyrus.

The SES maps of each dataset are shown in **Supplementary Material Figures S7–S12**, and the overlapped SES maps across datasets are shown in **Figure 4** (with SES > 0.8). No clusters showed overlaps from more than three datasets.

The seed-based functional connectivity was further examined in subtypes of ADHD, including the combined subtype and the inattention subtype. Abnormal functional connectivity of each dataset is shown in **Supplementary Material Figures S13–S24**. For the combined subtype, abnormal connectivity between R_IFG and middle frontal gyrus showed consistency across all datasets. This consistency was not identified in the inattention subtype (**Figures 5** and **6** and **Table 3**).

**Figure 5:**
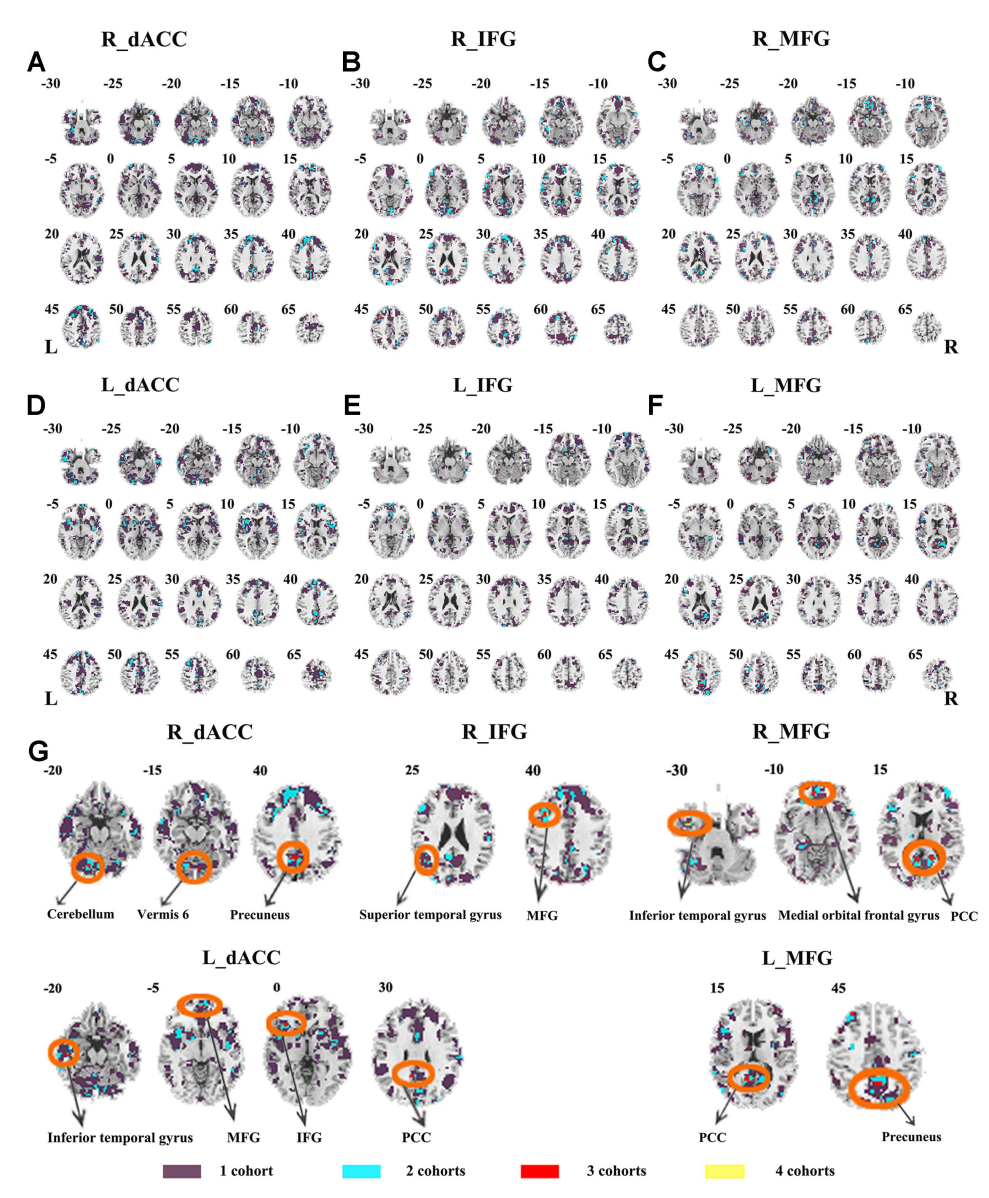
The overlapped results across the four datasets in the ADHD combined subtype. **(A–G)** indicate the results detected by using R_dACC, R_IFG, R_MFG, L_dACC, L_IFG, and L_MFG as the seed regions. Purple indicates regions detected in only one of the four datasets. Mint, red, and yellow indicate the regions detected in two, three, and four datasets, respectively. ADHD, attention deficit hyperactivity disorder; dACC, dorsal anterior cingulate cortex; IFG, inferior frontal gyrus; MFG, middle frontal gyrus.

**Figure 6:**
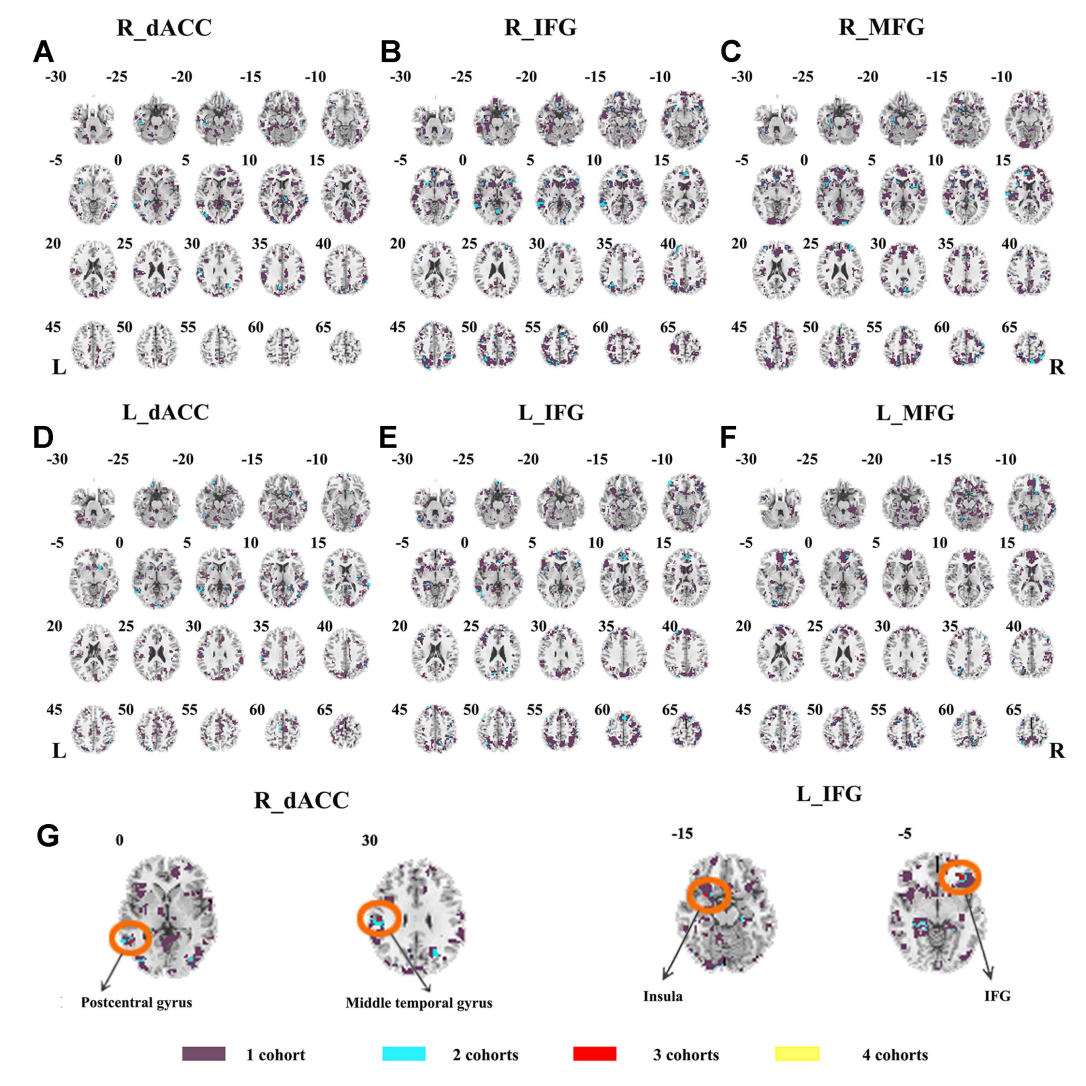
The overlapped results across the four datasets in the ADHD inattention subtypes. **(A–G)** indicate the results detected by using R_dACC, R_IFG, R_MFG, L_dACC, L_IFG, and L_MFG as the seed regions. Purple indicates regions detected in only one of the four datasets. Mint, red, and yellow indicate the regions detected in two, three, and four datasets, respectively. ADHD, attention deficit hyperactivity disorder; dACC, dorsal anterior cingulate cortex; IFG, inferior frontal gyrus; MFG, middle frontal gyrus.

**Table 3:** Clusters that were the overlap for three/four datasets and contained maximal of overlapped voxels.

Seed	Number of overlapped cohorts	Region	L/R	BA	Number of overlapped voxels	Dice
ADHD combined subtype						
R_dACC	3	Cerebellum	L	–	14	0.0035
		Vermis 6	–	–	7	0.0006
		Precuneus	L	7	14	0.0032
R_IFG	4	Mid. frontal gyrus	L	9	5	0.0015
	3	Sup. temporal gyrus	L	9	9	0.0029
		Mid. frontal gyrus	L	9	46	0.0011
R_MFG	4	Inf. temporal gyrus	L	20	2	0.0008
	3	Med. orbital frontal gyrus	L	11	29	0.0101
		Post. cingulate cortex	L/R	30	40	0.0046
L_dACC	3	Inf. temporal gyrus	L	20	6	0.0016
		Med. frontal gyrus	L	11	13	0.0035
		Inf. frontal gyrus	L	45	9	0.0024
		Post. cingulate cortex	L	30	12	0.0029
L_MFG	3	Post. cingulate cortex	L	30	16	0.0060
		Precuneus	R	7	8	0.0030
**ADHD inattention subtype**						
R_dACC	3	Mid. temporal gyrus	L	39	5	0.0008
		Postcentral gyrus	L	2	5	0.0023
L_IFG	3	Inf. frontal gyrus	R	45	5	0.0020
		Insula	L	38	1	0.0001

*Med., medial; Mid., middle; Inf., inferior; Sup., superior; Post., posterior; L, left; R, right; dACC, dorsal anterior cingulate cortex; IFG, inferior frontal gyrus; MFG, middle frontal gyrus; ADHD, attention deficit hyperactivity disorder. The threshold was p < 0.05, and cluster size > 10 voxels.*

The SES maps of each dataset/each ADHD subtype are shown in **Supplementary Material Figures S25–S36**. For each ADHD subtype, the overlapped SES maps across all datasets are shown in **Figures 7** and **8**. Abnormal functional connectivity of each seed region showed overlaps from more than three datasets, and these overlaps were only observed in the combined subtype but not in the inattention subtype.

**Figure 7:**
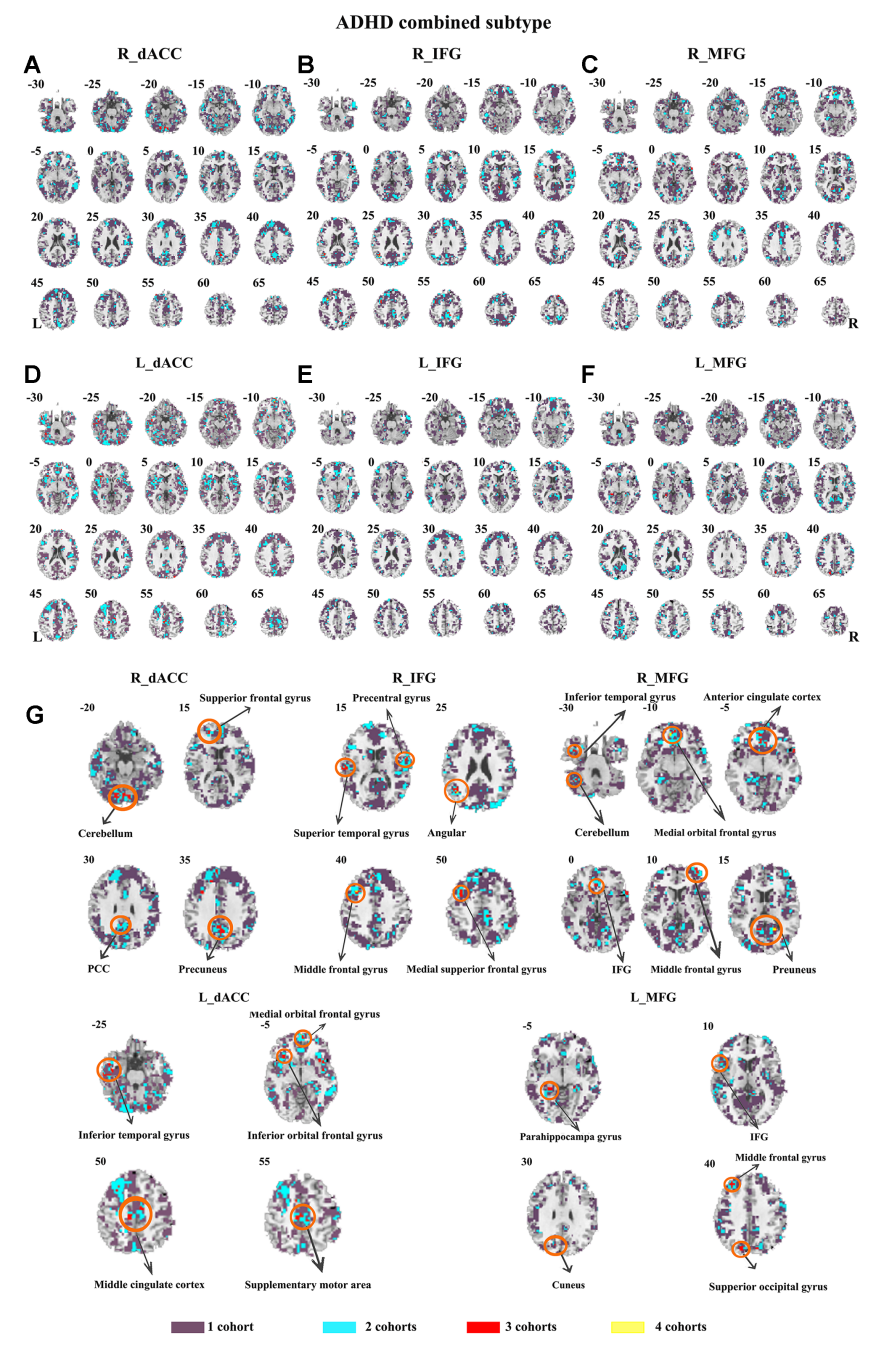
The overlapped effect size results of the individual dataset in the ADHD combined subtypes. The threshold of effect size was set at 0.8 for each dataset. **(A–G)** indicate the results detected by using R_dACC, R_IFG, R_MFG, L_dACC, L_IFG, and L_MFG as the seed regions. Purple indicates the regions detected only in one of the four datasets. Mint indicates the regions detected in two datasets. Red indicates the regions detected by only three datasets. Yellow indicates the regions detected in four datasets. ADHD, attention deficit hyperactivity disorder; dACC, dorsal anterior cingulate cortex; IFG, inferior frontal gyrus; MFG, middle frontal gyrus.

**Figure 8:**
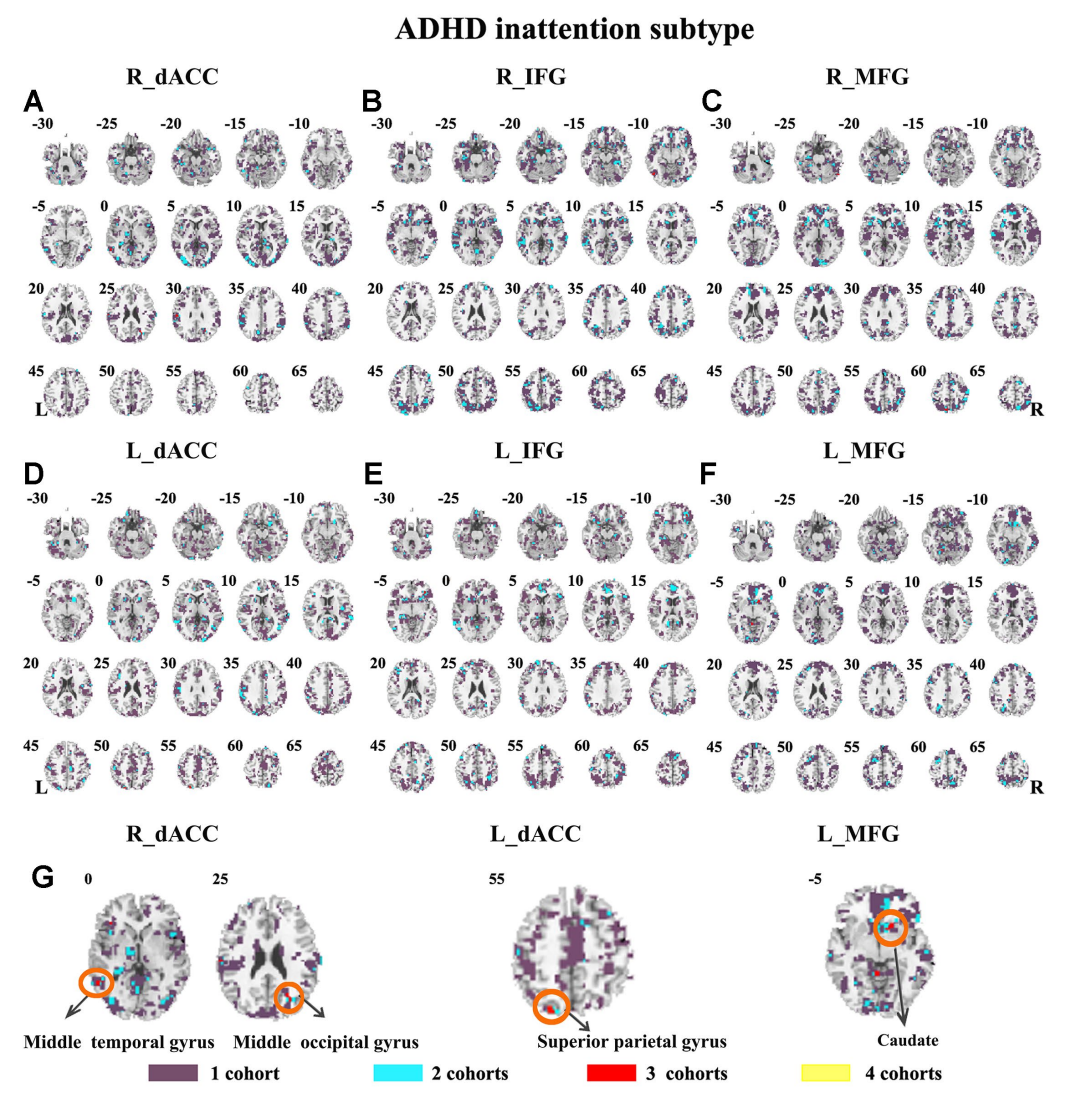
The overlapped effect size results of the individual dataset in the ADHD inattention subtypes. The threshold of effect size was set at 0.8 for each dataset. **(A–G)** indicate the results detected by using R_dACC, R_IFG, R_MFG, L_dACC, L_IFG, and L_MFG as the seed regions. Purple indicates the regions detected only in one of the four datasets. Mint indicates the regions detected in two datasets. Red indicates the regions detected by only three datasets. Yellow indicates the regions detected in four datasets. ADHD, attention deficit hyperactivity disorder; dACC, dorsal anterior cingulate cortex; IFG, inferior frontal gyrus; MFG, middle frontal gyrus.

Considering that the global signal effect on the functional connectivity analysis is still controversial, we also repeated the above analysis based on the preprocessed data without removing the global signal effect. The results are shown in the **Supplementary Materials (Figures S42–S48)**, which also exhibited few overlaps of the seed-based functional connectivity across the four datasets.

### Connectivity Between ECN and DMN

**Figure 9:**
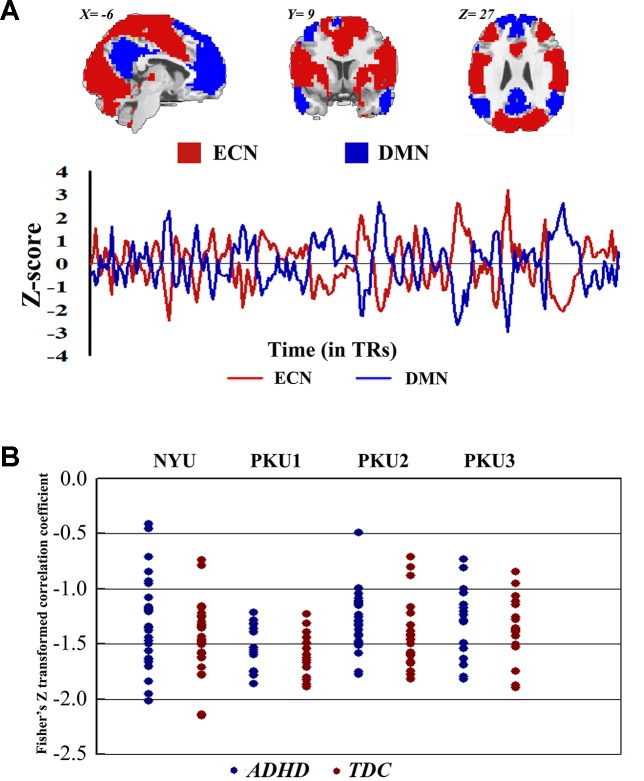
The Fisher *Z*-transformed negative connectivity between ECN and DMN of ADHD group and TDC group in each dataset. **(A)** The schematic diagram of ECN and DMN masks used in the analysis, and the *Z*-score graph indicated the antiphase time course of ECN and DMN for one subject. **(B)** Individual negative connectivity between ECN and DMN in each dataset. ECN, executive control network; DMN, default mode network; ADHD, attention deficit hyperactivity disorder; TDC, typical developing children.

Both children with ADHD and TDC in each dataset showed strong negative connectivity between ECN and DMN (**Figure 9**). No significant difference of this negative connectivity was observed between children with ADHD and TDC in each dataset (**Table 4**). This finding was reproduced by our further validation analysis with another network construction method (see details in **Supplementary Material Figure S37** and **Table S2**).

**Table 4:** The statistical difference of the negative network connectivity between ADHD group and TDC group.

Dataset	ADHD group *Mean ± SD*	TDC group *Mean ± SD*	*t*	*p*
NYU	−1.31 ± 0.40	−1.45 ± 0.31	1.54	0.13
PKU1	−1.51 ± 0.21	−1.60 ± 0.20	1.24	0.23
PKU2	−1.30 ± 0.26	−1.41 ± 0.27	1.57	0.12
PKU3	−1.30 ± 0.30	−1.36 ± 0.28	0.66	0.51

*No significant difference could be preserved after the multiple comparison correction. ADHD, attention deficit hyperactivity disorder; TDC, typical developing* *children.*

**Figure 10:**
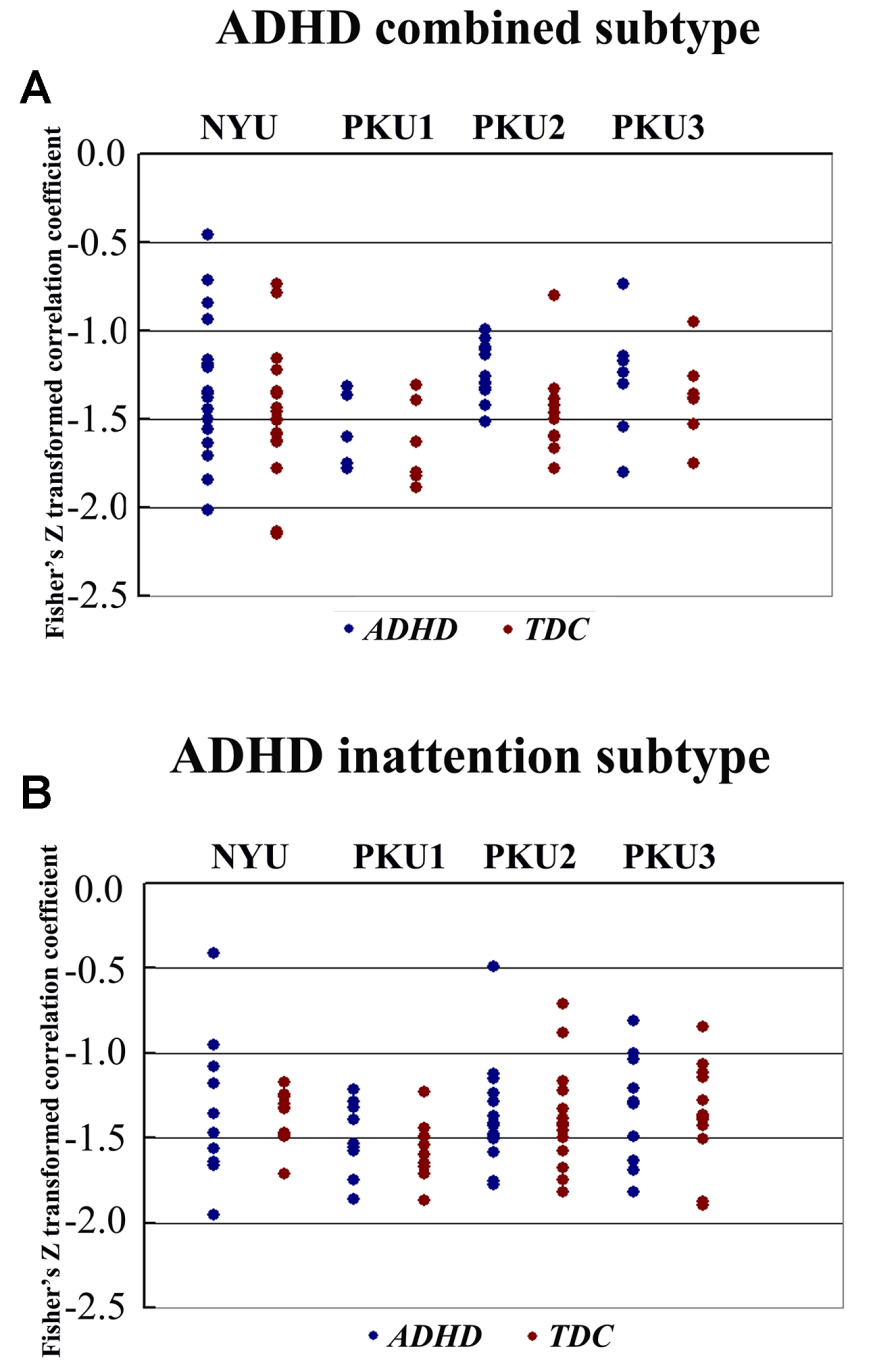
The Fisher *Z* score of negative connectivity results between ADHD group and TDC group of each dataset in the ADHD combined subtype **(A)** and the ADHD inattention subtype **(B)** with public DMN public mask. ADHD, attention deficit hyperactivity disorder; TDC, typical developing children; DMN, default mode network.

We also analyzed the negative connectivity between ECN and DMN in ADHD subtypes. As **Figure 10** shows, this negative connectivity in each dataset did not show significant difference between the children with the inattention subtype and TDC. Children with the combined subtype showed the trend of significant difference between ADHD and TDC in PKU2, *t*(22) = 2.69, *p* = 0.01 (**Figure 10** and **Table 5**); however, these results could not survive after the multiple comparison correction.

**Table 5:** The statistical difference of the negative network connectivity between ADHD subtype group and TDC group.

Dataset	ADHD group Mean ± SD	TDC group Mean ± SD	*t*	*p*
ADHD combined subtype				
**NYU**	−1.30 ± 0.39	−1.49 ± 0.36	1.60	0.12
**PKU1**	−1.53 ± 0.21	−1.64 ± 0.24	0.86	0.41
**PKU2**	−1.23 ± 0.16	−1.46 ± 0.25	2.69	0.01
**PKU3**	−1.28 ± 0.33	−1.37 ± 0.24	0.61	0.55
**ADHD inattention subtype**				
**NYU**	−1.33 ± 0.44	−1.38 ± 0.16	0.34	0.74
**PKU1**	−1.50 ± 0.22	−1.58 ± 0.18	0.84	0.41
**PKU2**	−1.36 ± 0.30	−1.38 ± 0.29	0.21	0.83
**PKU3**	−1.31 ± 0.30	−1.36 ± 0.31	0.34	0.73

*No significant difference could be preserved after the multiple comparison correction. ADHD, attention deficit hyperactivity disorder; TDC, typical developing children.*

These results could be reproduced in our validation analysis with another network construction method (details in **Supplementary Material Figures S38** and **S39** and **Table S3**). However, we failed to identify the negative connectivity between ECN and DMN when reproducing the results based on the preprocessed data without removing the global signal effect (**Supplementary Material Figures S49–S54** and **Tables S6–S9**).

## Discussion

The current study examined the consistency of abnormal functional connectivity across datasets of ADHD-200. We employed seed-based functional connectivity, the negative connectivity between ECN and DMN, stringent and lenient statistical thresholds, and ADHD subtypes in the analysis process. Three major results were obtained: (1) The abnormal seed-based functional connectivity of ADHD was not consistent across datasets. (2) For each dataset, the negative connectivity between ECN and DMN did not show any significant difference between ADHD and TDC. (3) In a subtype analysis, the combined subtype showed more consistent results across datasets as compared with the inattention subtype.

As previous studies reported, functional connectivity between PCC/precuneus and the three regions of R_dACC, R_IFG, and R_MFG could be observed in children with ADHD and TDC (15, 43), and the decreased functional connectivity between dACC and PCC/precuneus was intensively reported in rs-fMRI studies on ADHD (16, 44, 45). In the present study, this abnormal functional connectivity was not consistent across all datasets. Decreased connectivity between R_dACC and PCC/precuneus was observed only in the datasets of NYU and PKU1. The abnormal functional connectivity showed some overlaps from three datasets in the region of right inferior parietal gyrus, right supplementary motor area (SMA), and so on. However, the highest Dice overlap coefficient is just 0.0057 even when using a lenient statistical threshold (*p* < 0.05, cluster size > 10 voxels). Notably, the inconsistency could also be observed in three datasets from the same research site, PKU. Furthermore, in each dataset, the SES of the abnormal connectivity was small, and no overlaps of the SES maps were observed from more than three datasets. These findings suggest high inconsistency of abnormal functional connectivity in children with ADHD, and such inconsistency may be related to the heterogeneity of ADHD (2, 46). It is important to further validate the findings of functional connectivity in children with ADHD, and at least, both statistical and SES results should be provided in future studies.

Negative connectivity between ECN and DMN was widely examined, and it was believed as the toggling between the executive state and introspective state (25, 47, 48). In the present study, each subject showed strong negative connectivity between ECN and DMN. Such negative connectivity has attracted more attention from ADHD research communities for its correlation with the stability of behavior (49). Clinically, this negative connectivity was decreased in children with ADHD without medicine and was increased after use of methylphenidate (23). In the present study, no significant difference of this negative connectivity was identified between children with ADHD and TDC in each dataset. This finding could be validated by different network construction methods. The networks constructed by the data of individual dataset were different across all of four datasets (the percentage of common region from 51.67% to 66.60%). Moreover, these networks showed difference of spatial pattern as compared with the networks provided by the previous study (37) (Dice coefficient from 0.58 to 0.63). The difference of network spatial pattern may affect the results of the connectivity between networks (50). Thus, we directly employed the networks of Yeo et al. (37) to further validate the negative functional connectivity between ECN and DMN, and the findings were not changed. Moreover, we observed the trend of significant difference when performing analysis in the ADHD subtypes. Children with the combined subtype showed the trend of significant difference between ADHD and TDC in PKU2; however, these results could not survive after the multiple comparison correction. So these results were not enough to support the biomarker role of this negative connectivity for the presence of ADHD.

The ADHD involve three subtypes, that is, inattention, hyperactivity/impulsivity, and combined (27). It was suggested that the mixed ADHD subtypes may have confounding effects on the results of abnormal spontaneous brain activity (29). Thus, ADHD subtype was considered as a factor in the analysis process. Here, only the combined subtype and the inattention subtype were involved in the datasets. Results from the combined subtype showed more consistency than did the inattention subtype, and combined subtype showed more overlapped regions across more than three datasets. Specifically, children with the combined subtype showed increased functional connectivity between R_IFG and middle frontal gyrus in all of four datasets. Moreover, considering that the sample size may affect the results, we further calculated the effect size. Also, with high SES (> 0.8), combined subtype exhibited overlapped regions from three datasets. These overlapped regions, such as IFG, MFG, precuneus, PCC, cerebellum, and SMA, have been reported by previous studies on ADHD. For example, Maarten et al. (51) found that increased positive connectivity between SMA and MFG was associated with inhibitory function (51). Duann et al. (52) also found that the greater connectivity between IFG and SMA is related to successful response inhibition (52). Rubia et al. (53) reported that the negative correlation in ADHD patients between reduced activation in PCC and hyperactivity scale scores confirms a relationship between behavioral neural abnormalities and impulsiveness (53). So these overlapped regions may be related to the impulsiveness and inhibition. By contrast, children with the inattention subtype did not show any overlaps of abnormal functional connectivity across more than three cohorts, considering that the ADHD combined subtype included symptoms of hyperactivity/impulsivity and inattention. Thus, the consistency of abnormal functional connectivity for ADHD combined subtype may be not related to inattention symptom in ADHD.

Several limitations exist in the present study. First, we explored the functional connectivity with the seed regions of R_dACC, R_IFG, R_MFG, L_dACC, L_IFG, and L_MFG and the negative connectivity between ECN and DMN. Thus, our results were restricted to these measurements, which could not be extended to other regions and networks. Second, only ADHD combined subtype and inattention subtype were involved in the present study. We did not perform an analysis on the data of hyperactivity/impulsivity subtype because datasets of ADHD-200 only include nine subjects with this subtype. Further studies recruiting subjects with hyperactivity/impulsivity are helpful for the validation of our findings. Lastly, we explored the contribution of different subtypes to the inconsistency in ADHD neuroimaging findings; however, the sample size for statistical analysis was too small. For example, PKU1 only included nine ADHD inattention subtype subjects and six ADHD combined subtype subjects. Their contribution should be further explored on a large sample dataset in the future.

## Conclusions

Functional connectivity provided profound information for us to understand the pathological mechanism of ADHD. This is the first study, to our knowledge, to assess the consistency of abnormal functional connectivity in children with ADHD across different datasets. We found that the results of abnormal functional connectivity were inconsistent across datasets, even across three datasets from the same research site. And there was no significant difference between ADHD and TDC in the negative connectivity between ECN and DMN. More importantly, abnormal functional connectivity of the combined subtype was more consistent than that of the inattention subtype. These results provided methodological implications for the rs-fMRI studies of children with ADHD, and subtype should be involved in the analysis as a critical factor in the future studies.

## Data Availability Statement

Publicly available datasets were analyzed in this study. This data can be found here: http://fcon_1000.projects.nitrc.org/indi/adhd200.

## Ethics Statement

Signed informed consent was obtained from all participants or their legal guardian before participation.

## Author Contributions

HZ and Y-FZ conceived and designed the experiment. Z-WZ and X–QL performed the data analysis. Y-TF, HL, Q-JC, LS and Y-FW provided advice on the analysis and interpretation of the results. Z-WZ and HZ wrote the paper.

## Funding

This work is supported by the National Key R&D Program of China (2018YFC1312600 and 2018YFC1312603), the National Natural Sciences Foundation of China (31471084, 81520108016, 81471382, 81873804, and 81771479), the National Basic Research Program of China (973 program 2014CB846104), and Zhejiang Fundamental Public Welfare Research Program (LGJ19C090001). HZ is supported by Scientific Research Staring Foundation from Hangzhou Normal University.

## Conflict of Interest

The authors declare that the research was conducted in the absence of any commercial or financial relationships that could be construed as a potential conflict of interest.
