# Supplementary material for: Inconsistency in Abnormal Functional Connectivity Across Datasets of ADHD-200 in Children With Attention Deficit Hyperactivity Disorder
Source: Front Psychiatry. 2019 Sep 27;10:692. doi: 10.3389/fpsyt.2019.00692 (PMC6777421; doi:10.3389/fpsyt.2019.00692)
Supplement: Supplementary file 1 [file DataSheet_1.pdf]

Supplementary Materials

1. Scanning Parameters.

Table S1. The parameters of T1-weighted structural image can be found in http://fcon_1000.projects.nitrc.org/indi/adhd200/.

Sites	TR/TE/Flip Angle	Slice thickness/Gap	FOV	Slice	TP	Duration
**NYU**	2000 ms/15 ms/90°	4.0 mm/0 mm	240×192mm	33	176	6 min
**PKU1**	2000 ms/30 ms/90°	3.5 mm/0.7 mm	200×200mm	33	236	8 min
**PKU2**	2000 ms/30 ms/90°	3.0 mm/0.6mm	200×200mm	33	236	8 min
**PKU3**	2000 ms/30 ms/90°	4.5 mm/0 mm	220×220mm	30	336	8 min

2. Results for the analyses with removing global signal effect

2.1 The difference of seed-based functional connectivity between children with ADHD and TDC

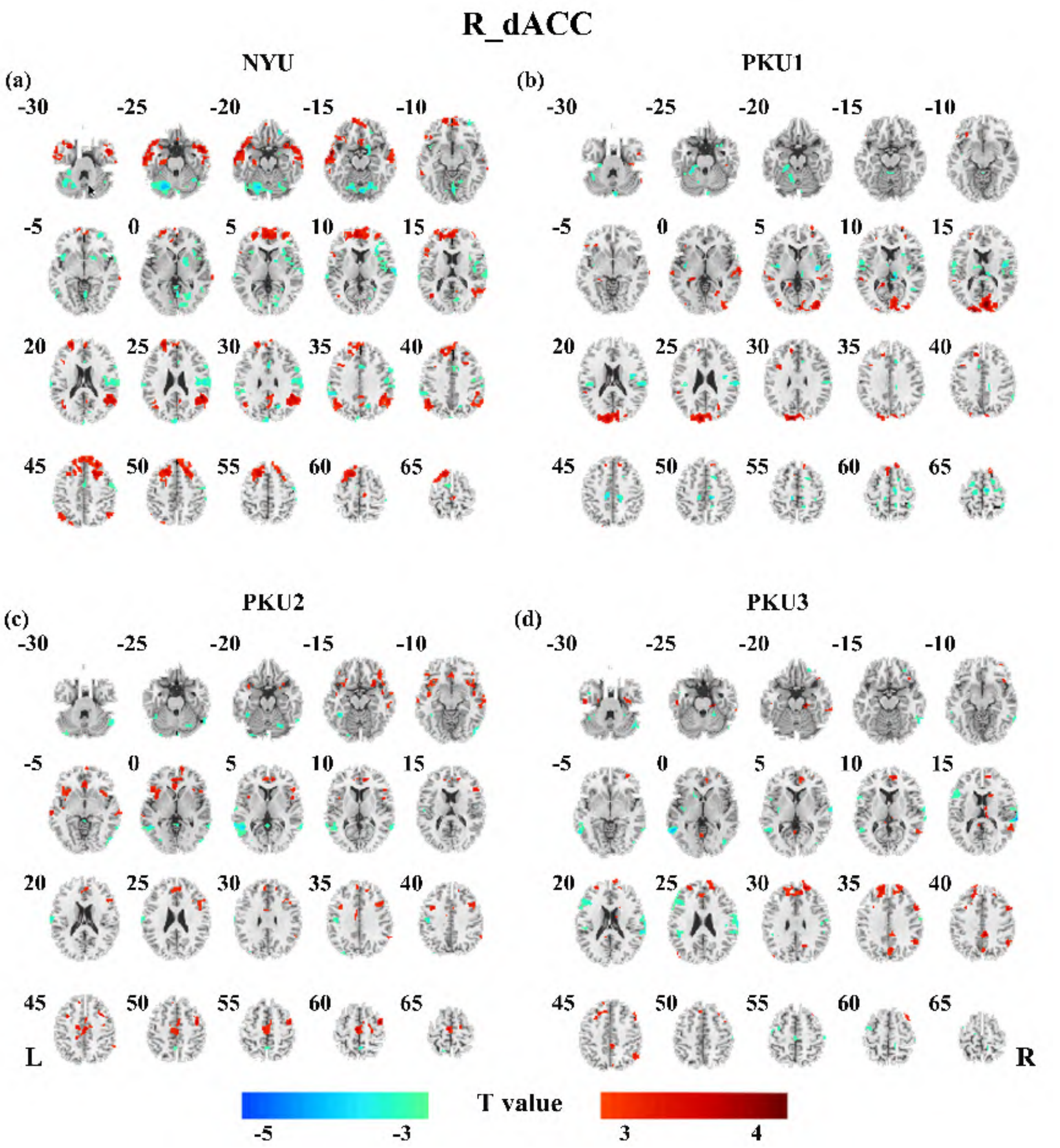

Figure S1. Clusters showing significant functional connectivity differences between children with ADHD and TDC. a-d indicate the result of NYU, PKU1, PKU2 and PKU3, respectively. Cold color indicates the region in which ADHD subjects had decreased functional connectivity with R_dACC compared with TDC and the warm color indicates the opposite ($p < 0.05$, cluster size > 10).

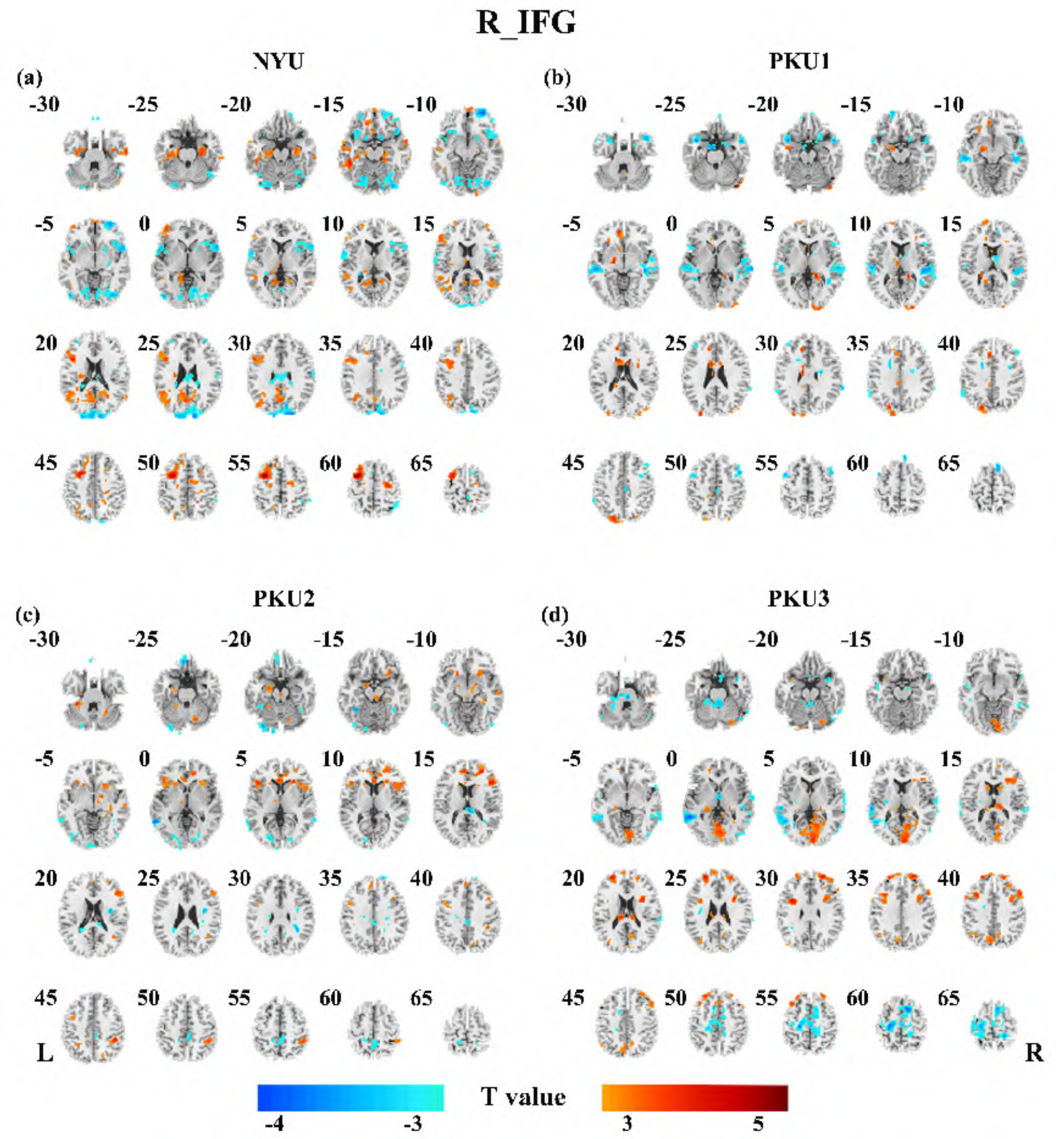

Figure S2. Clusters showing significant functional connectivity differences between children with ADHD and TDC. a-d indicate the result of NYU, PKU1, PKU2 and PKU3, respectively. Cold color indicates the region in which ADHD subjects had decreased functional connectivity with R_IFG compared with TDC and the warm color indicates the opposite ($p < 0.05$, cluster size > 10).

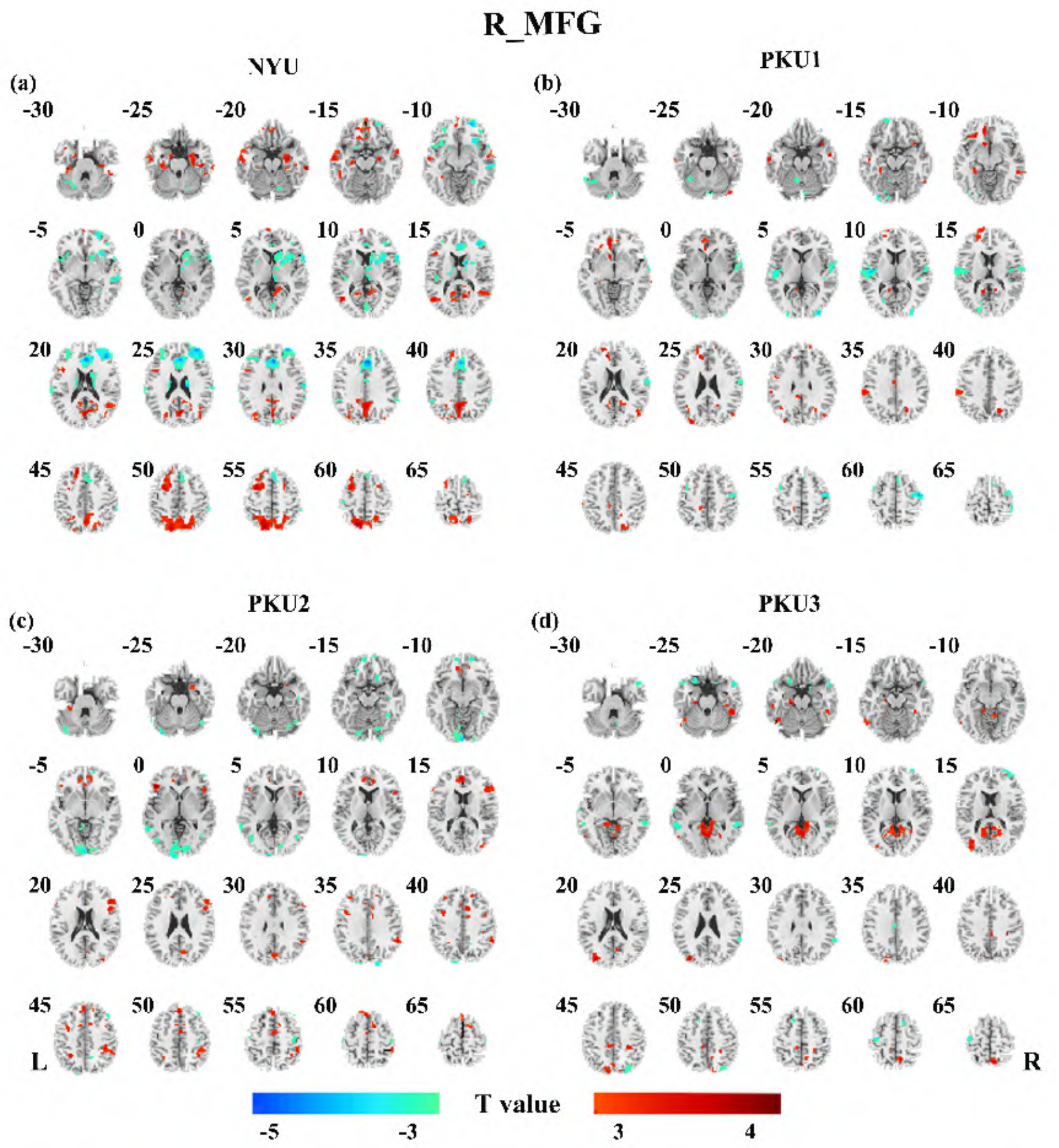

Figure S3. Clusters showing significant functional connectivity differences between children with ADHD and TDC. a-d indicate the result of NYU, PKU1, PKU2 and PKU3, respectively. Cold color indicates the region in which ADHD subjects had decreased functional connectivity with R_MFG compared with TDC and the warm color indicates the opposite ($p < 0.05$, cluster size > 10).

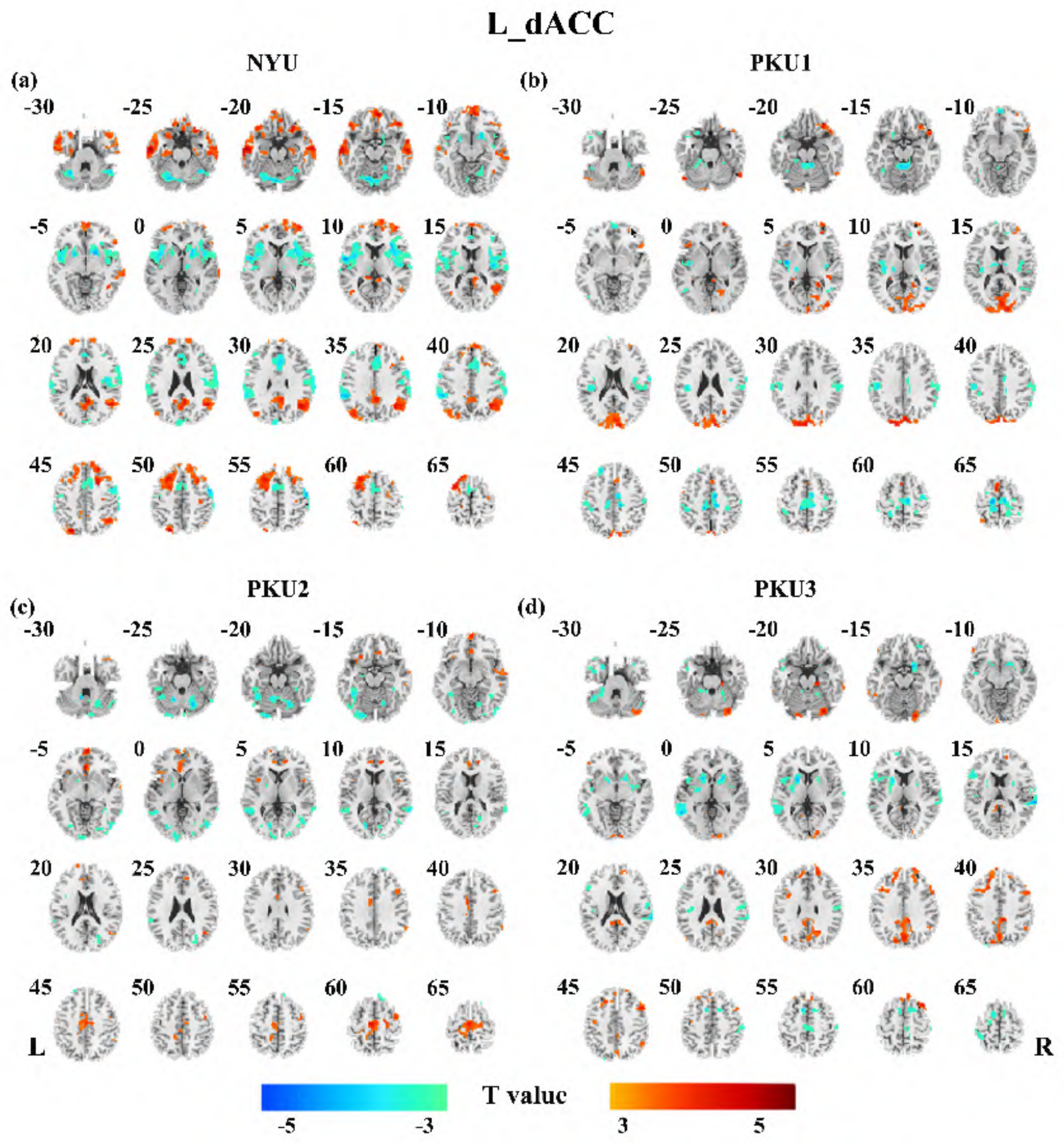

Figure S4. Clusters showing significant functional connectivity differences between children with ADHD and TDC. a-d indicate the result of NYU, PKU1, PKU2 and PKU3, respectively. Cold color indicates the region in which ADHD subjects had decreased functional connectivity with L_dACC compared with TDC and the warm color indicates the opposite ($p < 0.05$, cluster size > 10).

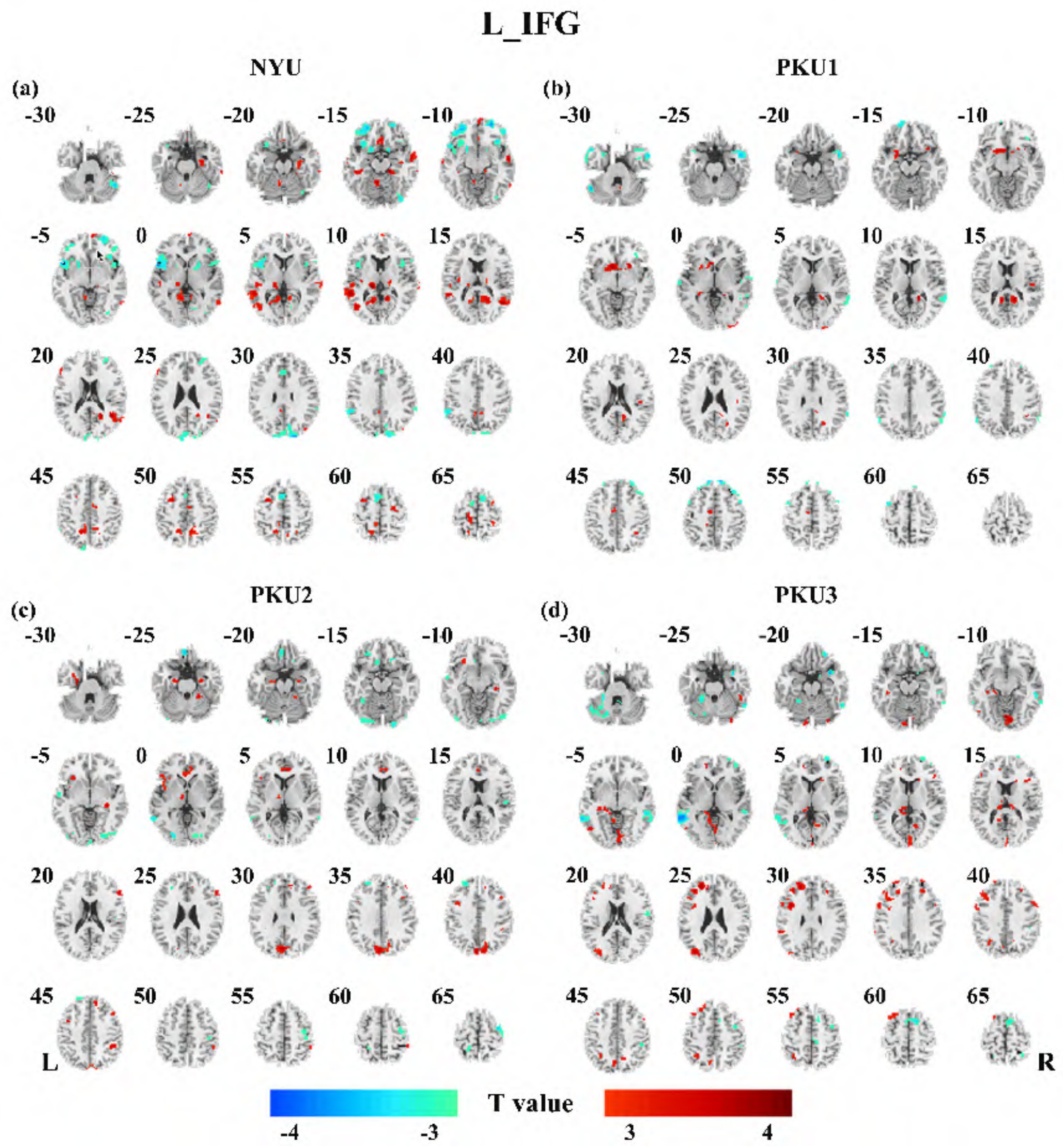

Figure S5. Clusters showing significant functional connectivity differences between children with ADHD and TDC. a-d indicate the result of NYU, PKU1, PKU2 and PKU3, respectively. Cold color indicates the region in which ADHD subjects had decreased functional connectivity with L_IFG compared with TDC and the warm color indicates the opposite ($p < 0.05$, cluster size > 10).

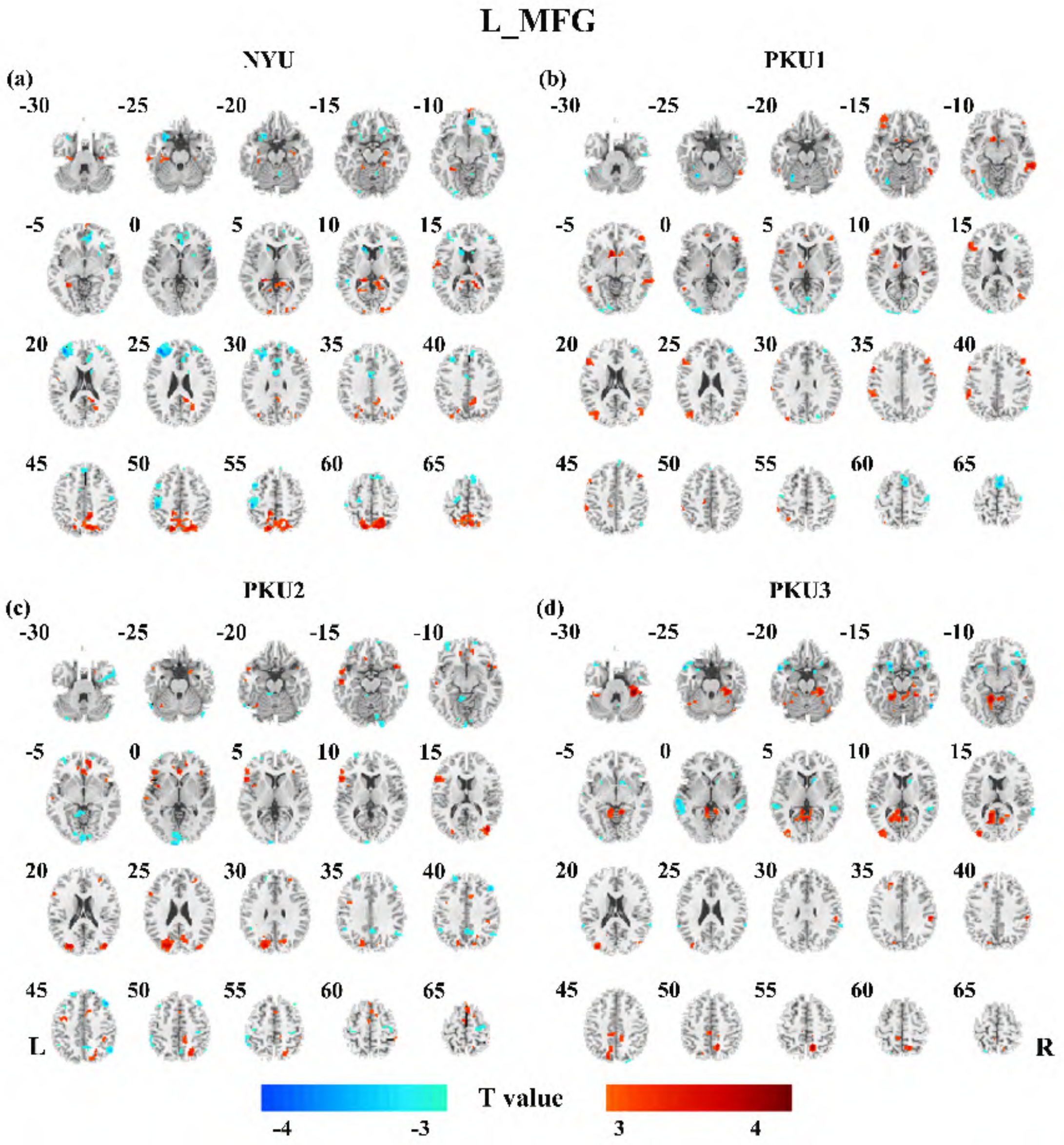

Figure S6. Clusters showing significant functional connectivity differences between children with ADHD and TDC. a-d indicate the result of NYU, PKU1, PKU2 and PKU3, respectively. Cold color indicates the region in which ADHD subjects had decreased functional connectivity with L_MFG compared with TDC and the warm color indicates the opposite ($p < 0.05$, cluster size > 10).

2.2 SES for the difference of seed-based functional connectivity between children with ADHD and TDC

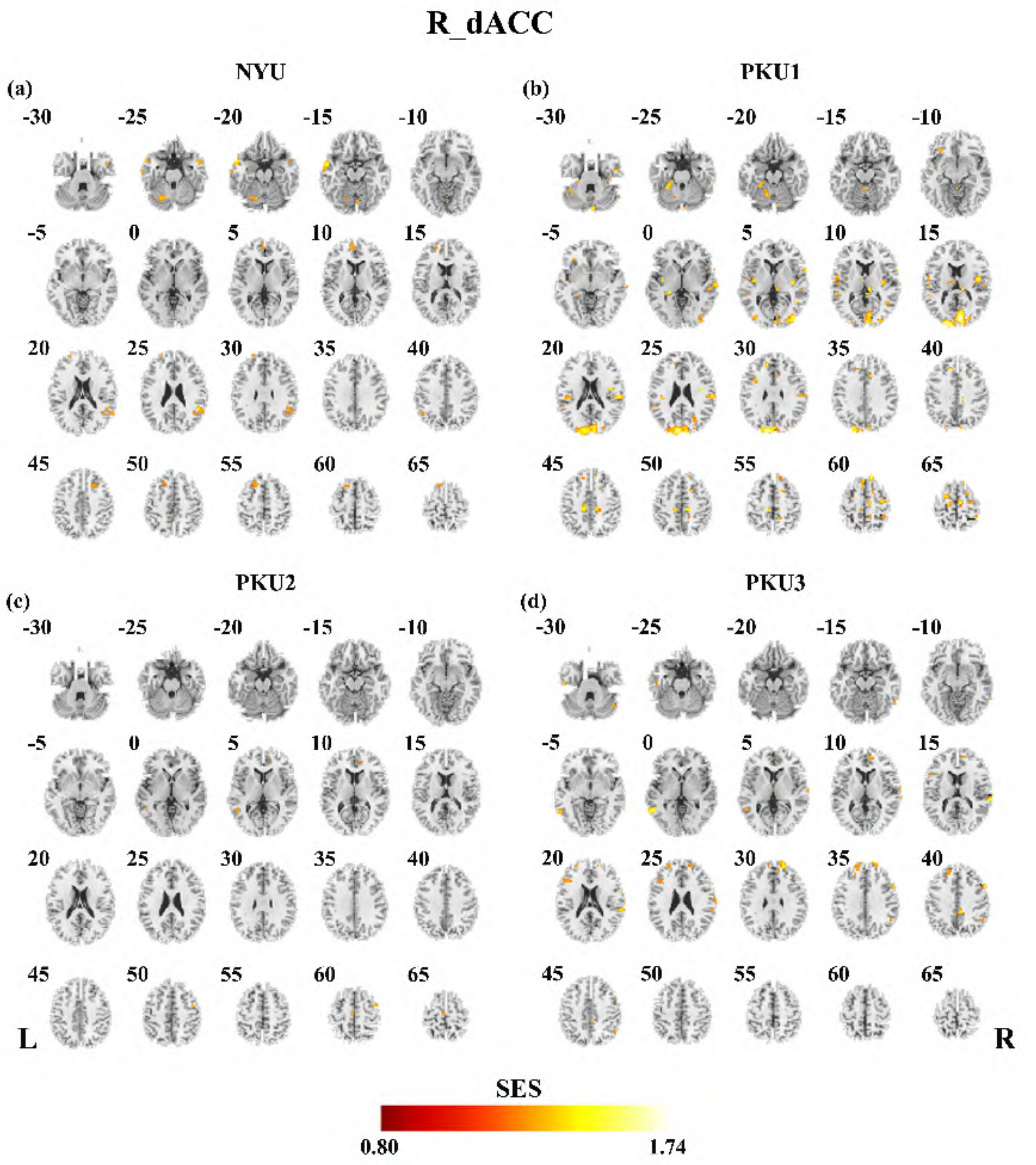

Figure S7. The SES for the difference of seed-based functional connectivity (using R_dACC as the seed region) between children with ADHD and TDC. a-d indicate the regions in which show SES > 0.80 for NYU, PKU1, PKU2 and PKU3, respectively (cluster size > 10).

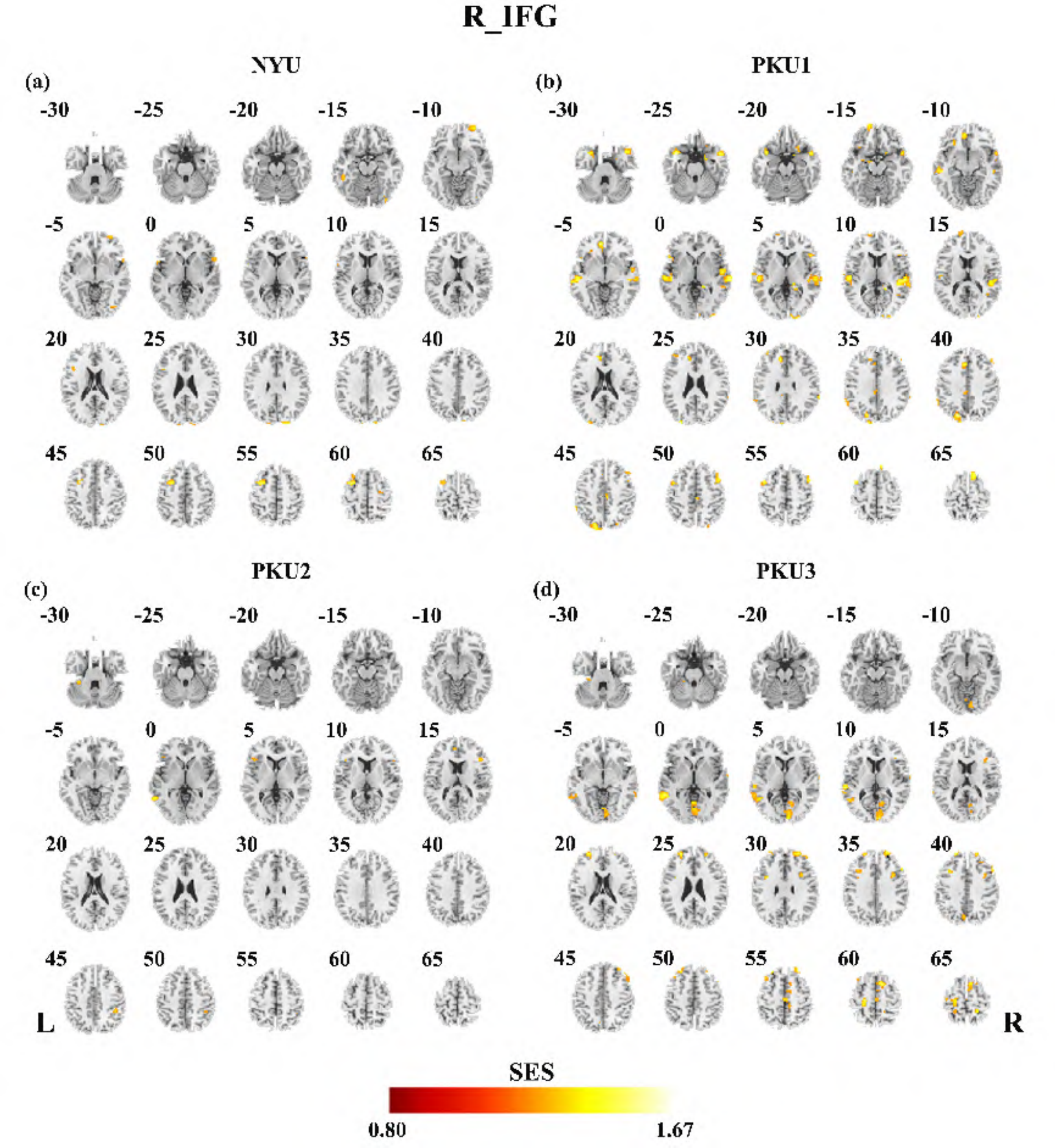

Figure S8. The SES for the difference of seed-based functional connectivity (using R_IFG as the seed region) between children with ADHD and TDC. a-d indicate the regions in which show SES > 0.80 for NYU, PKU1, PKU2 and PKU3, respectively (cluster size > 10).

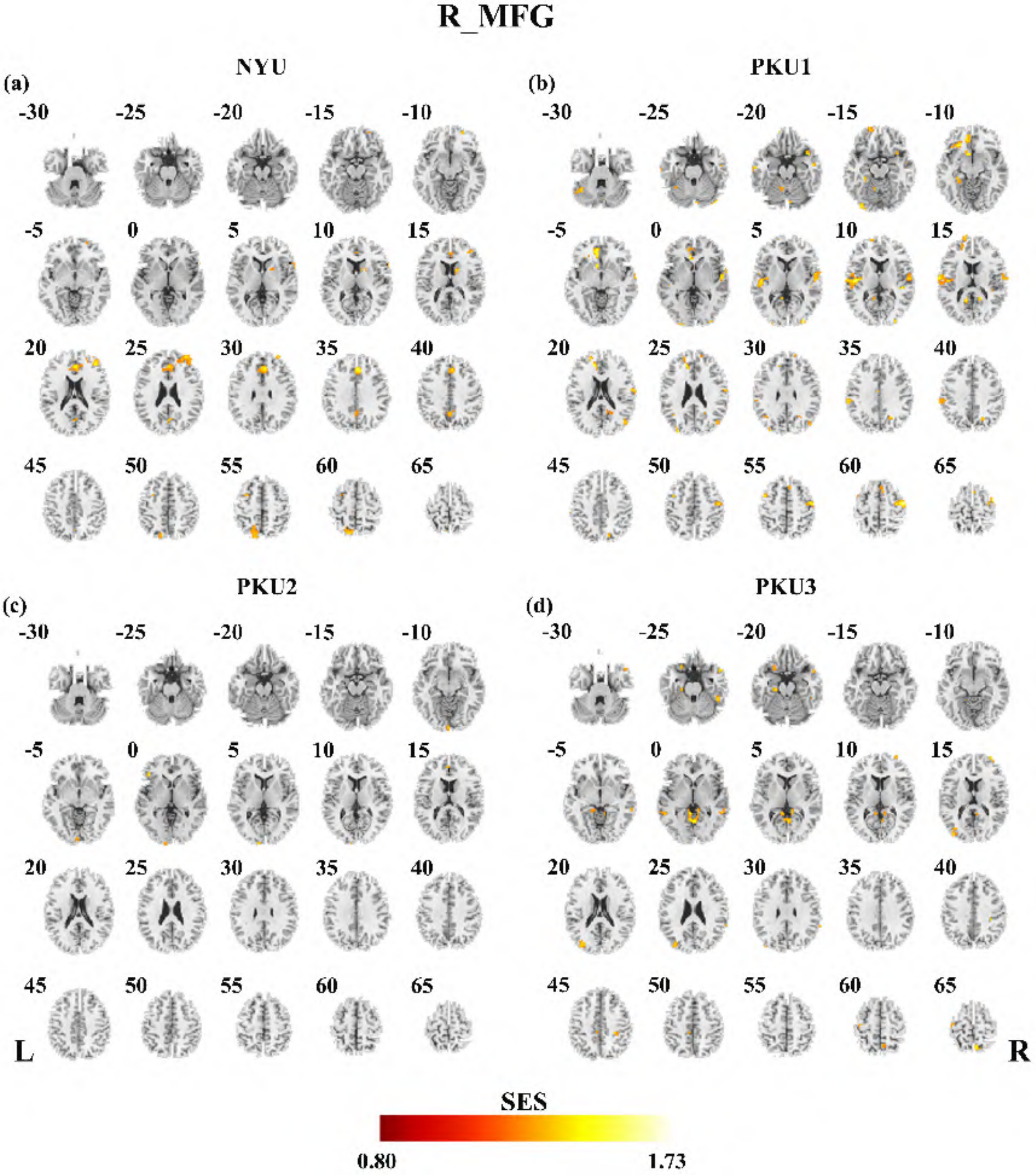

Figure S9. The SES for the difference of seed-based functional connectivity (using R_MFG as the seed region) between children with ADHD and TDC. a-d indicate the regions in which show SES > 0.80 for NYU, PKU1, PKU2 and PKU3, respectively (cluster size > 10).

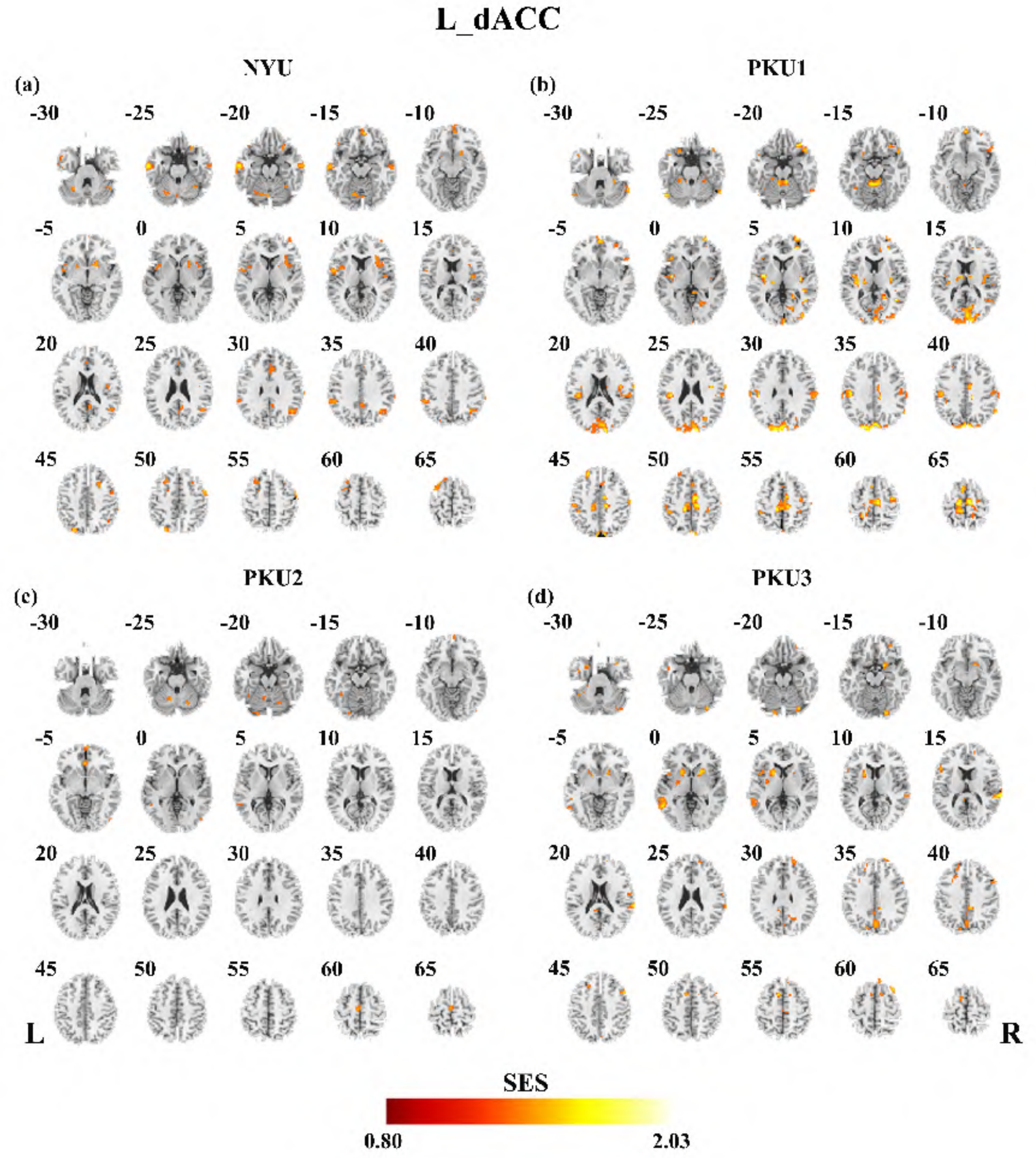

Figure S10. The SES for the difference of seed-based functional connectivity (using L_dACC as the seed region) between children with ADHD and TDC. a-d indicate the regions in which show SES > 0.80 for NYU, PKU1, PKU2 and PKU3, respectively (cluster size > 10).

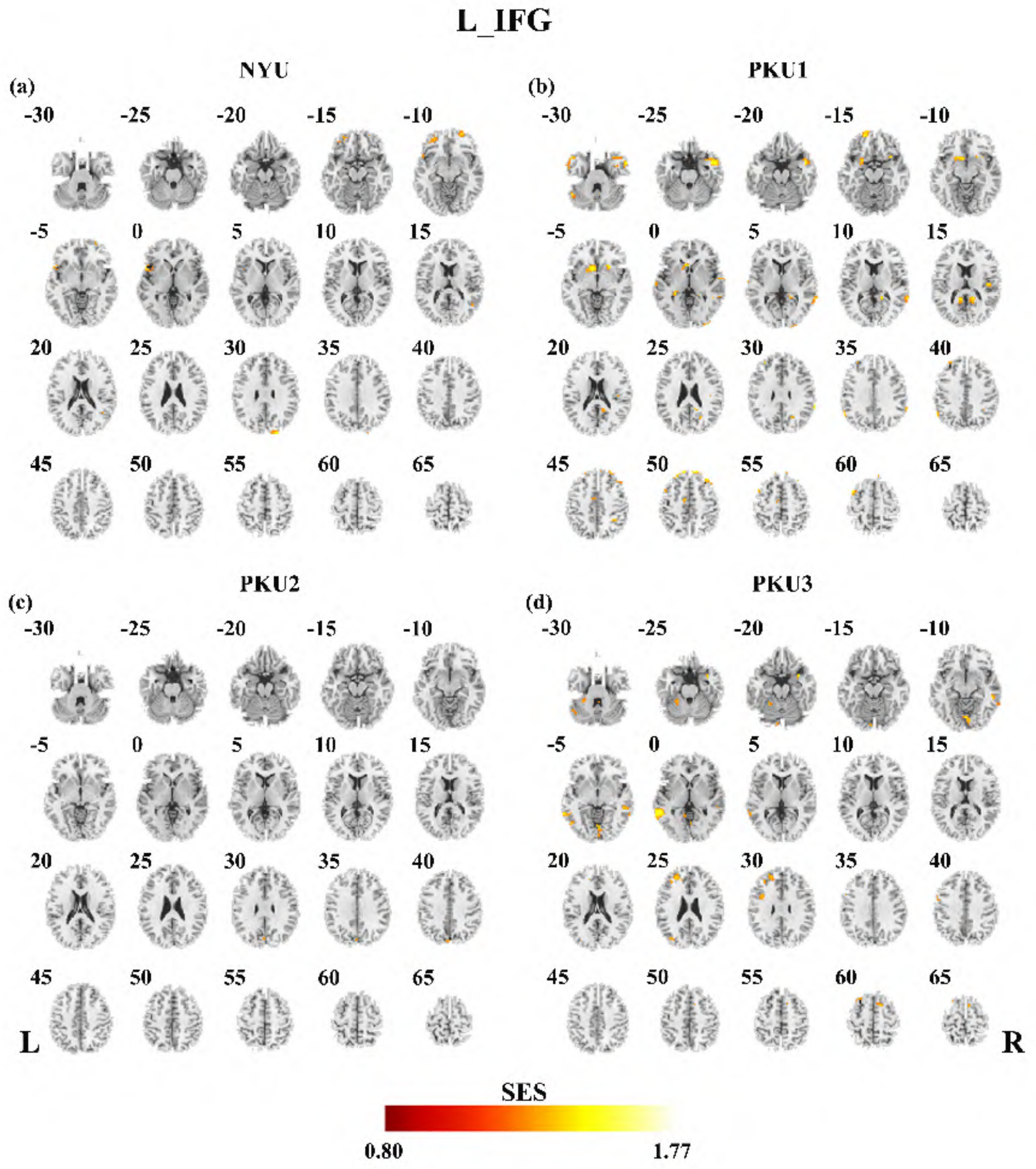

Figure S11. The SES for the difference of seed-based functional connectivity (using L_IFG as the seed region) between children with ADHD and TDC. a-d indicate the regions in which show SES > 0.80 for NYU, PKU1, PKU2 and PKU3, respectively (cluster size > 10).

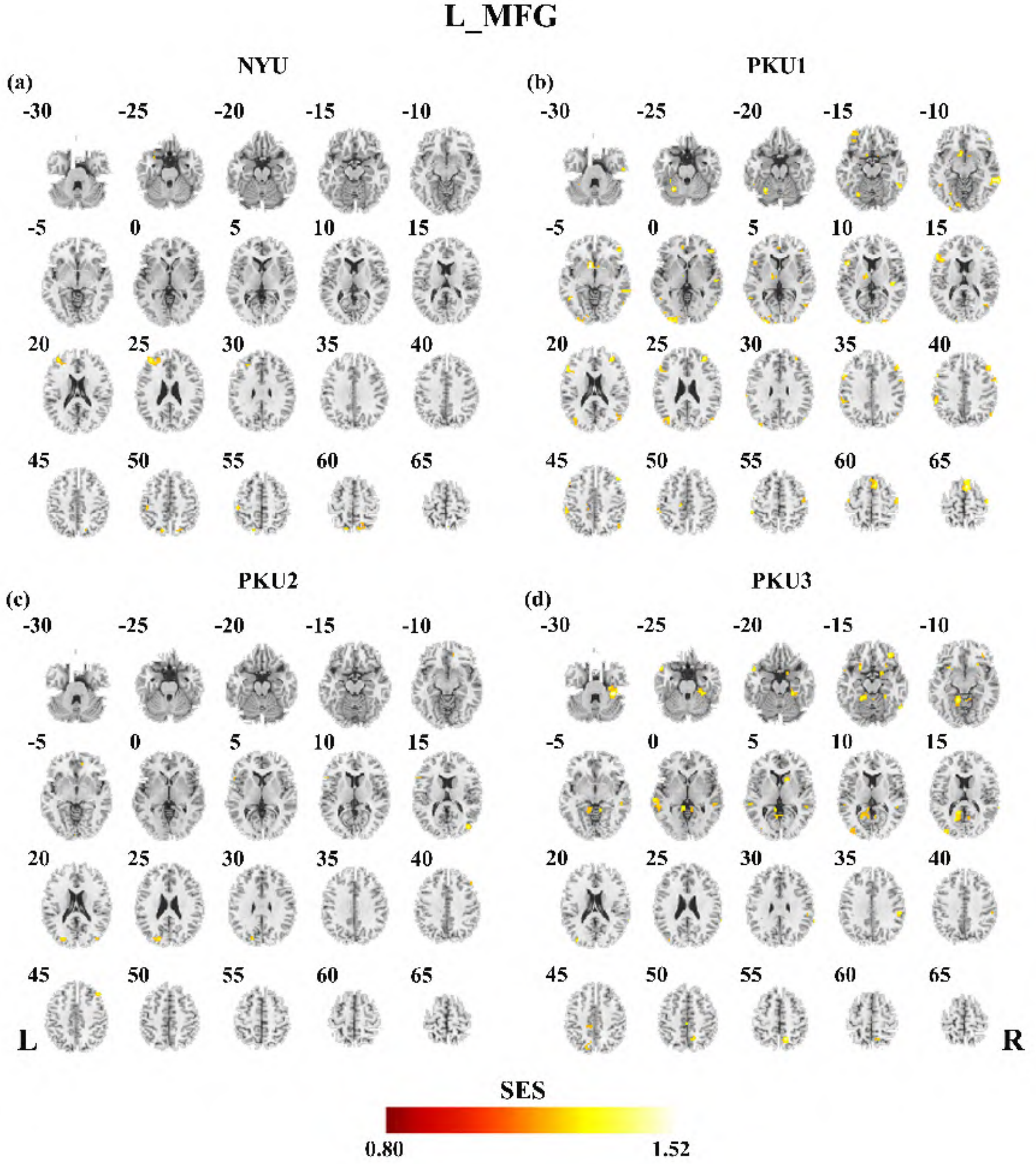

Figure S12. The SES for the difference of seed-based functional connectivity (using L_MFG as the seed region) between children with ADHD and TDC. a-d indicate the regions in which show SES > 0.80 for NYU, PKU1, PKU2 and PKU3, respectively (cluster size > 10).

2.3 The difference of seed-based functional connectivity between children with ADHD subtypes and TDC

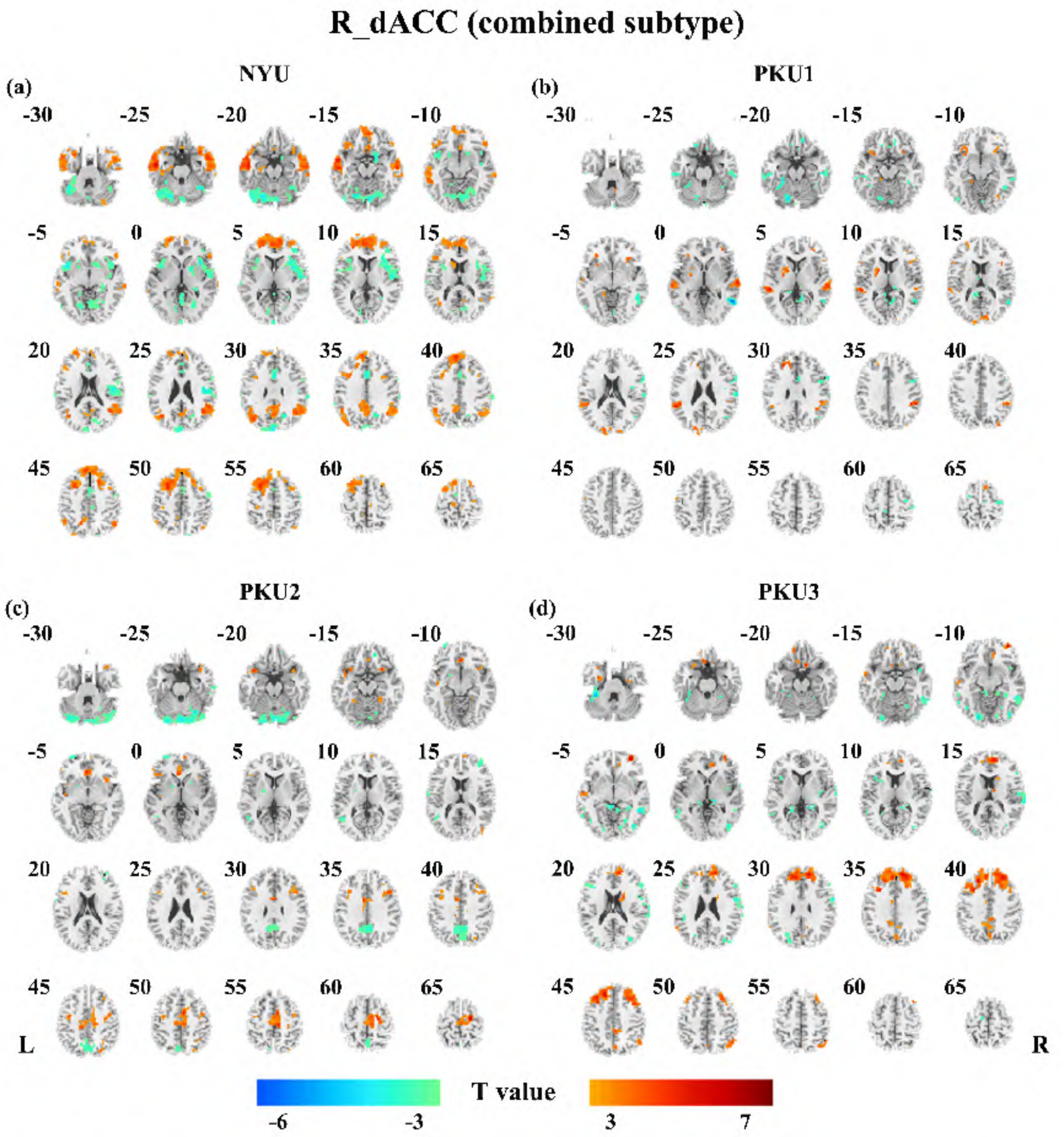

Figure S13. Clusters showing significant functional connectivity differences between TDC and children with ADHD combined subtype. a-d indicate the result of NYU, PKU1, PKU2 and PKU3, respectively. Cold color indicates the region in which combined subtype subjects had decreased functional connectivity with R_dACC compared with TDC and the warm color indicates the opposite (p < 0.05, cluster size > 10).

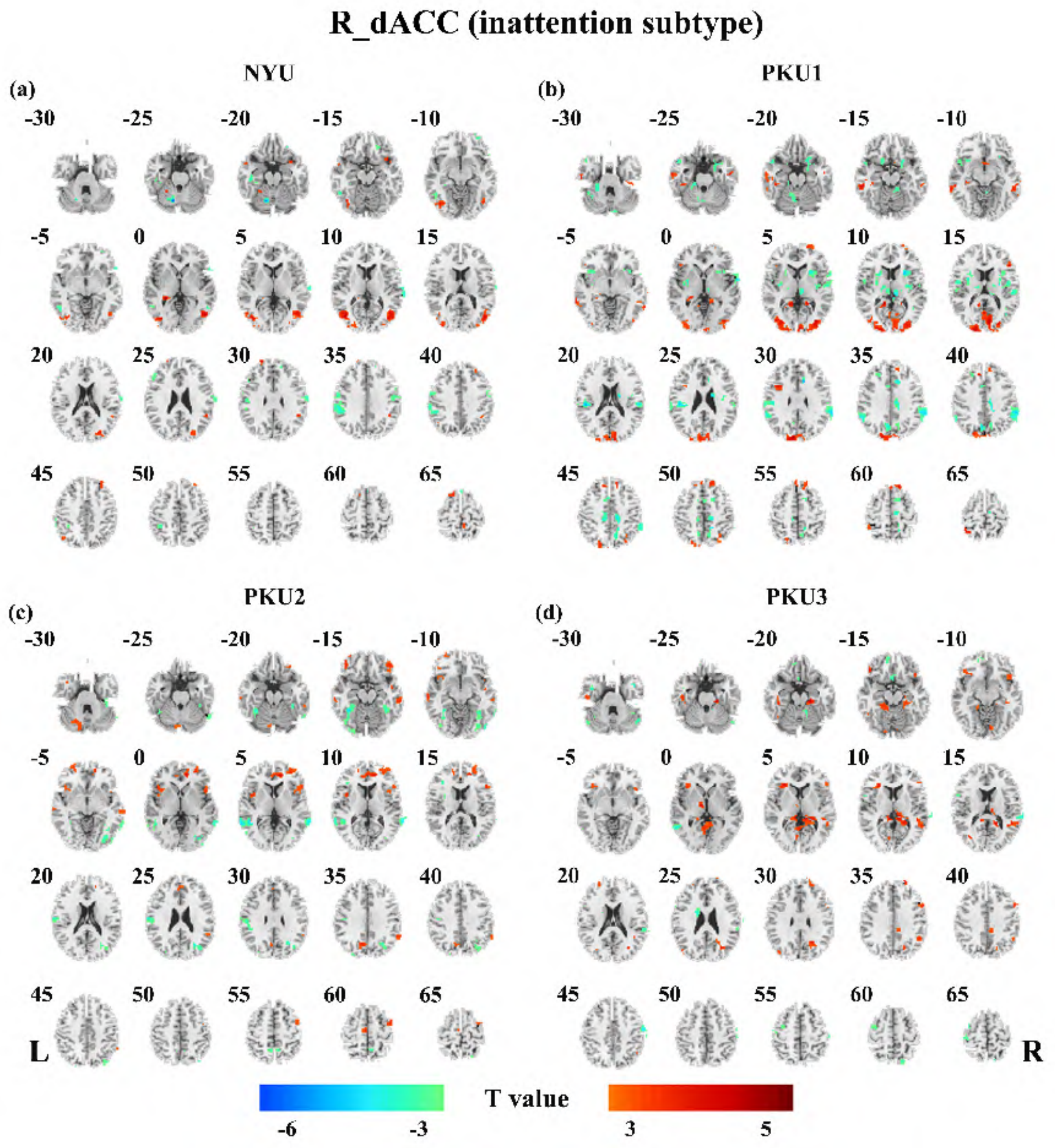

Figure S14. Clusters showing significant functional connectivity differences between TDC and children with ADHD inattention subtype. a-d indicate the result of NYU, PKU1, PKU2 and PKU3, respectively. Cold color indicates the region in which inattention subtype subjects had decreased functional connectivity with R_dACC compared with TDC and the warm color indicates the opposite (p < 0.05, cluster size > 10).

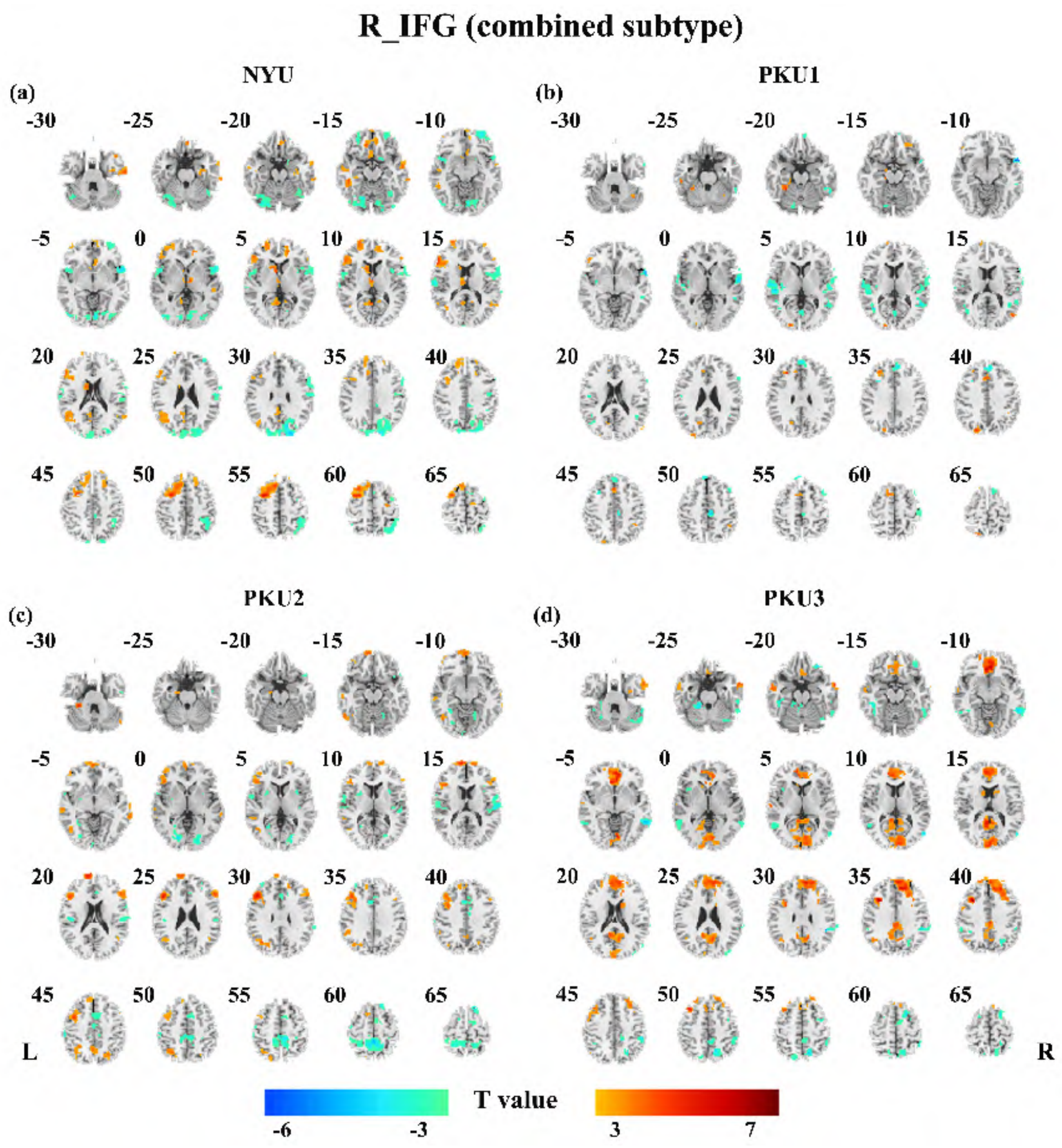

Figure S15. Clusters showing significant functional connectivity differences between TDC and children with ADHD combined subtype. a-d indicate the result of NYU, PKU1, PKU2 and PKU3, respectively. Cold color indicates the region in which combined subtype subjects had decreased functional connectivity with R_IFG compared with TDC and the warm color indicates the opposite (p < 0.05, cluster size > 10).

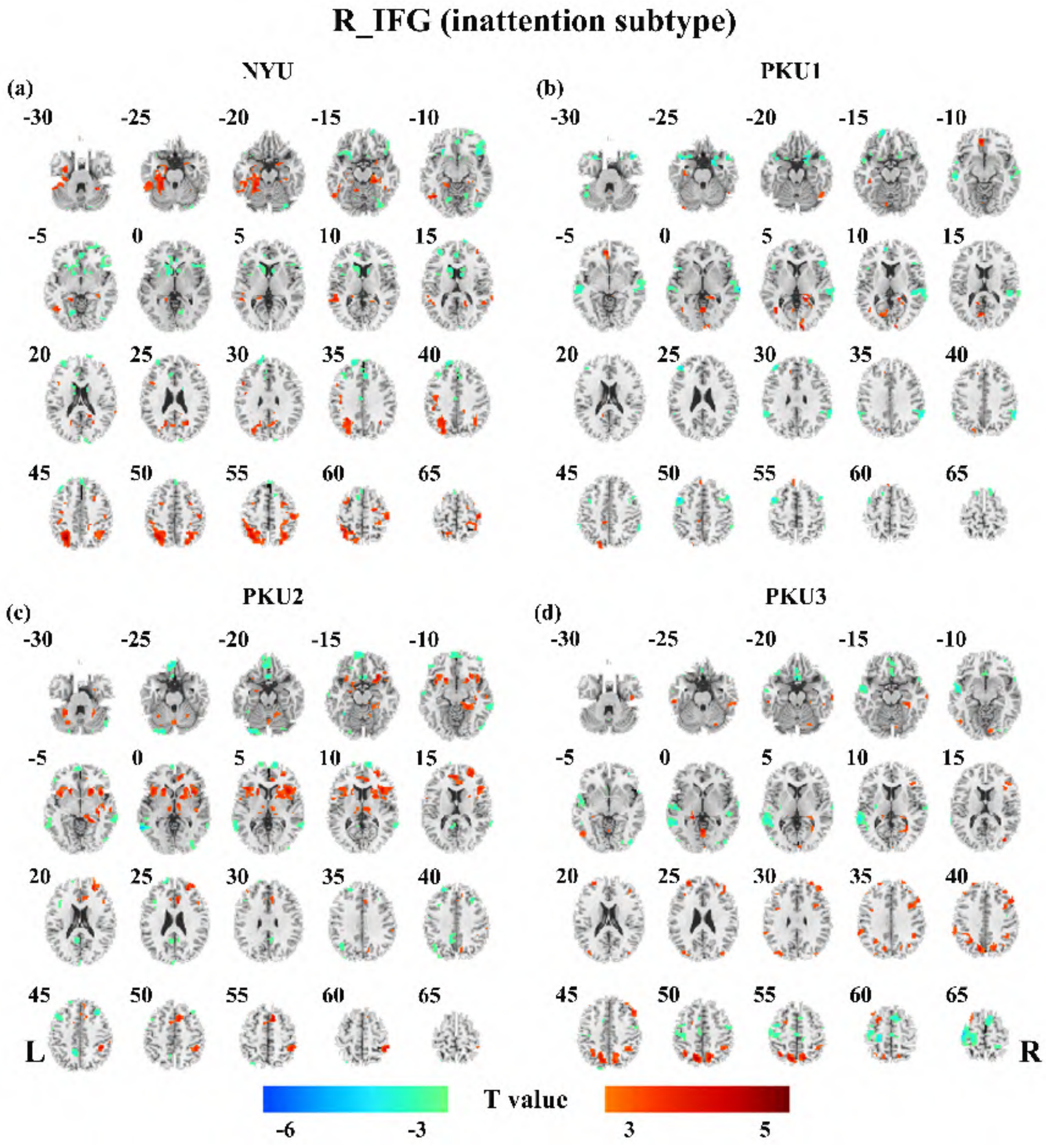

Figure S16. Clusters showing significant functional connectivity differences between TDC and children with ADHD inattention subtype. a-d indicate the result of NYU, PKU1, PKU2 and PKU3, respectively. Cold color indicates the region in which inattention subtype subjects had decreased functional connectivity with R_IFG compared with TDC and the warm color indicates the opposite ($p < 0.05$, cluster size > 10).

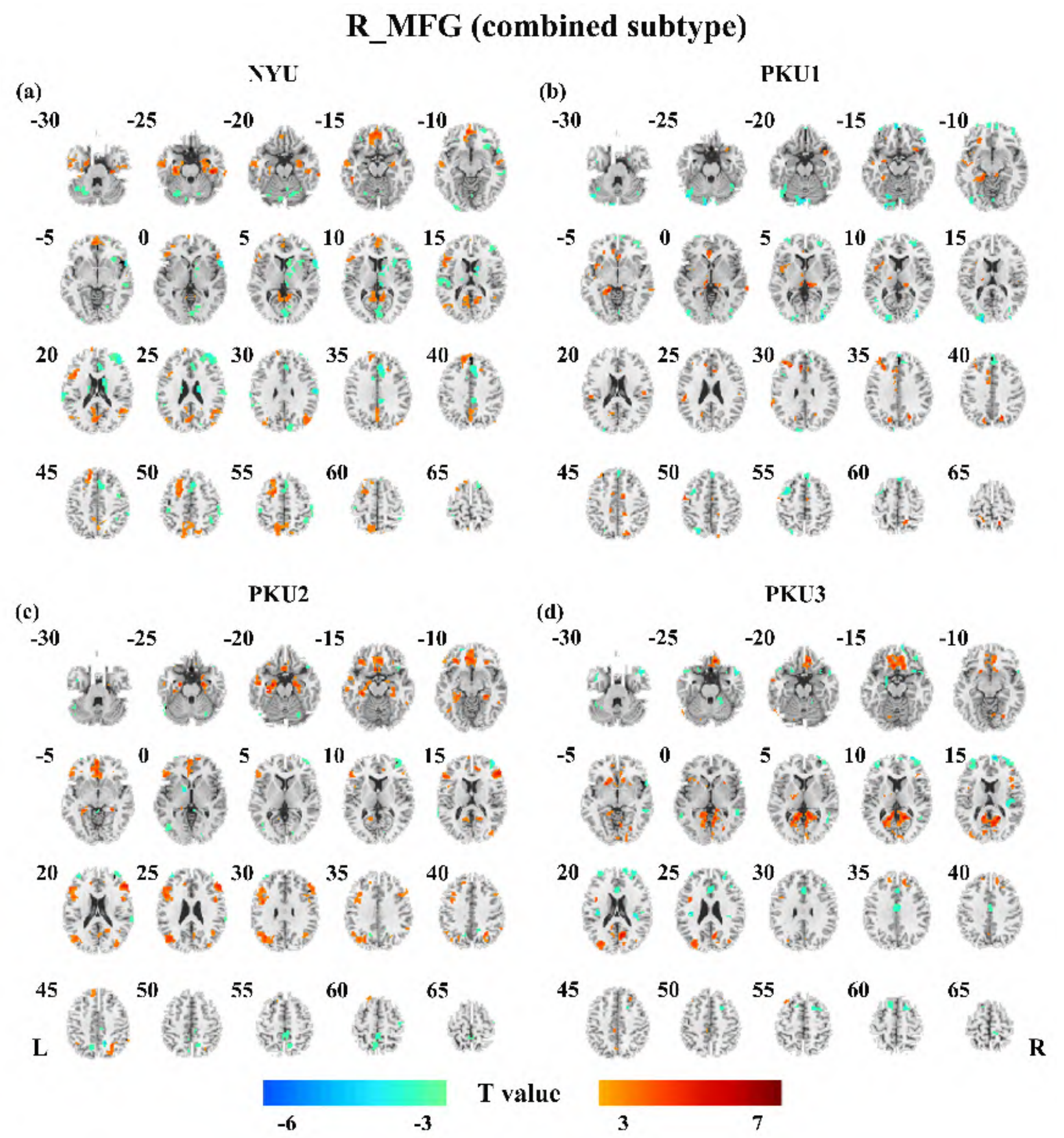

Figure S17. Clusters showing significant functional connectivity differences between TDC and children with ADHD combined subtype. a-d indicate the result of NYU, PKU1, PKU2 and PKU3, respectively. Cold color indicates the region in which combined subtype subjects had decreased functional connectivity with R_MFG compared with TDC and the warm color indicates the opposite ($p < 0.05$, cluster size > 10).

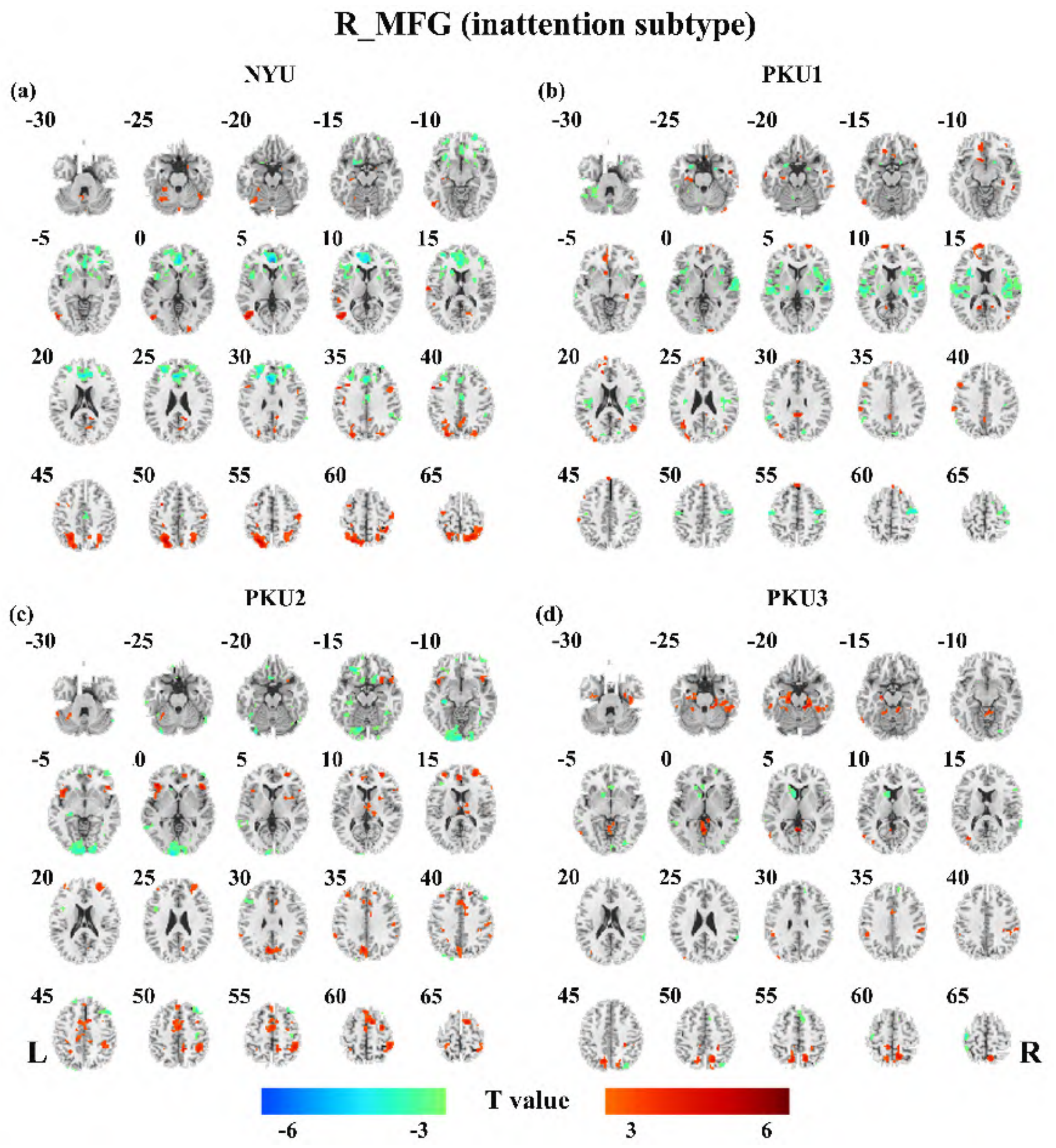

Figure S18. Clusters showing significant functional connectivity differences between TDC and children with ADHD inattention subtype. a-d indicate the result of NYU, PKU1, PKU2 and PKU3, respectively. Cold color indicates the region in which inattention subtype subjects had decreased functional connectivity with R_MFG compared with TDC and the warm color indicates the opposite (p < 0.05, cluster size > 10).

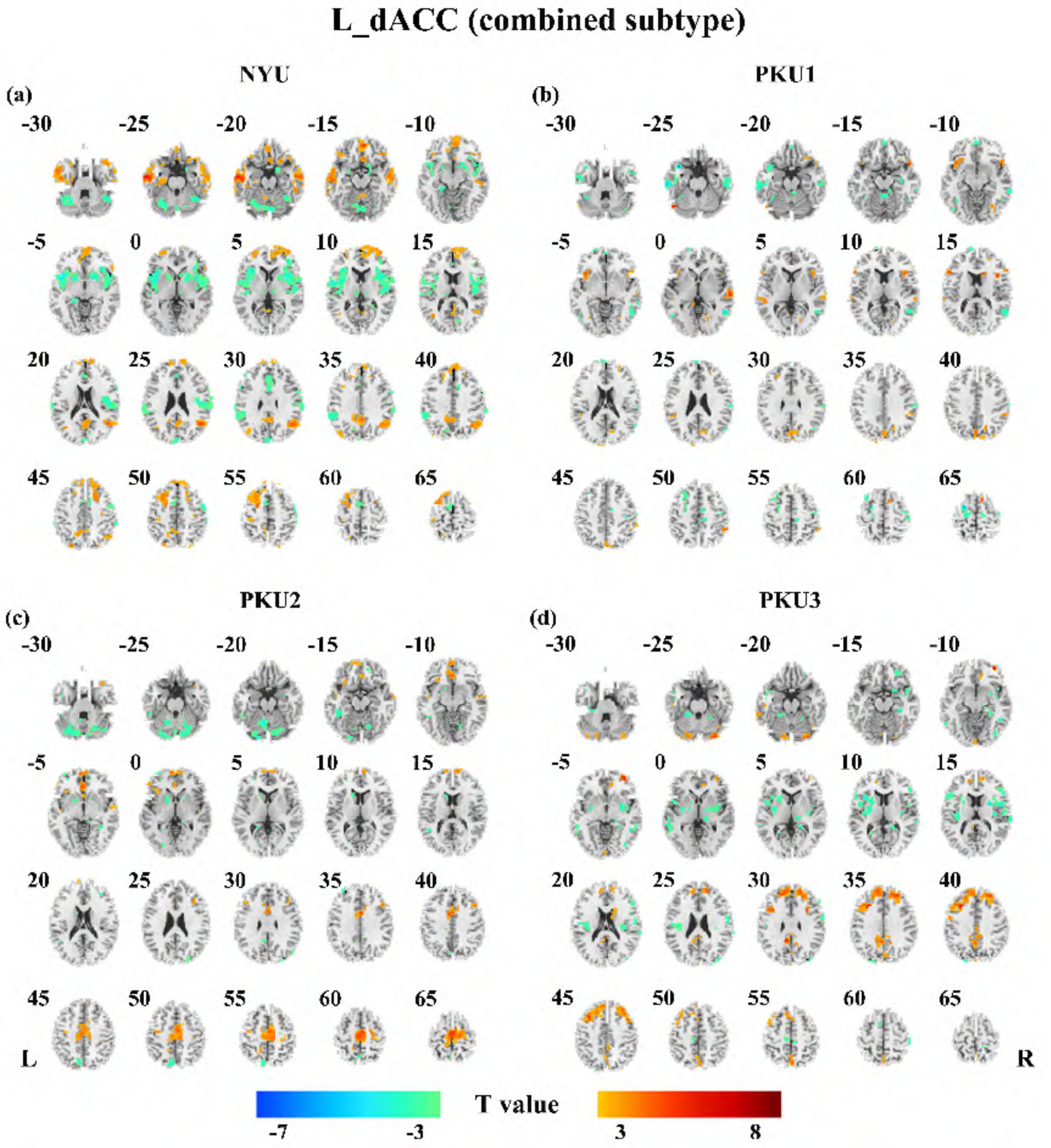

Figure S19. Clusters showing significant functional connectivity differences between TDC and children with ADHD combined subtype. a-d indicate the result of NYU, PKU1, PKU2 and PKU3, respectively. Cold color indicates the region in which combined subtype subjects had decreased functional connectivity with L_dACC compared with TDC and the warm color indicates the opposite ($p < 0.05$, cluster size > 10).

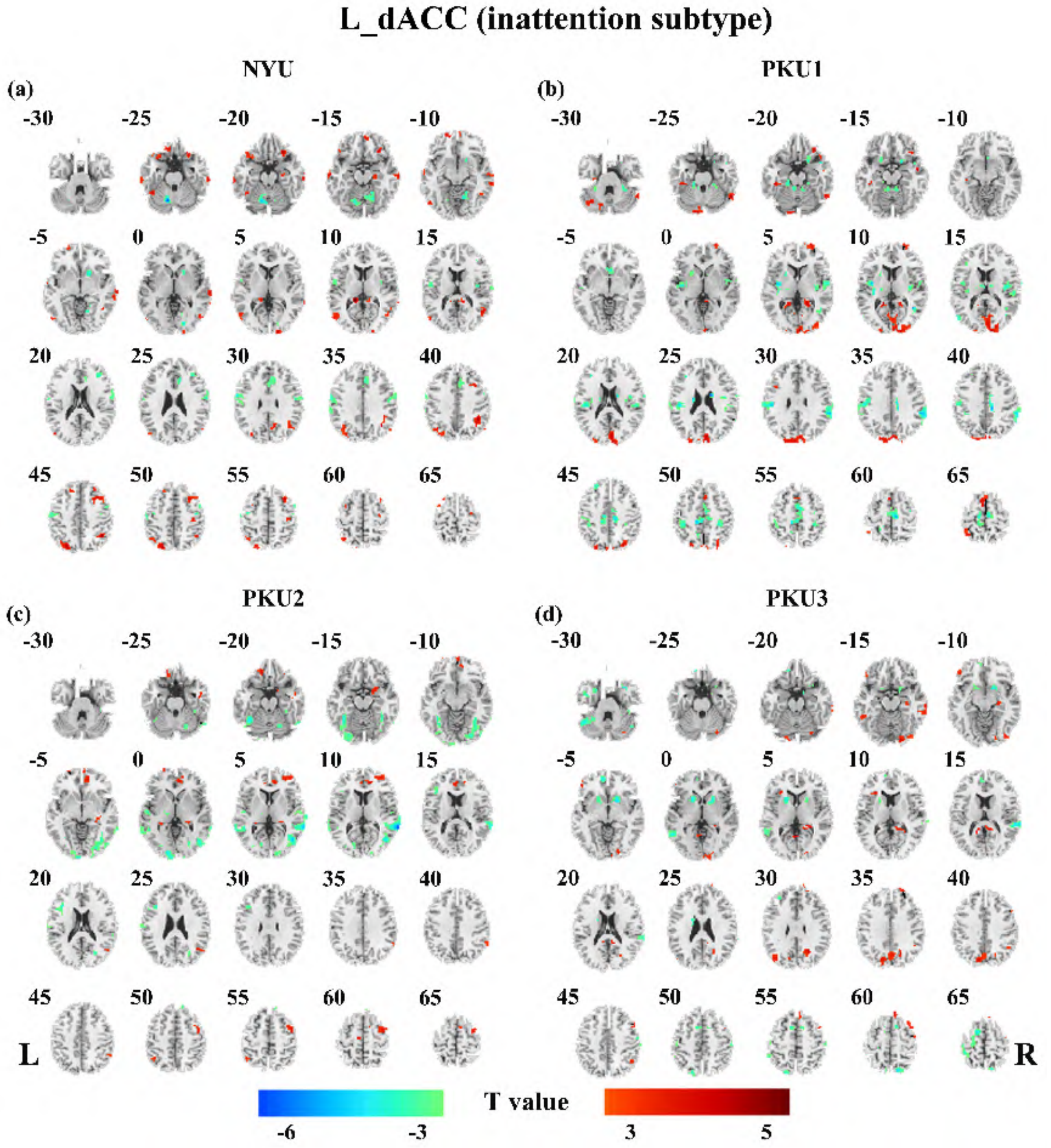

Figure S20. Clusters showing significant functional connectivity differences between TDC and children with ADHD inattention subtype. a-d indicate the result of NYU, PKU1, PKU2 and PKU3, respectively. Cold color indicates the region in which inattention subtype subjects had decreased functional connectivity with L_dACC compared with TDC and the warm color indicates the opposite ($p < 0.05$, cluster size > 10).

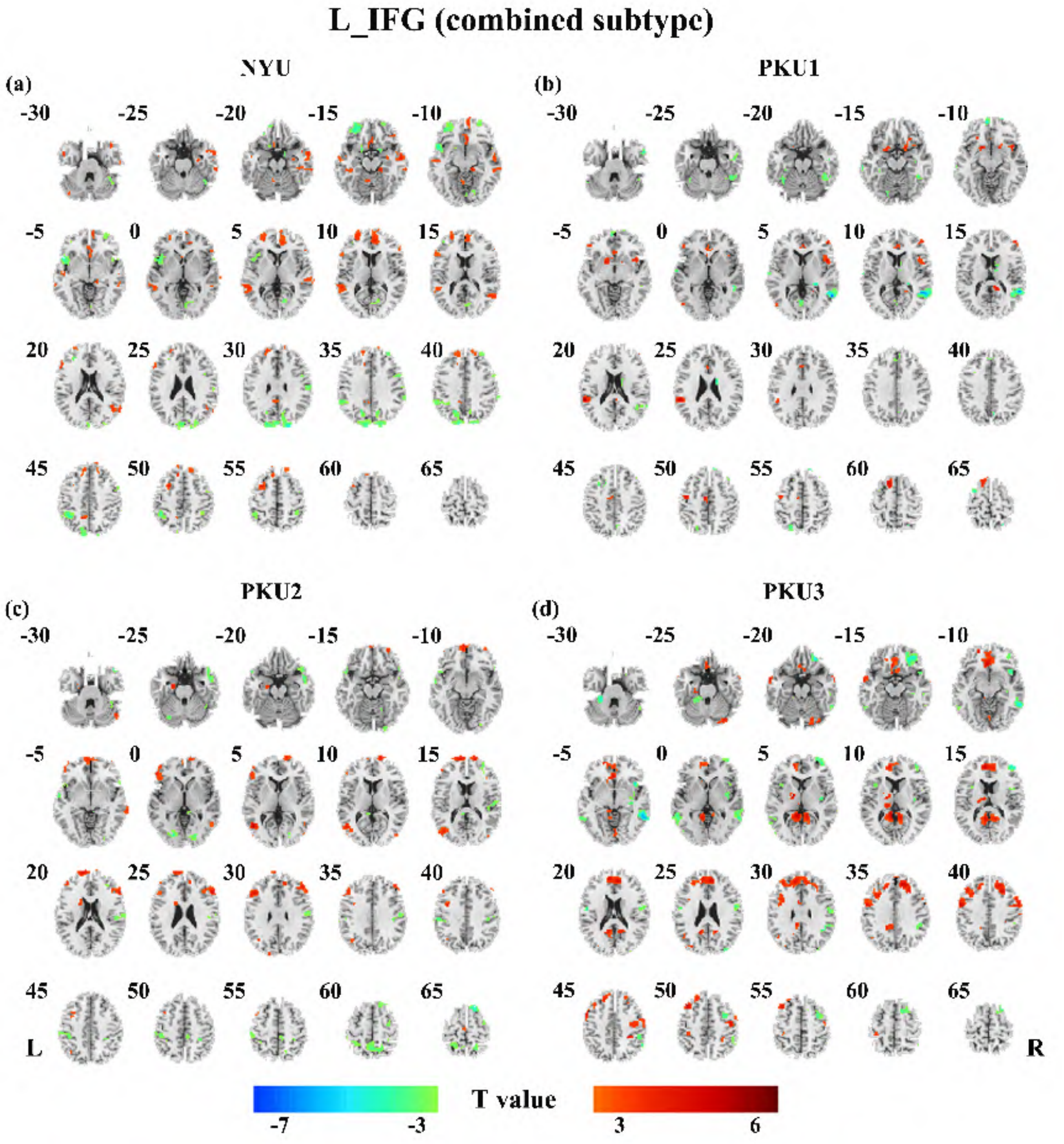

Figure S21. Clusters showing significant functional connectivity differences between TDC and children with ADHD combined subtype. a-d indicate the result of NYU, PKU1, PKU2 and PKU3, respectively. Cold color indicates the region in which combined subtype subjects had decreased functional connectivity with L_IFG compared with TDC and the warm color indicates the opposite (p < 0.05, cluster size > 10).

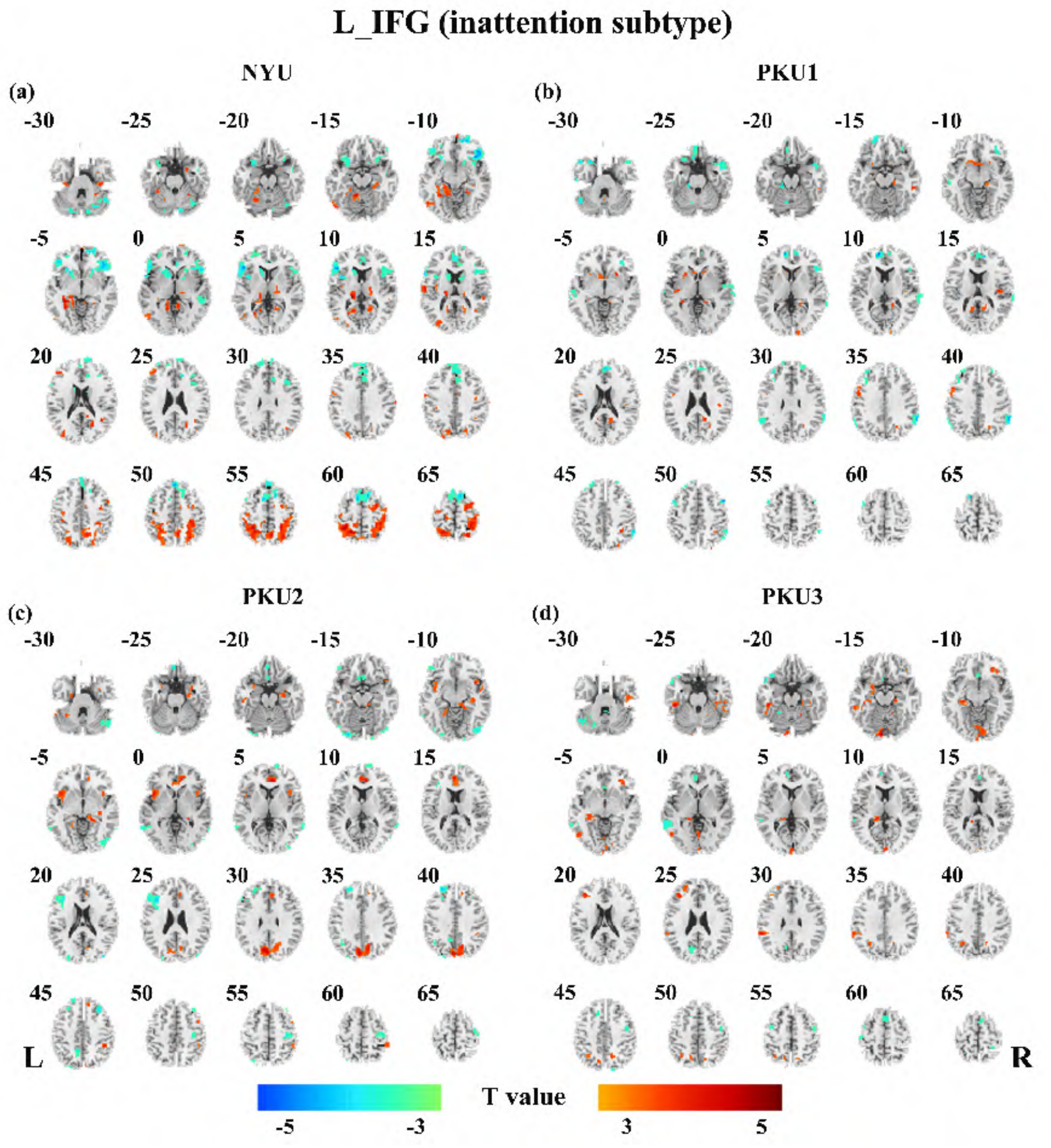

Figure S22. Clusters showing significant functional connectivity differences between TDC and children with ADHD inattention subtype. a-d indicate the result of NYU, PKU1, PKU2 and PKU3, respectively. Cold color indicates the region in which inattention subtype subjects had decreased functional connectivity with L_IFG compared with TDC and the warm color indicates the opposite ($p < 0.05$, cluster size > 10).

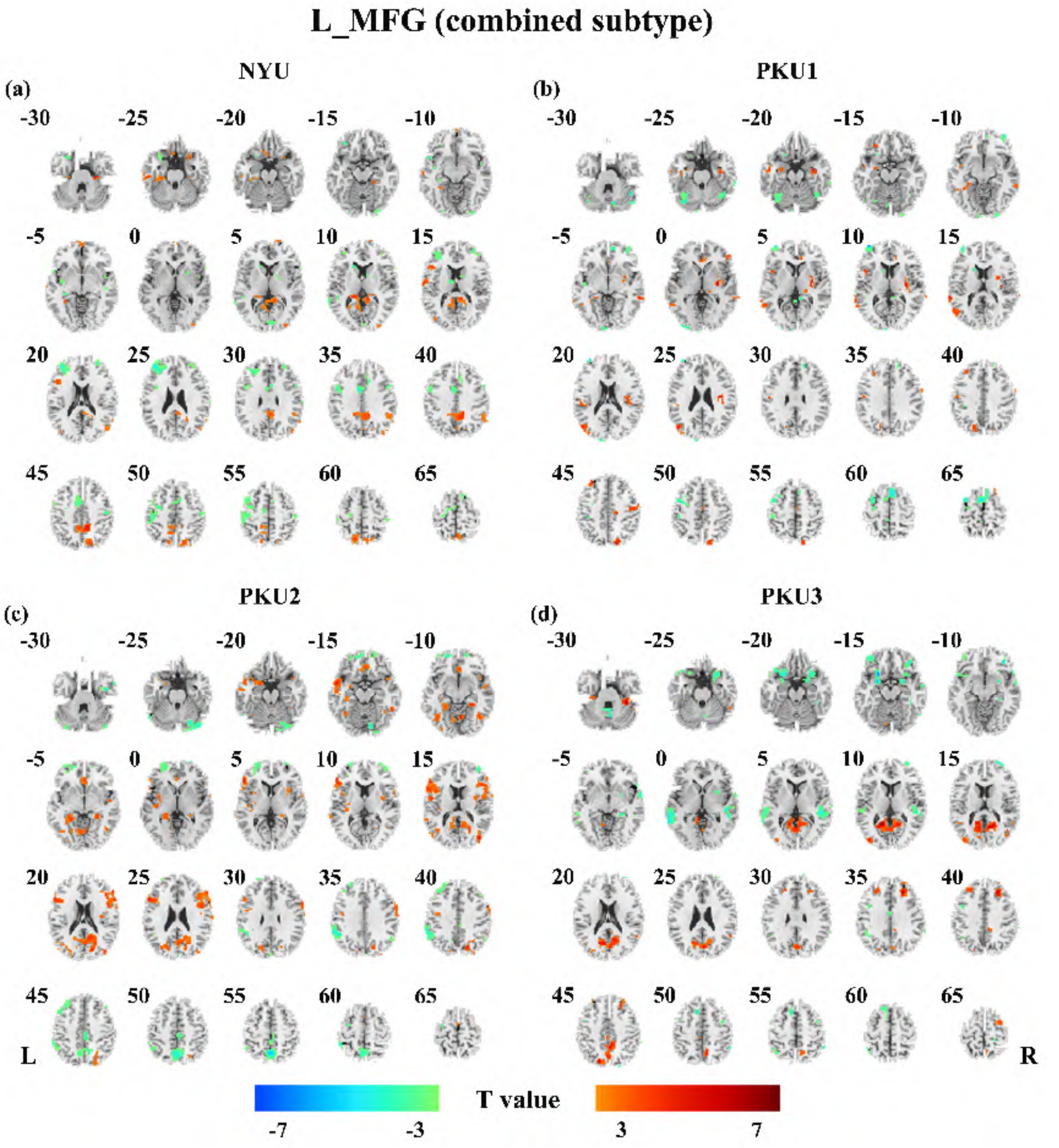

Figure S23. Clusters showing significant functional connectivity differences between TDC and children with ADHD combined subtype. a-d indicate the result of NYU, PKU1, PKU2 and PKU3, respectively. Cold color indicates the region in which combined subtype subjects had decreased functional connectivity with L_MFG compared with TDC and the warm color indicates the opposite ($p < 0.05$, cluster size > 10).

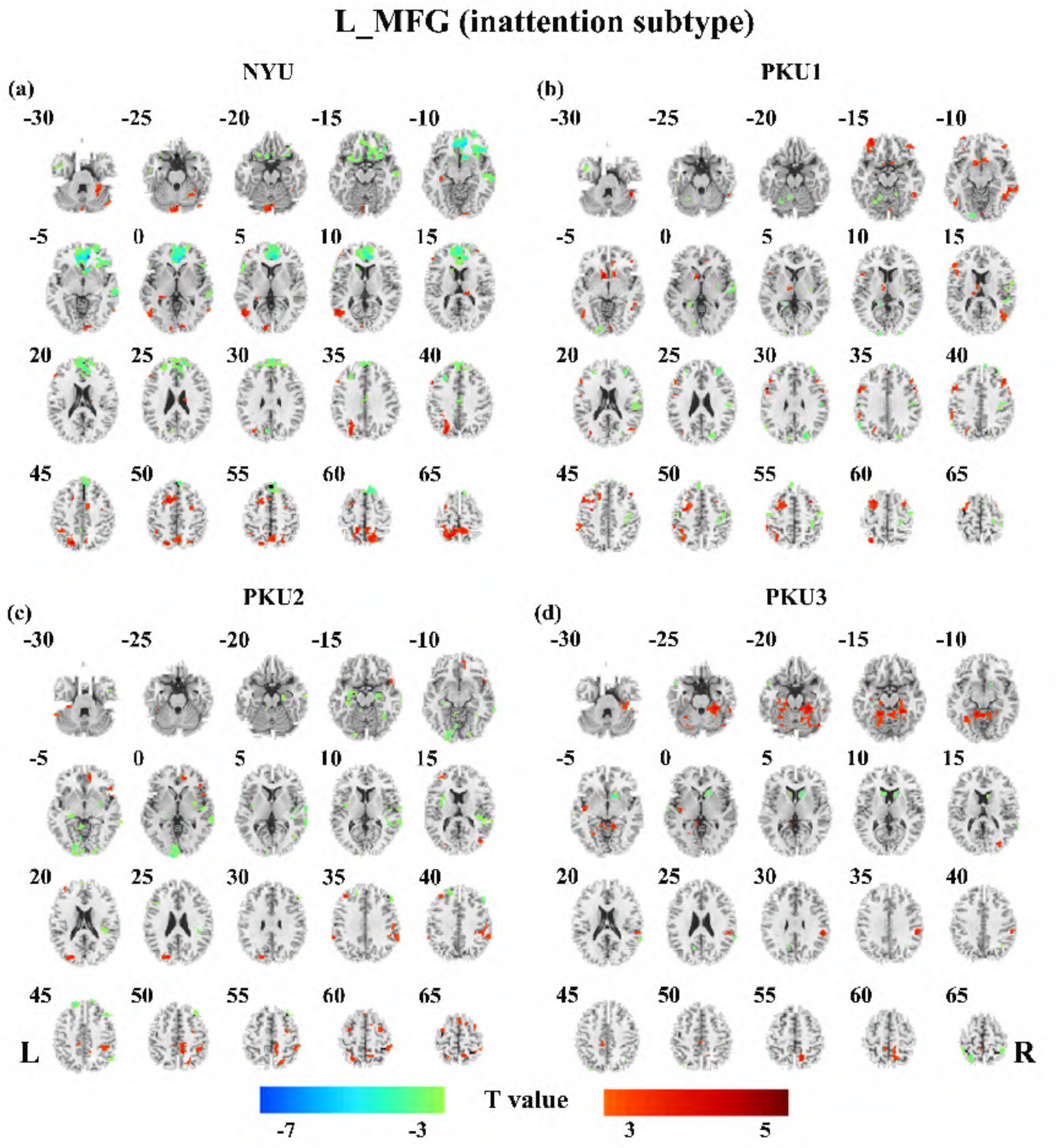

Figure S24. Clusters showing significant functional connectivity differences between TDC and children with ADHD inattention subtype. a-d indicate the result of NYU, PKU1, PKU2 and PKU3, respectively. Cold color indicates the region in which inattention subtype subjects had decreased functional connectivity with L_MFG compared with TDC and the warm color indicates the opposite (p < 0.05, cluster size > 10).

2.4 SES for the difference of the seed-based functional connectivity between children with ADHD subtypes and TDC

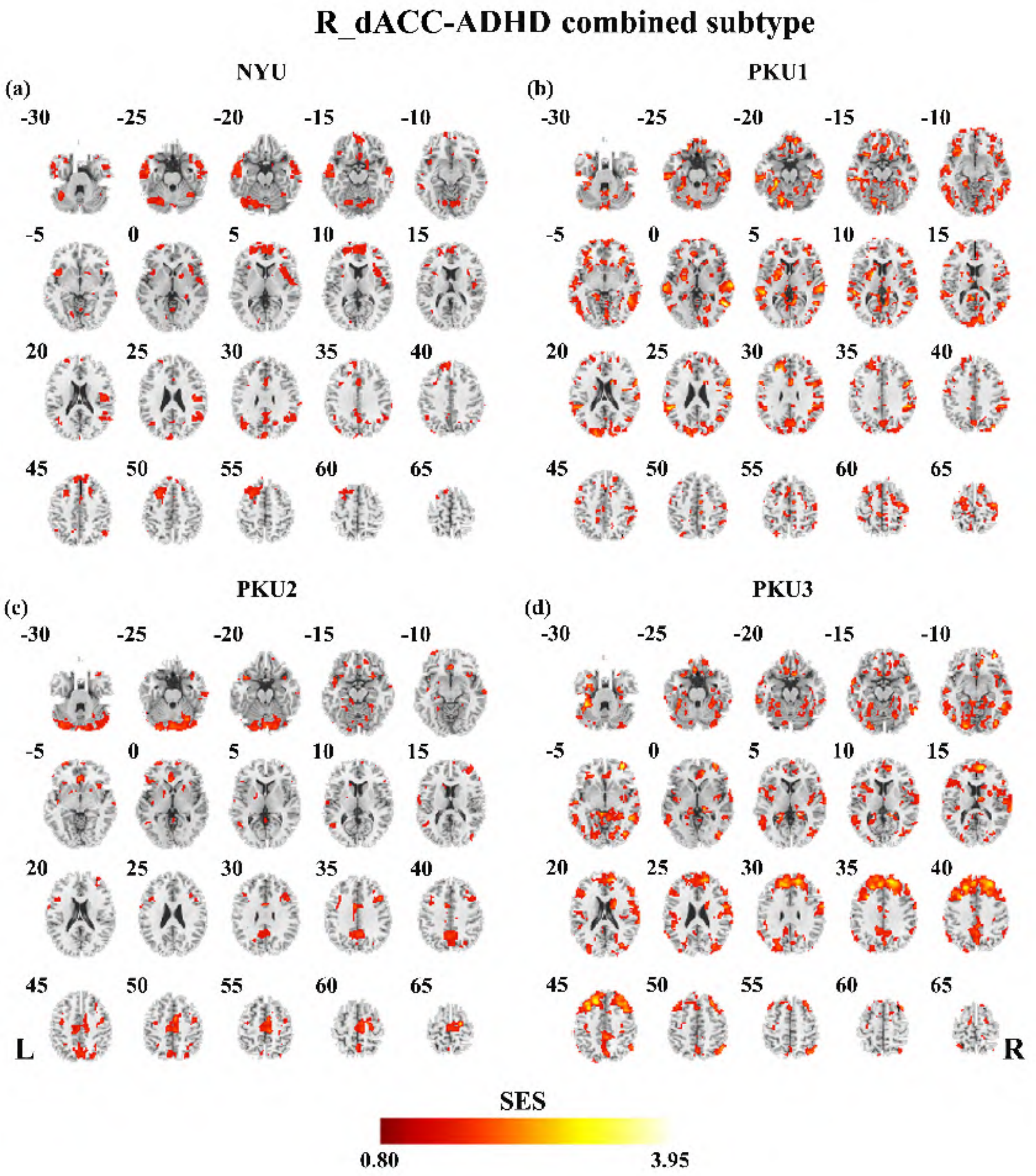

Figure S25. The SES for the difference of seed-based functional connectivity (using R_dACC as the seed region) between children with ADHD combined subtype and TDC. a-d indicate the regions in which show SES > 0.80 for NYU, PKU1, PKU2 and PKU3, respectively (cluster size > 10).

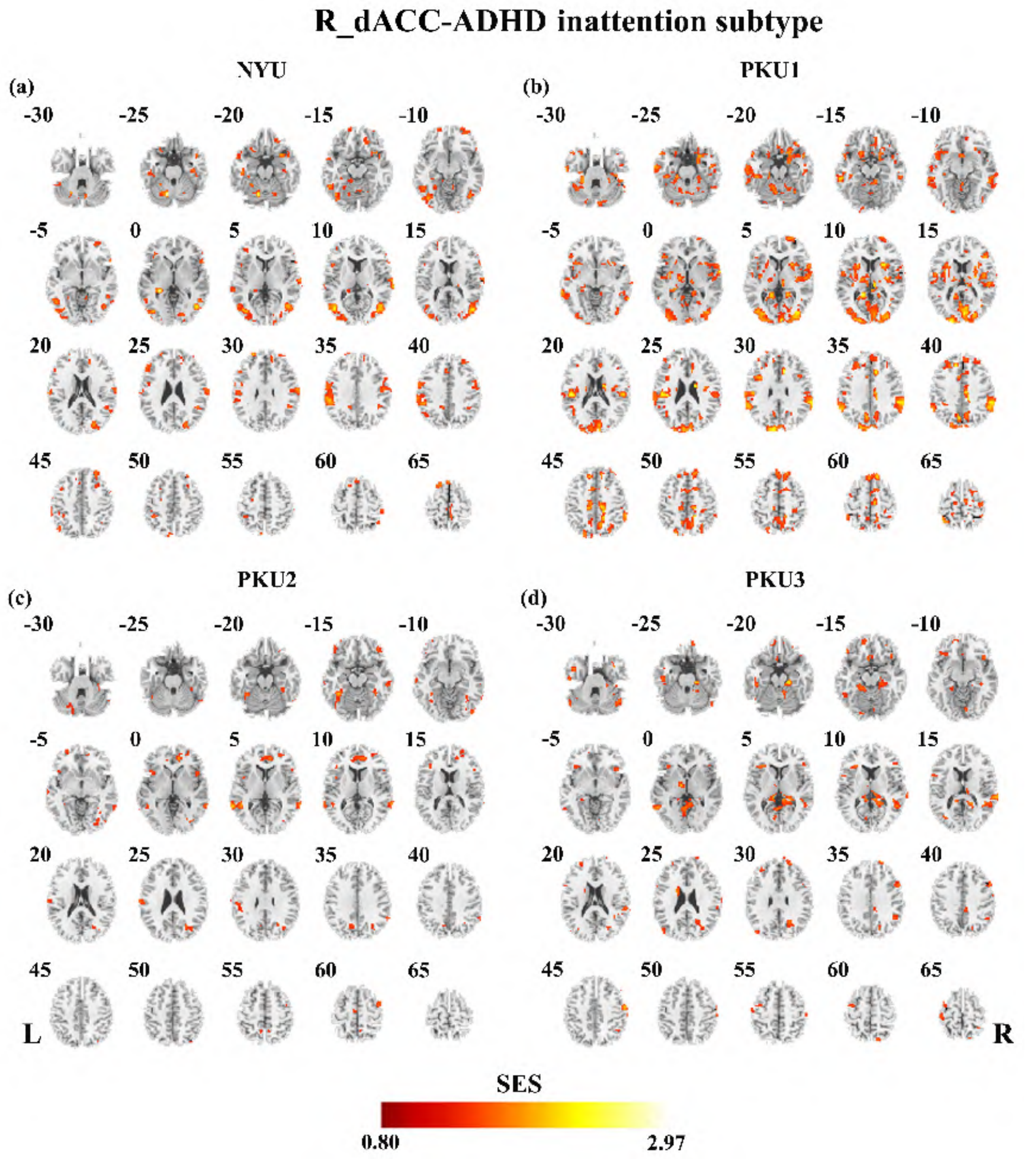

Figure S26. The SES for the difference of seed-based functional connectivity (using R_dACC as the seed region) between children with ADHD inattention subtype and TDC. a-d indicate the regions in which show SES > 0.80 for NYU, PKU1, PKU2 and PKU3, respectively (cluster size > 10).

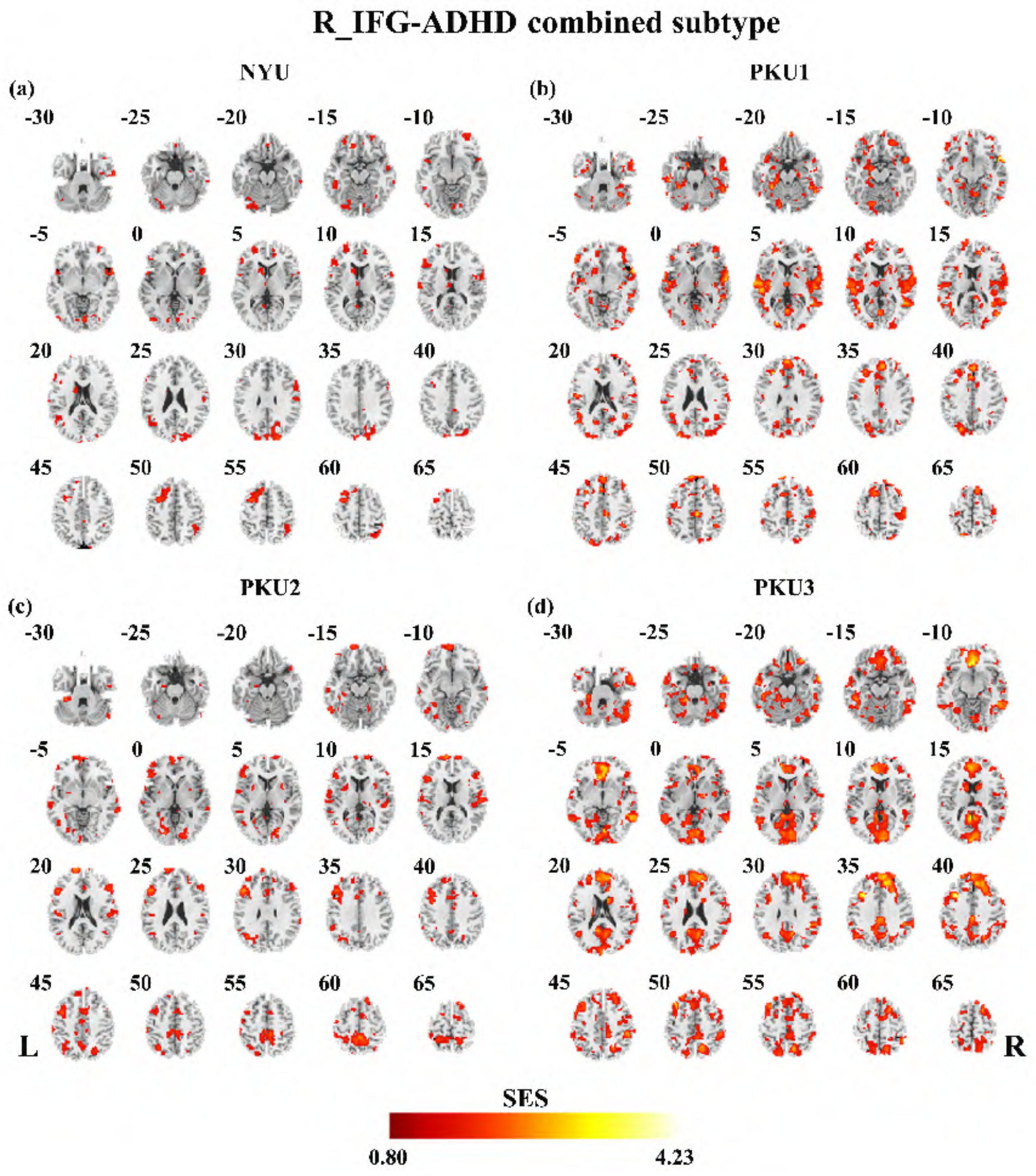

Figure S27. The SES for the difference of seed-based functional connectivity (using R_IFG as the seed region) between children with ADHD combined subtype and TDC. a-d indicate the regions in which show SES > 0.80 for NYU, PKU1, PKU2 and PKU3, respectively (cluster size > 10).

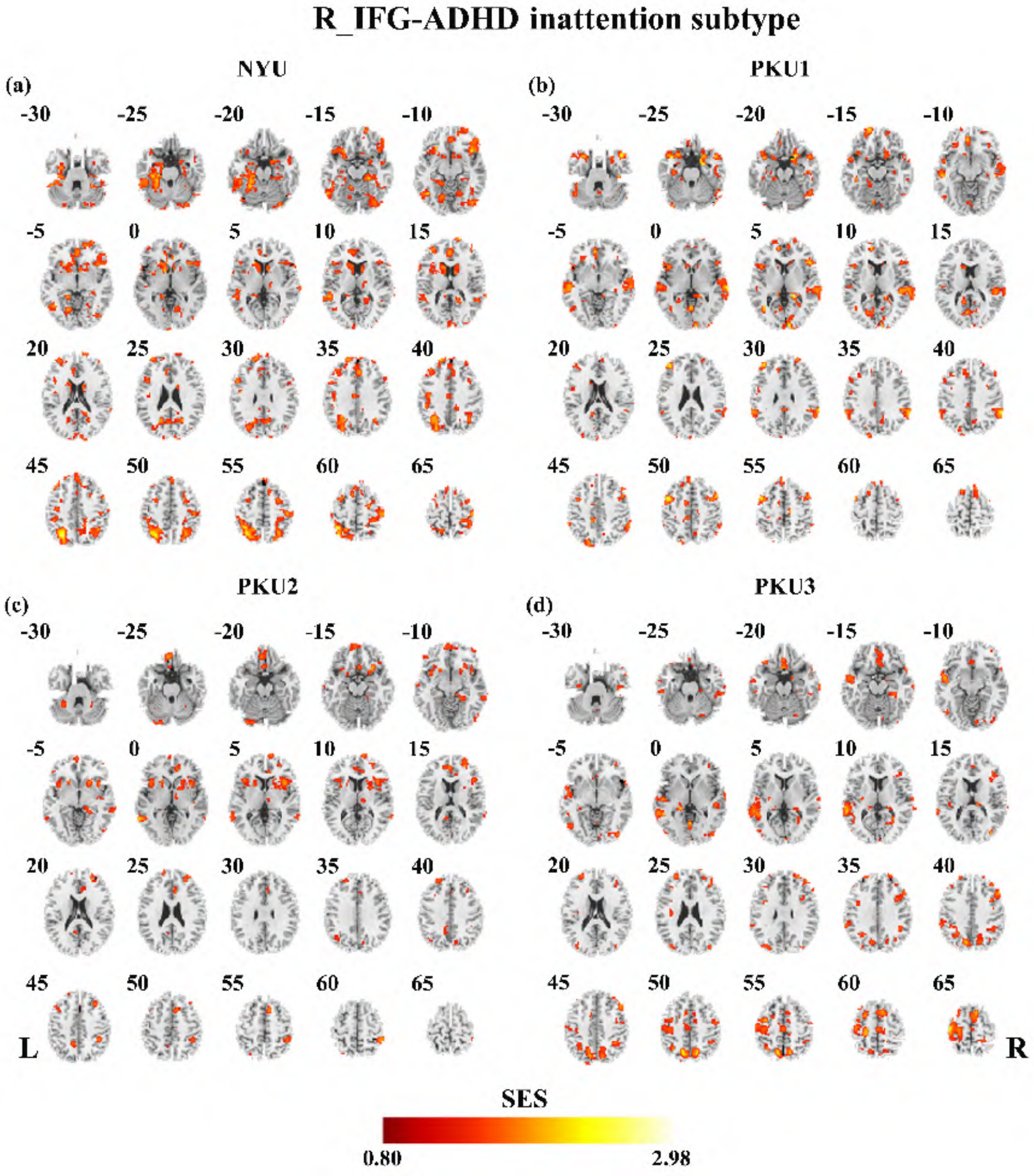

Figure S28. The SES for the difference of seed-based functional connectivity (using R_IFG as the seed region) between children with ADHD inattention subtype and TDC. a-d indicate the regions in which show SES > 0.80 for NYU, PKU1, PKU2 and PKU3, respectively (cluster size > 10).

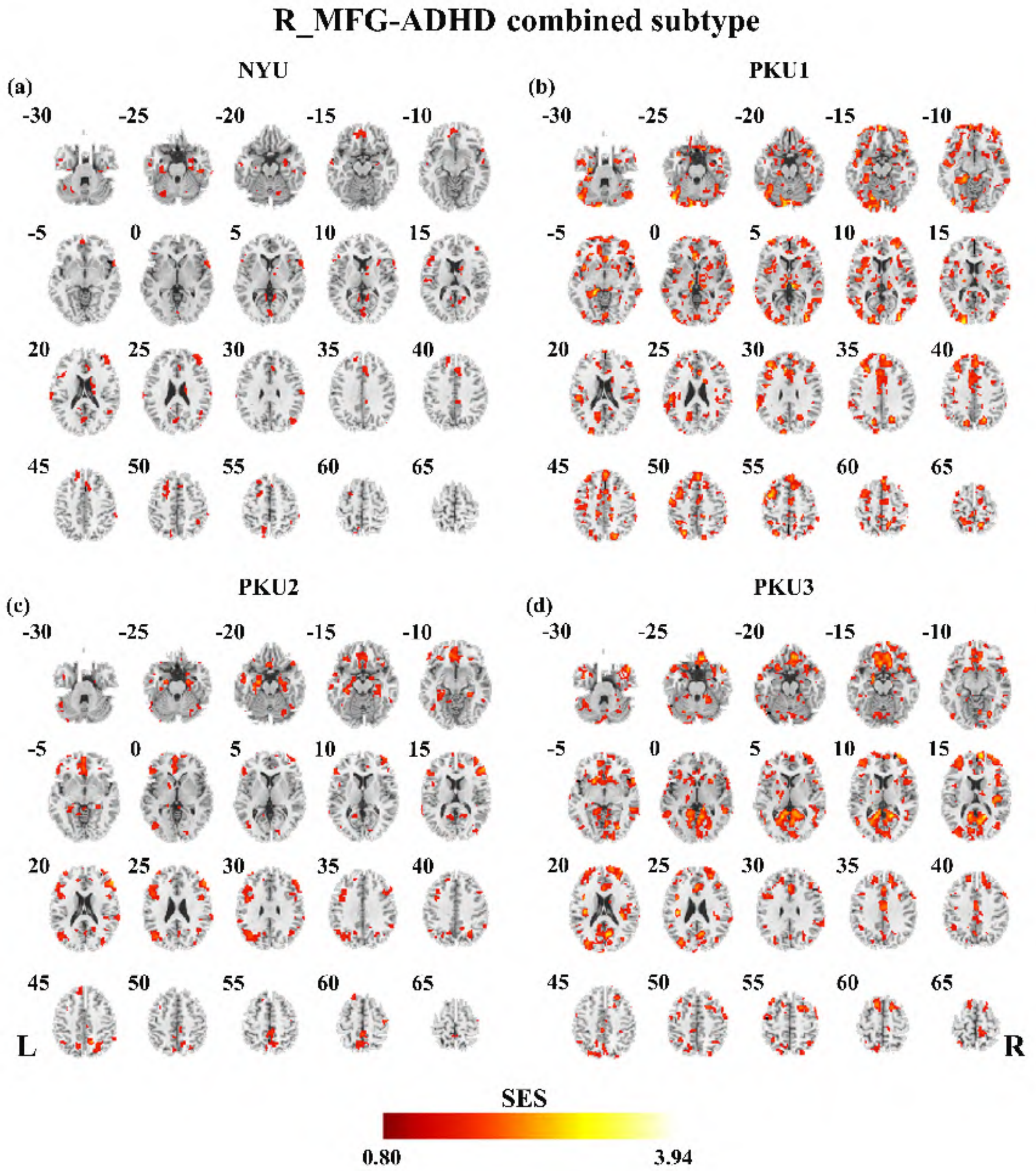

Figure S29. The SES for the difference of seed-based functional connectivity (using R_MFG as the seed region) between children with ADHD combined subtype and TDC. a-d indicate the regions in which show SES > 0.80 for NYU, PKU1, PKU2 and PKU3, respectively (cluster size > 10).

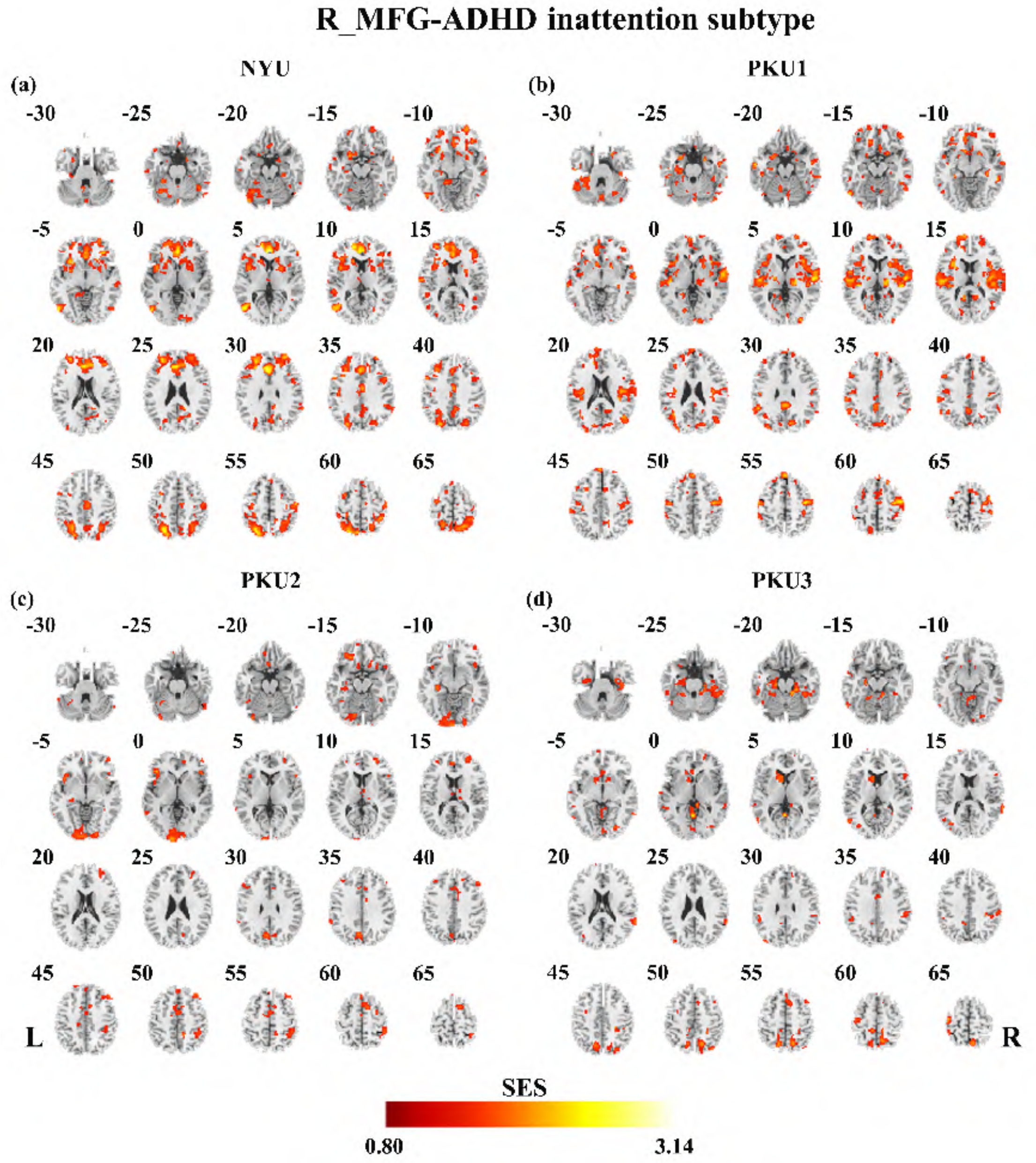

Figure S30. The SES for the difference of seed-based functional connectivity (using R_MFG as the seed region) between children with ADHD inattention subtype and TDC. a-d indicate the regions in which show SES > 0.80 for NYU, PKU1, PKU2 and PKU3, respectively (cluster size > 10).

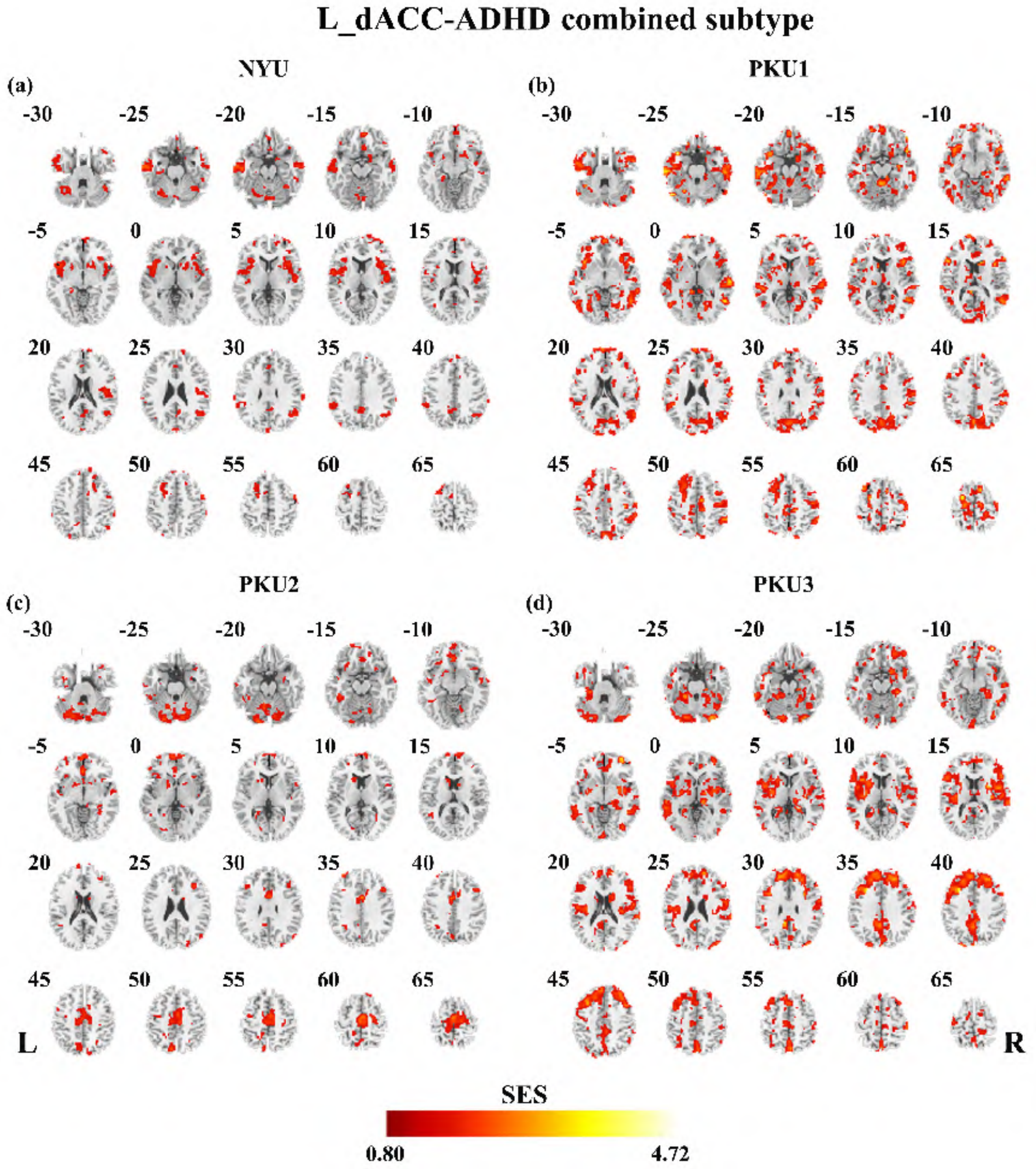

Figure S31. The SES for the difference of seed-based functional connectivity (using L_dACC as the seed region) between children with ADHD combined subtype and TDC. a-d indicate the regions in which show SES > 0.80 for NYU, PKU1, PKU2 and PKU3, respectively (cluster size > 10).

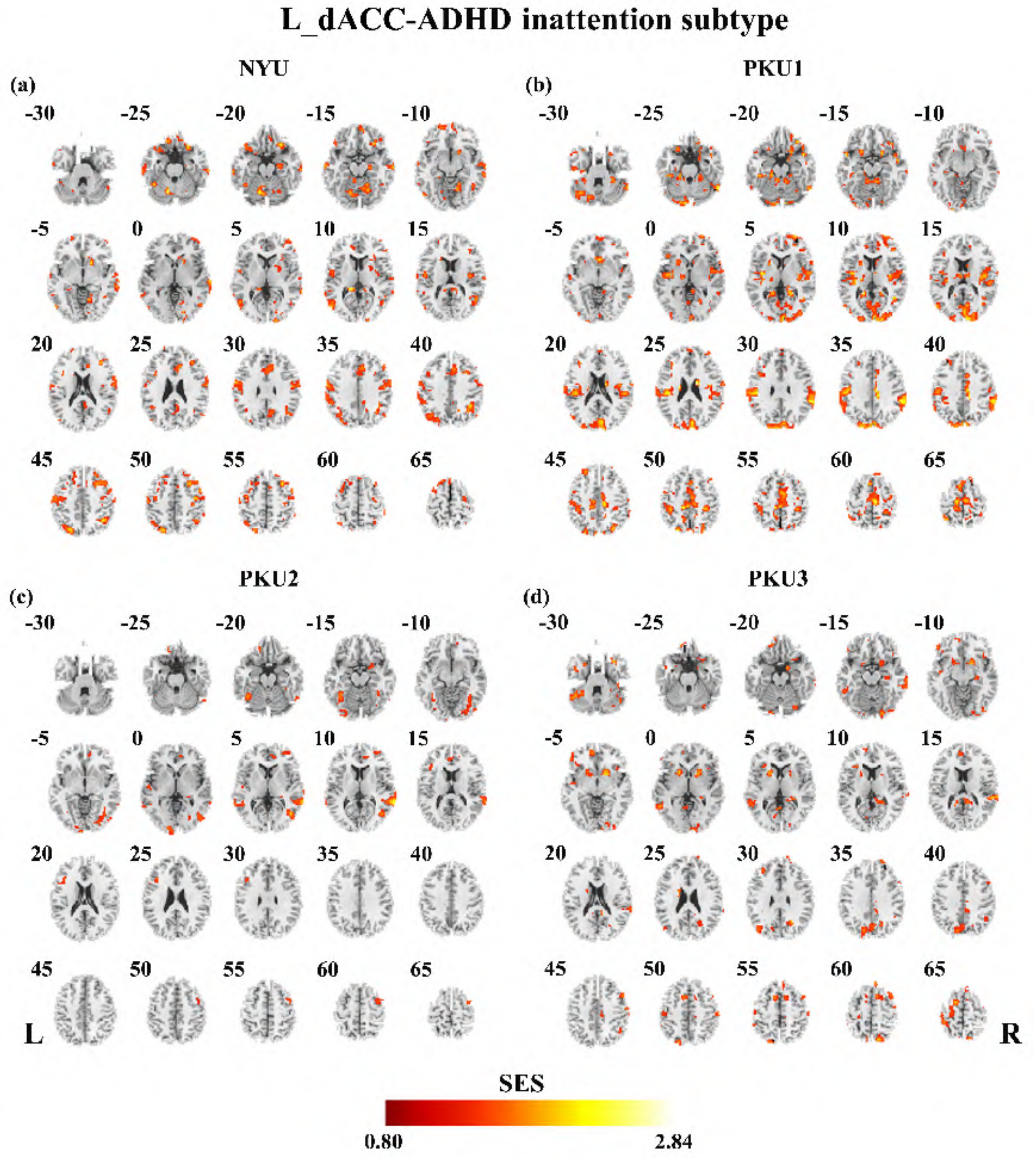

Figure S32. The SES for the difference of seed-based functional connectivity (using L_dACC as the seed region) between children with ADHD inattention subtype and TDC. a-d indicate the regions in which show SES > 0.80 for NYU, PKU1, PKU2 and PKU3, respectively (cluster size > 10).

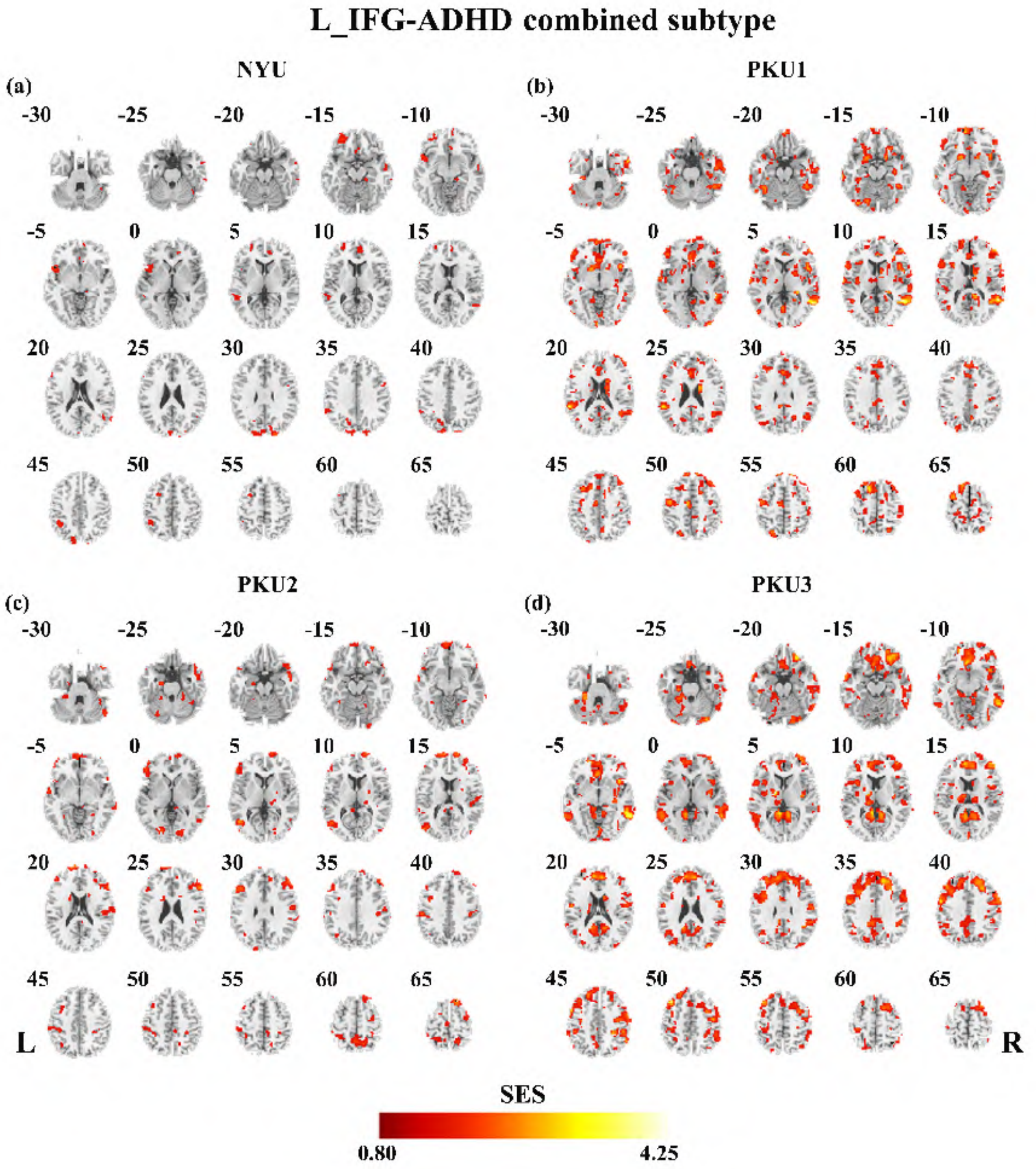

Figure S33. The SES for the difference of seed-based functional connectivity (using L_IFG as the seed region) between children with ADHD combined subtype and TDC. a-d indicate the regions in which show SES > 0.80 for NYU, PKU1, PKU2 and PKU3, respectively (cluster size > 10).

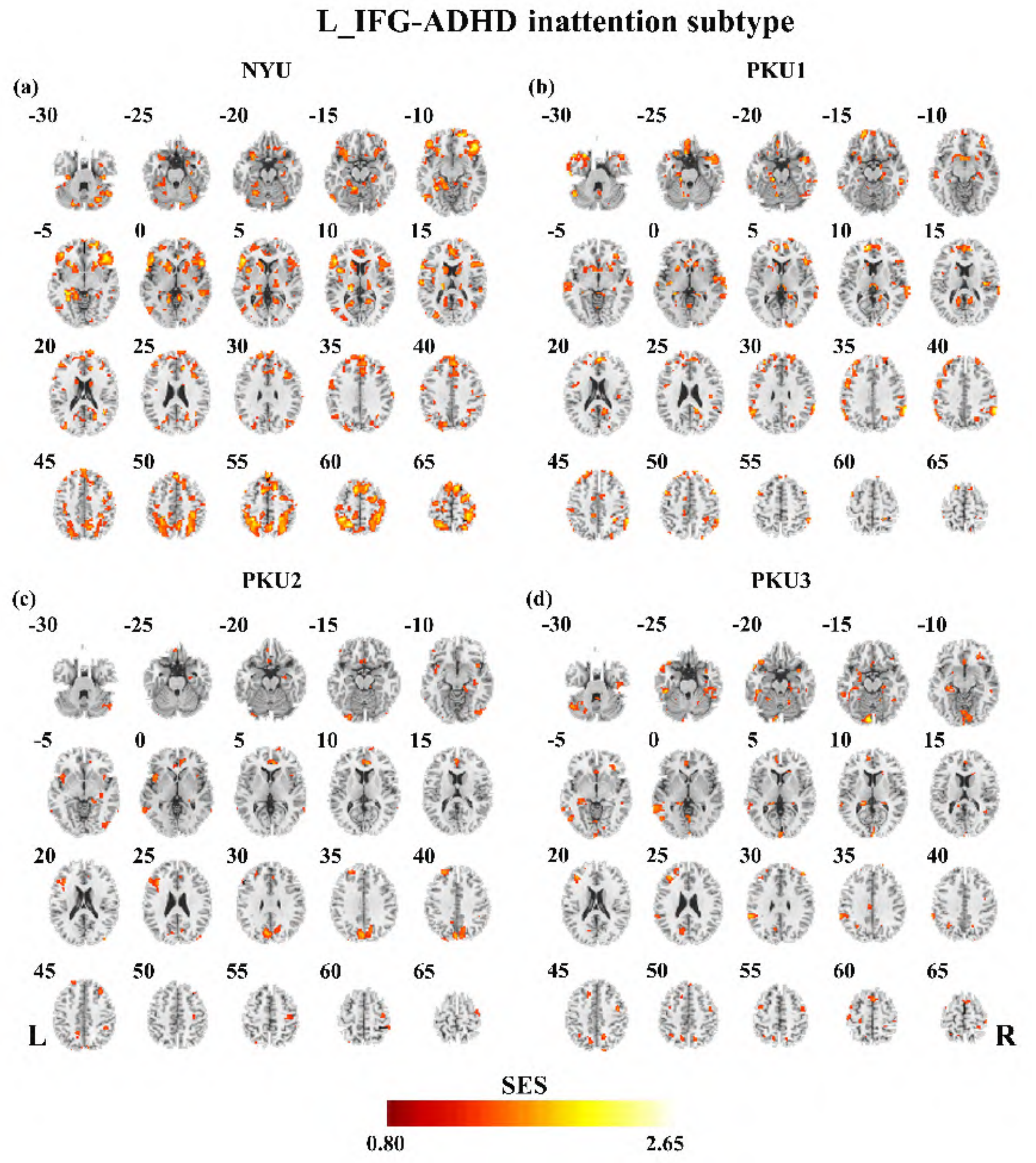

Figure S34. The SES for the difference of seed-based functional connectivity (using L_IFG as the seed region) between children with ADHD inattention subtype and TDC. a-d indicate the regions in which show SES > 0.80 for NYU, PKU1, PKU2 and PKU3, respectively (cluster size > 10).

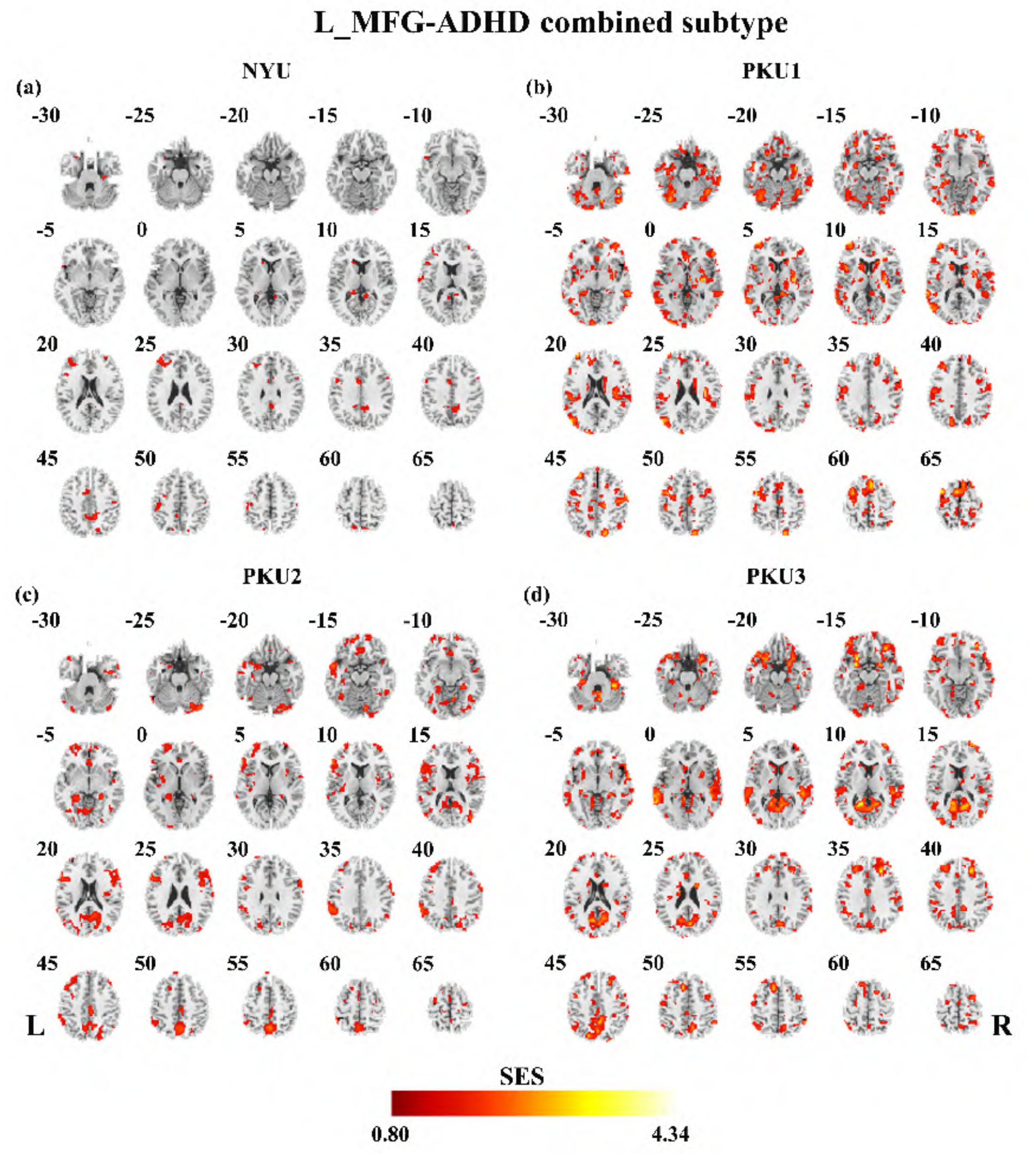

Figure S35. The SES for the difference of seed-based functional connectivity (using L_MFG as the seed region) between children with ADHD combined subtype and TDC. a-d indicate the regions in which show SES > 0.80 for NYU, PKU1, PKU2 and PKU3, respectively (cluster size > 10).

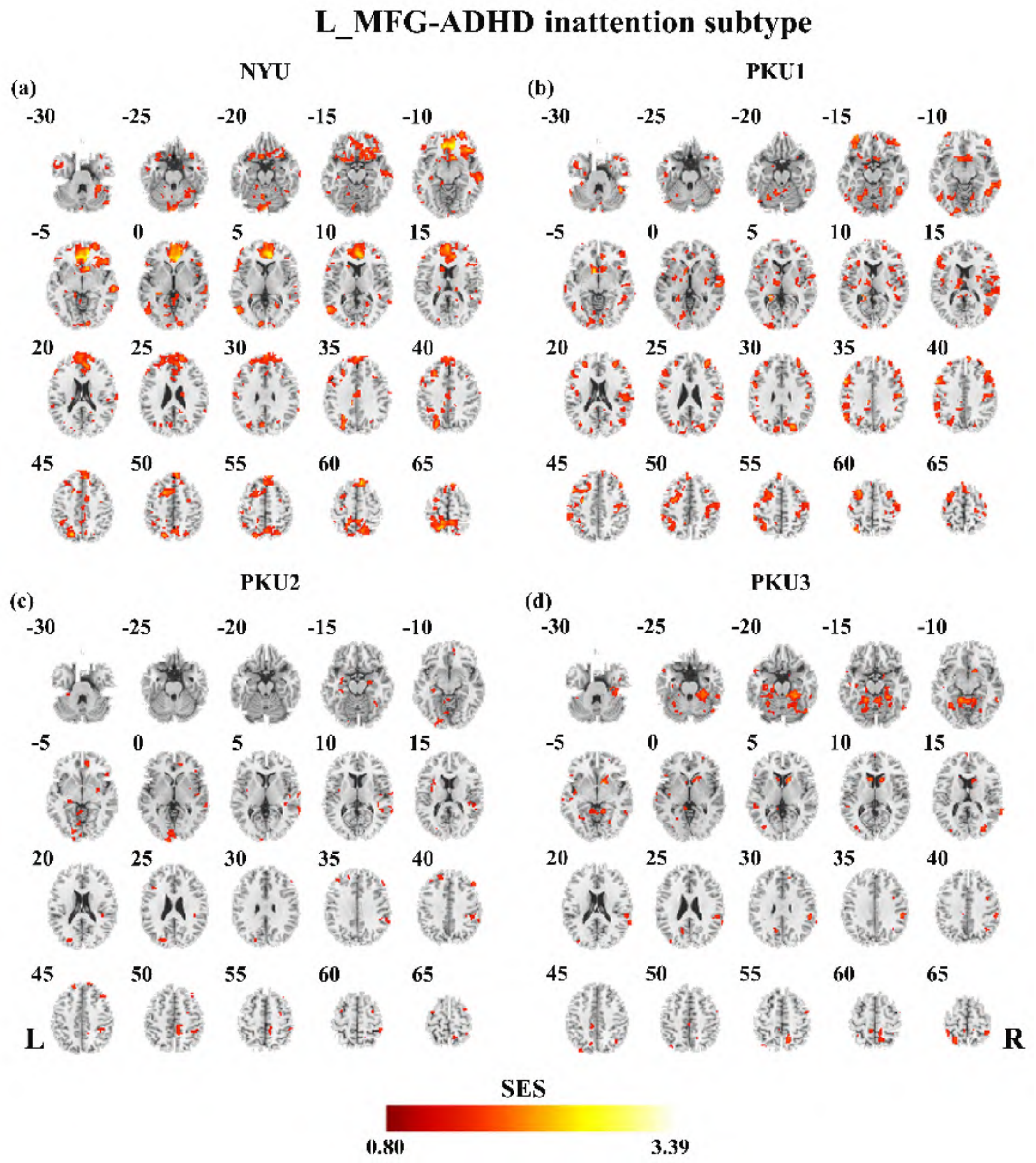

Figure S36. The SES for the difference of seed-based functional connectivity (using L_MFG as the seed region) between children with ADHD inattention subtype and TDC. a-d indicate the regions in which show SES > 0.80 for NYU, PKU1, PKU2 and PKU3, respectively (cluster size > 10).

2.5 Results of negative connectivity between ECN and DMN

To validate these analyses of using public network masks, we also reproduced the analyses based on the functional connectivity of posterior cingulate cortex (PCC). Regions showing positive and negative connectivity with the seed region of PCC was identified as the DMN and ECN respectively (6 mm spherical ROI centered at MNI coordinates: x = 1, y = −55, z = 17 reported by Vincent et al., 2008). Then, for each subject, the mean time courses were extracted based on the masks of DMN and ECN. The negative connectivity between ECN and DMN was calculated through the Pearson correlation between the mean time courses of the two networks. Then, two-sample *t* test was performed for each dataset to test the difference of this negative network connectivity between children with ADHD and TDC.

2.5.1 Negative connectivity between ECN and DMN for ADHD group and TDC group

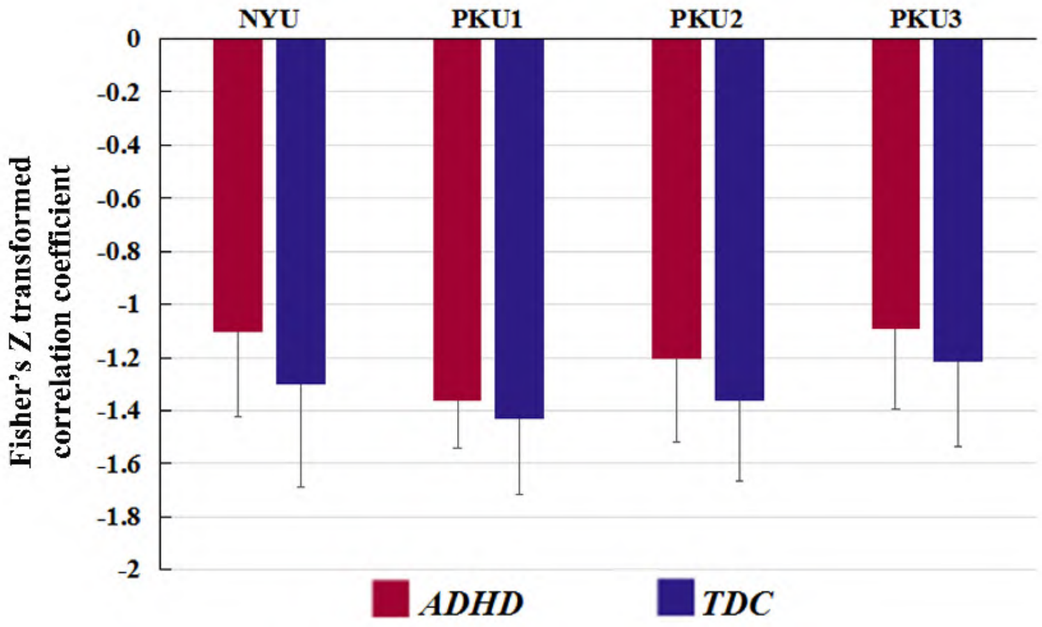

Figure S37. The connectivity between ECN and DMN for children with ADHD and TDC in each dataset.

Table S2. The statistical difference of the negative network connectivity between ADHD group and TDC group. No significant difference could be preserved after the multiple comparison correction.

Dataset	ADHD Group	TDC Group	*t*	*p*
	Mean ± SD	*Mean ± SD*		
**NYU**	-1.10±0.32	-1.30±0.39	2.09	0.04
**PKU1**	-1.37±0.17	-1.43±0.28	0.75	0.46
**PKU2**	-1.20±0.32	-1.37±0.30	1.98	0.05
**PKU3**	-1.09±0.30	-1.22±0.32	1.21	0.23

2.5.2 Connectivity between ECN and DMN for ADHD subtype group and TDC group

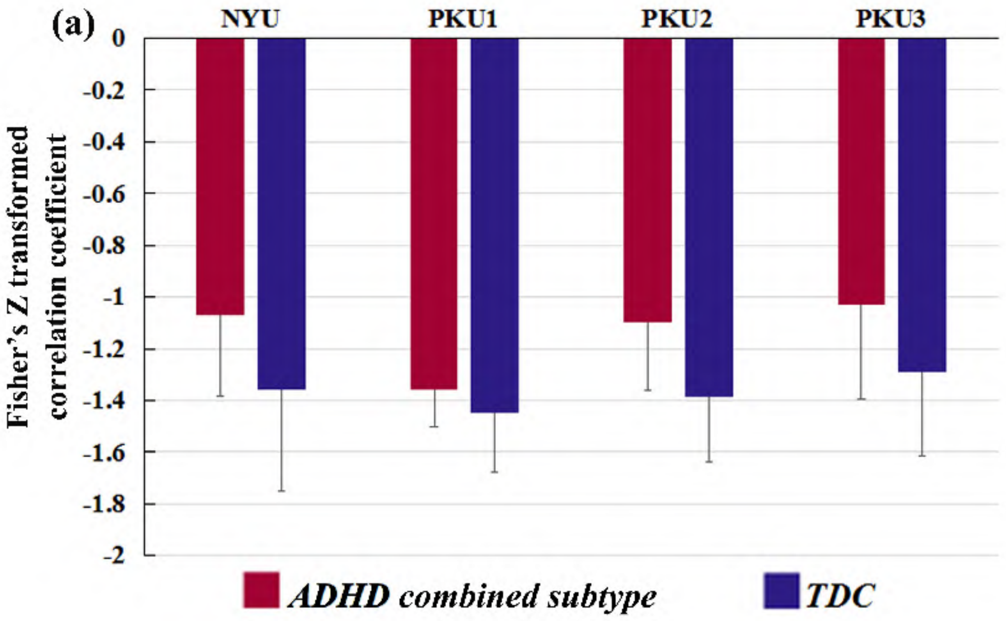

Figure S38. The connectivity between ECN and DMN for children with ADHD combined subtype and TDC in each dataset.

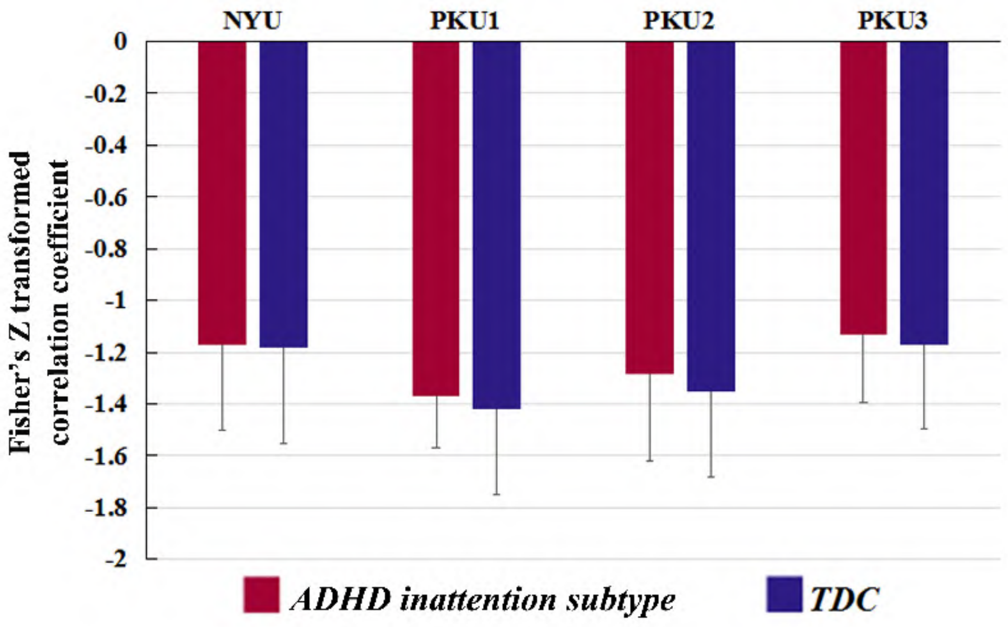

Figure S39. The connectivity between ECN and DMN for children with ADHD inattention subtype and TDC in each dataset.

Table S3. The statistical difference of the negative network connectivity between ADHD subtype group and TDC group.

Dataset	ADHD Group	TDC Group	*t*	*p*
	Mean ± *SD*	*Mean* ± *SD*		
		**ADHD combined subtype**		
**NYU**	-1.07±0.32	-1.36±0.39	2.51	0.02
**PKU1**	-1.36±0.14	-1.45±0.23	0.81	0.44
**PKU2**	-1.10±0.26	-1.39±0.25	2.75	0.01
**PKU3**	-1.03±0.37	-1.29±0.32	1.42	0.18
		**ADHD inattention subtype**		
**NYU**	-1.17±0.33	-1.18±0.37	0.07	0.94
**PKU1**	-1.37±0.20	-1.42±0.33	0.37	0.71
**PKU2**	-1.28±0.34	-1.35±0.33	0.58	0.57
**PKU3**	-1.13±0.26	-1.17±0.32	0.32	0.75

2.6 The similarity and difference of networks across datasets

We examined the similarities and differences between the networks (generated by PCC-based analysis) for all of the 4 datasets. These networks were generated based on the data of each dataset respectively. The percentage of overlapping (PO) was assessed for each dataset, and it was calculated as follows:

$$PO = \frac{Num.voxels(overlapped\ region)}{Num.voxels(whole\ network)} \times 100\%$$

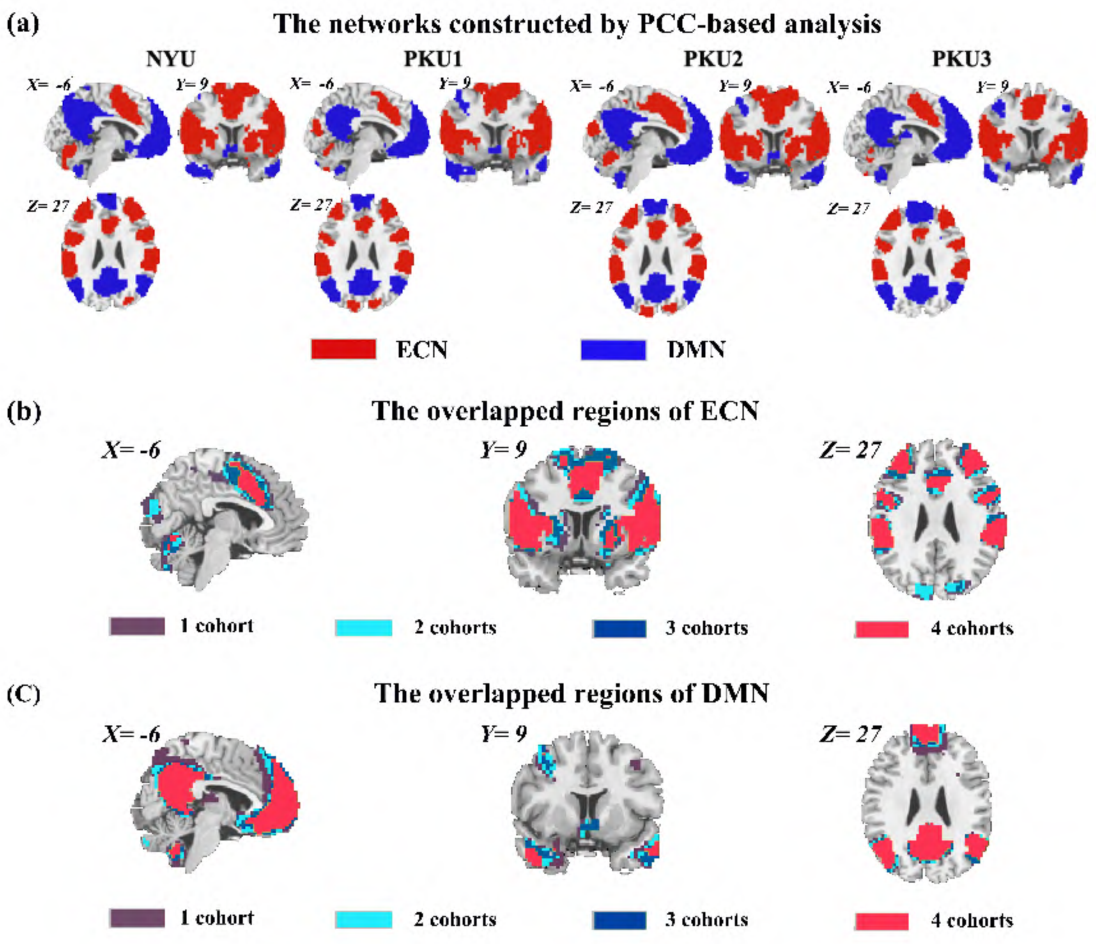

Figure S40. The PCC-based network of each dataset and the overlaps of the 4 datasets.

Table S4. The PO for the networks across all of the 4 datasets.

Dataset	Number of overlapped voxels	PO
**NYU**	23221	58.28%
**PKU1**	20320	66.60%
**PKU2**	26195	51.67%
**PKU3**	20961	64.56%

2.6 The similarity and difference of networks constructed by various analysis methods

We also used another network construction method to minimize the potential influence of the network difference across datasets our findings. The public network masks released by Yeo et al., 2011 were used for all of the 4 datasets. The difference between networks constructed by the two methods was assessed by Dice Coefficient, and it was calculated as follows:

$$Dice\ Coefficient = \frac{2 \times Num.voxels\,(overlapped\ region)}{Num.voxels\,(whole\ two\ network)} \times 100\%$$

Table S5. The Dice Coefficient for the networks constructed by various analysis methods.

Dataset	Number of overlapped voxels	Dice Coefficient
**NYU**	17468	0.59
**PKU1**	16495	0.59
**PKU2**	19476	0.63
**PKU3**	16567	0.58

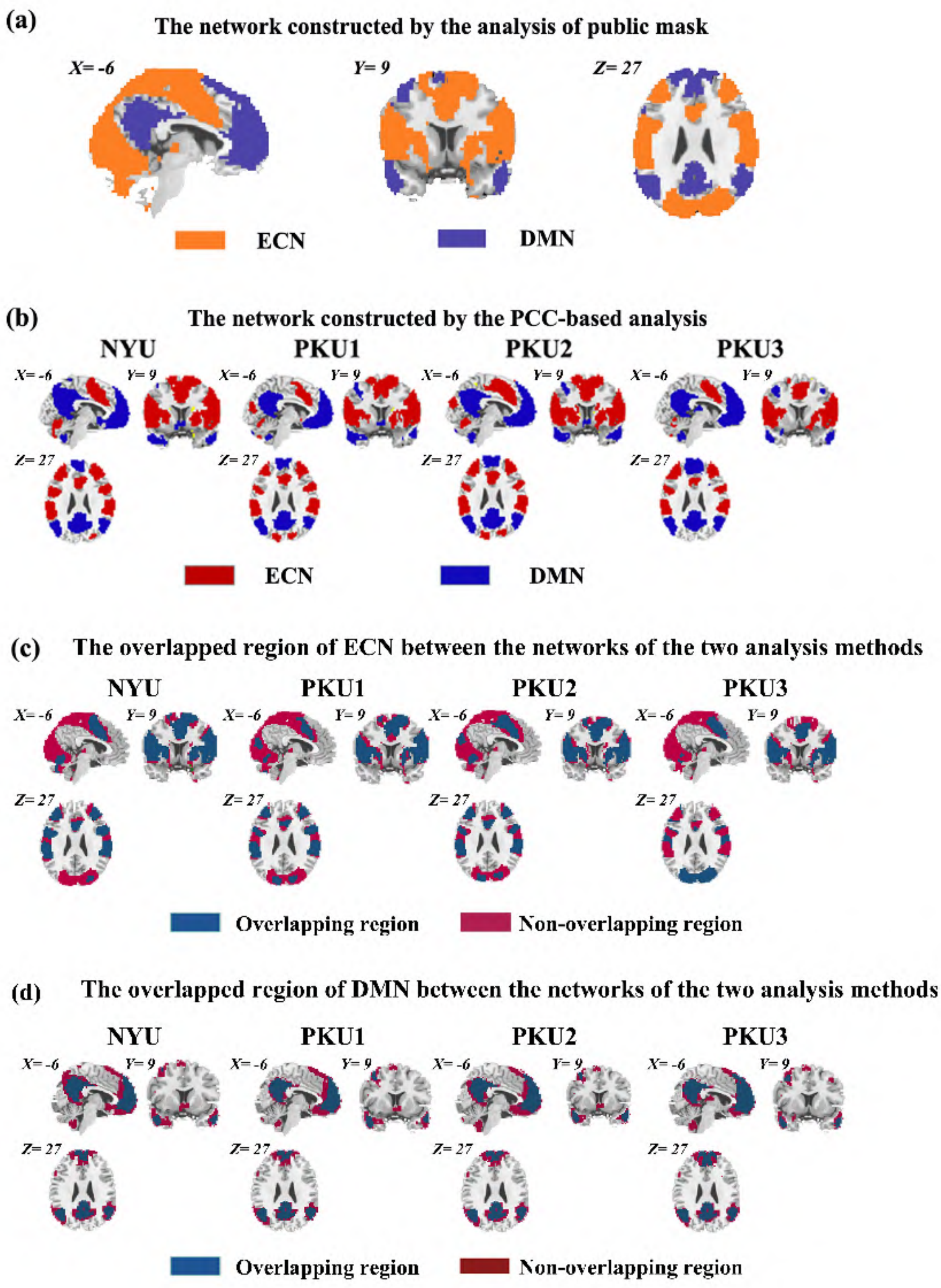

Figure S41. The similarity and difference of networks constructed by various analysis methods.

3. Results for the analysis without removing global mean signal

3.1 Seed-based functional connectivity across datasets

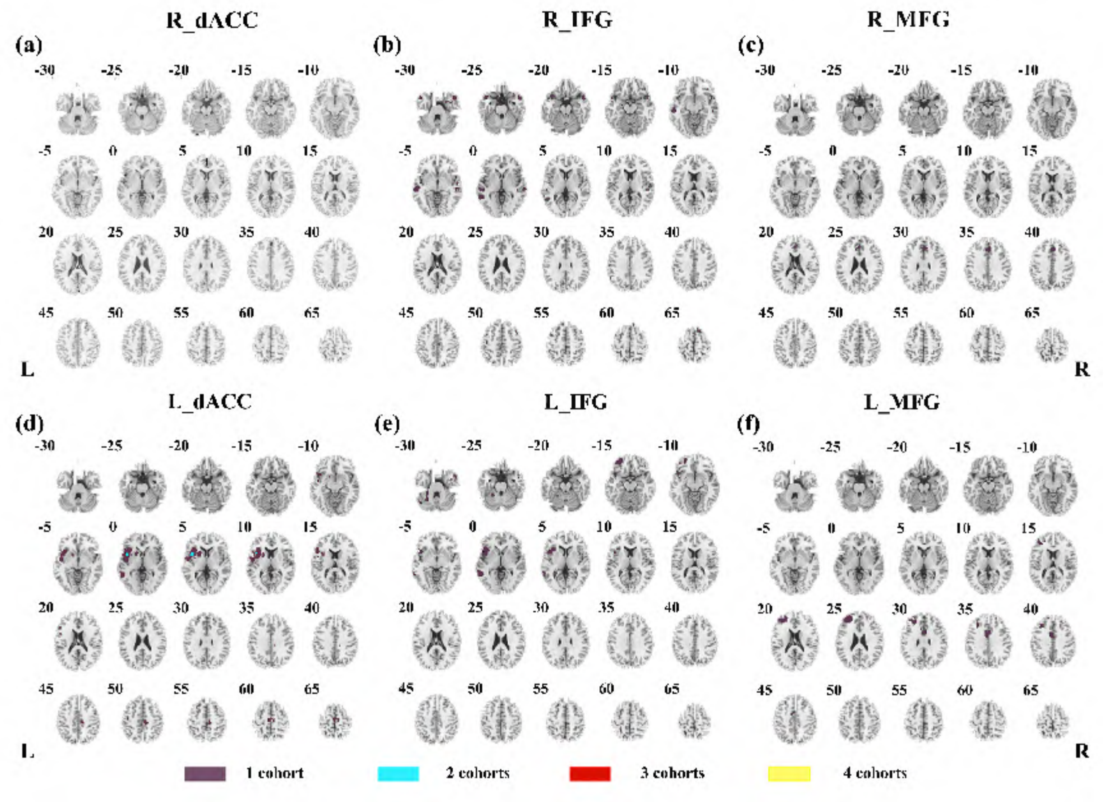

Figure S42. The overlapped results of abnormal functional connectivity for children with ADHD across the 4 datasets. The functional connectivity was assessed with the seed regions of R_dACC, R_IFG, R_MFG, L_dACC, L_IFG and L_MFG respectively. a-f indicate the results with stringent ($p < 0.005$, GRF corrected). Purple indicates the regions detected in only one of the 4 datasets. Mint, red, and yellow indicate the regions detected in 2, 3, and 4 datasets, respectively.

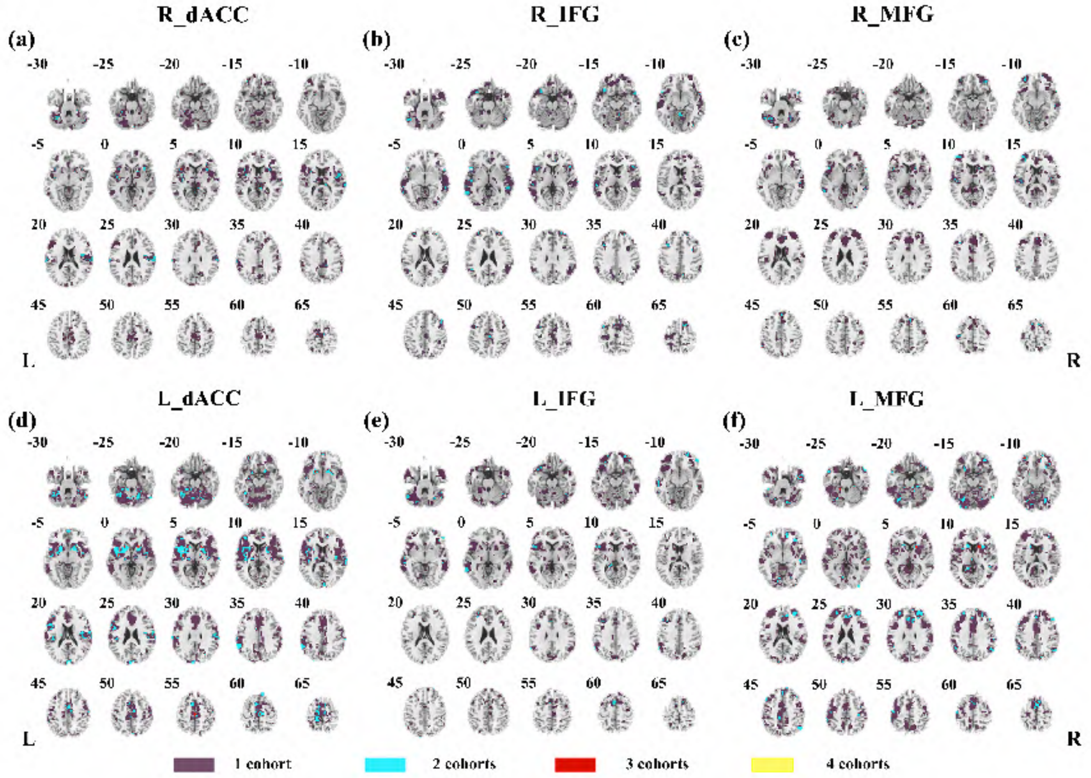

Figure S43. The overlapped results of abnormal functional connectivity for children with ADHD across the 4 datasets. The functional connectivity was assessed with the seed regions of R_dACC, R_IFG, R_MFG, L_dACC, L_IFG and L_MFG respectively. a-f indicate the results with lenient (p < 0.05, cluster size > 10) thresholds. Purple indicates the regions detected in only one of the 4 datasets. Mint, red, and yellow indicate the regions detected in 2, 3, and 4 datasets, respectively.

3.2 The SES for the difference of seed-based functional connectivity between children with ADHD and TDC

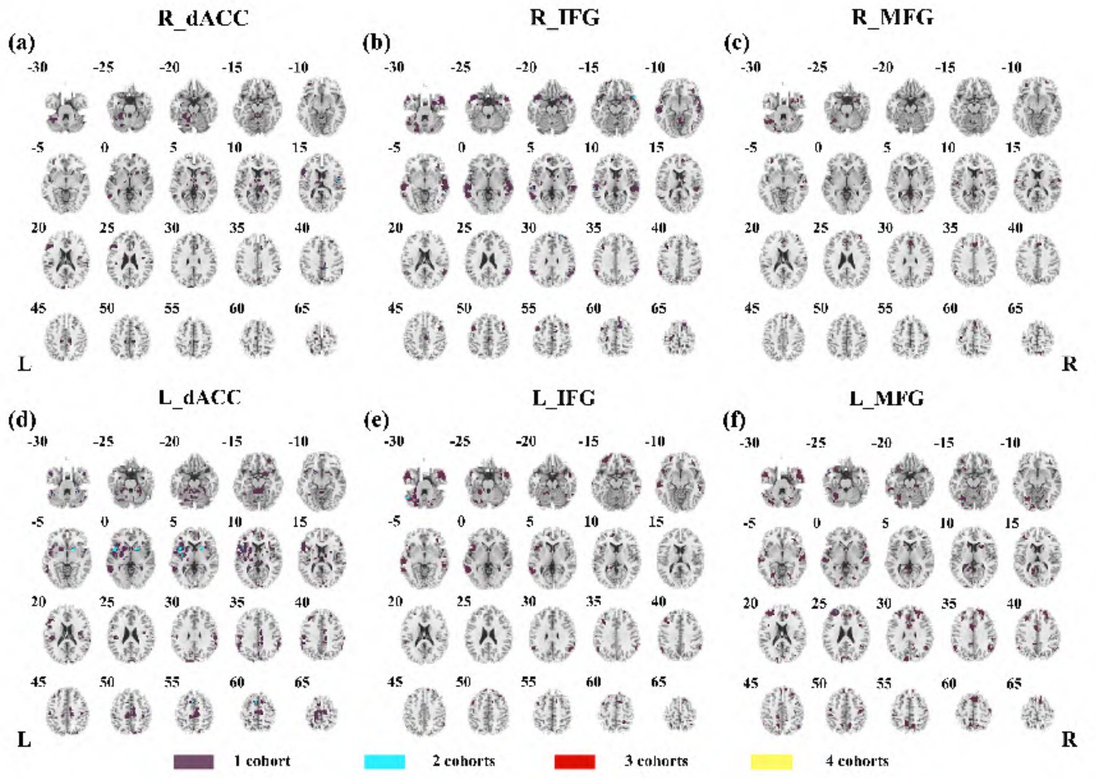

Figure S44. The overlapped SES results across the 4 datasets. The threshold of SES was set at 0.8 for each dataset. a-f indicate the results by using R_dACC, R_IFG, R_MFG, L_dACC, L_IFG and L_MFG as the seed region, respectively. Purple indicate the regions detected only in one of the 4 datasets. Mint indicates the regions detected in 2 datasets. Red indicates the regions detected in only 3 datasets. Yellow indicates the regions detected in 4 datasets.

3.3 The difference of seed-based functional connectivity between children with ADHD subtypes and TDC

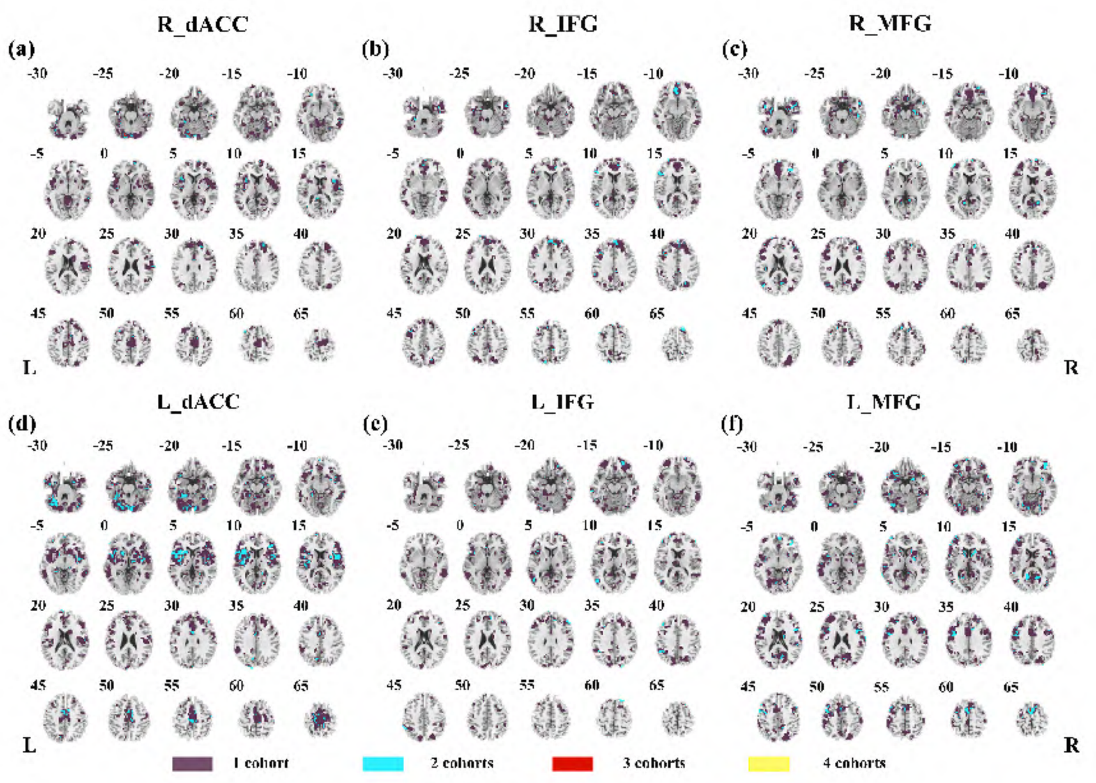

Figure S45. The overlapped results across the 4 datasets in the ADHD combined. Both of them indicate the results detected by using R_dACC, R_IFG, R_MFG, L_dACC, L_IFG and L_MFG as the seed regions, respectively. Purple indicates regions detected in only one of the 4 datasets. Mint, red, and yellow indicate the regions detected in 2, 3, and 4 datasets, respectively.

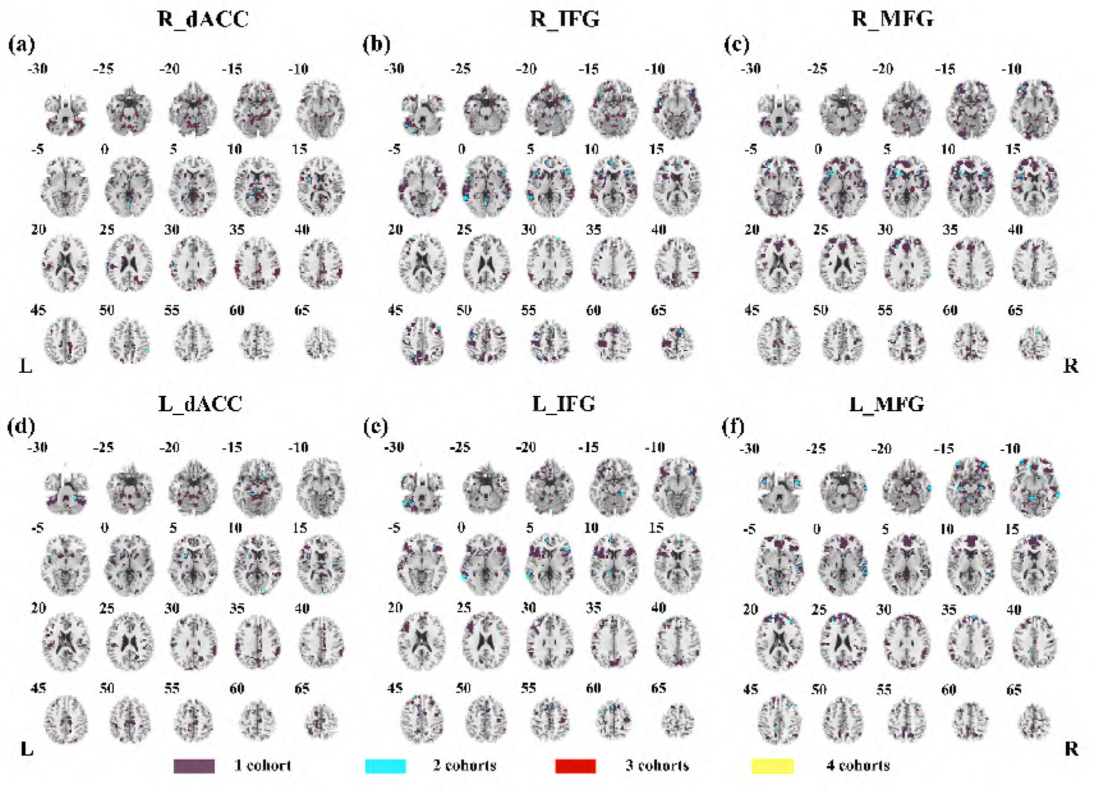

Figure S46. The overlapped results across the 4 datasets in the ADHD inattention subtype. Both of them indicate the results detected by using R_dACC, R_IFG, R_MFG, L_dACC, L_IFG and L_MFG as the seed regions, respectively. Purple indicates regions detected in only one of the 4 datasets. Mint, red, and yellow indicate the regions detected in 2, 3, and 4 datasets, respectively.

3.4 The SES for the difference of the seed-based functional connectivity between children with ADHD subtypes and TDC

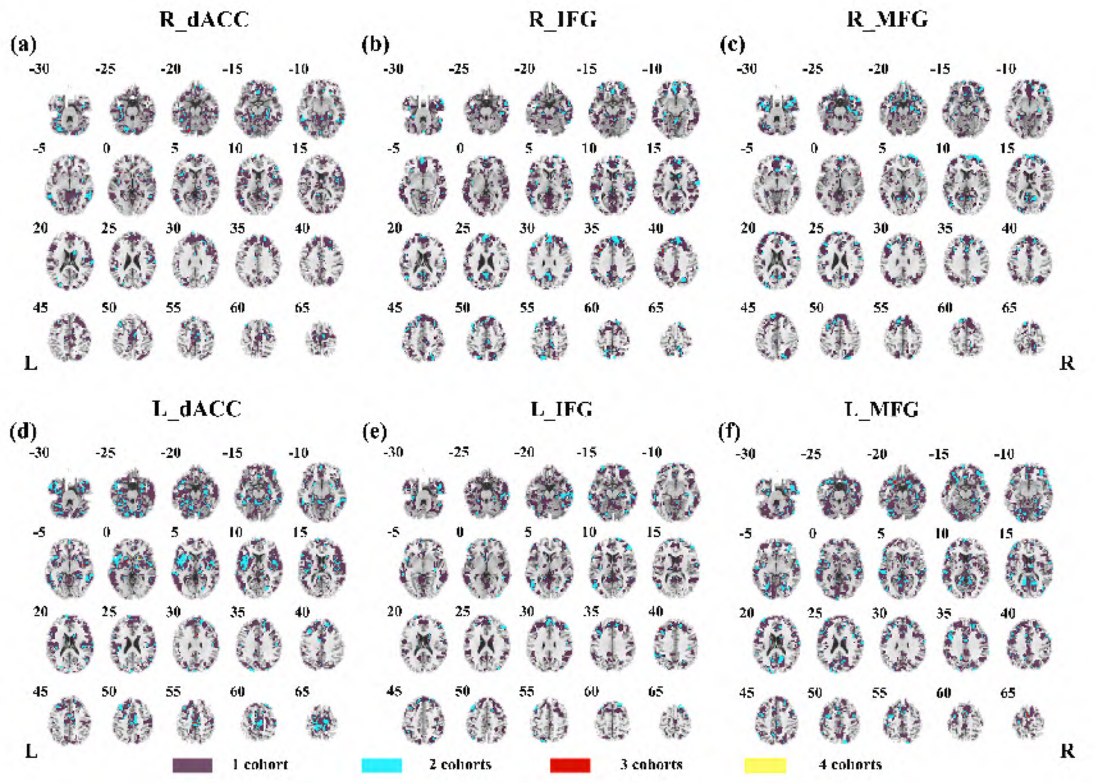

Figure S47. The overlapped SES results of the individual dataset in the ADHD combined subtype. The threshold of SES was set at 0.8 for each dataset. These indicate the results detected by using R_dACC, R_IFG, R_MFG, L_dACC, L_IFG and L_MFG as the seed region, respectively. Purple indicate the regions detected only in one of the 4 datasets. Mint indicates the regions detected in 2 datasets. Red indicates the regions detected by only 3 datasets. Yellow indicates the regions detected in 4 datasets.

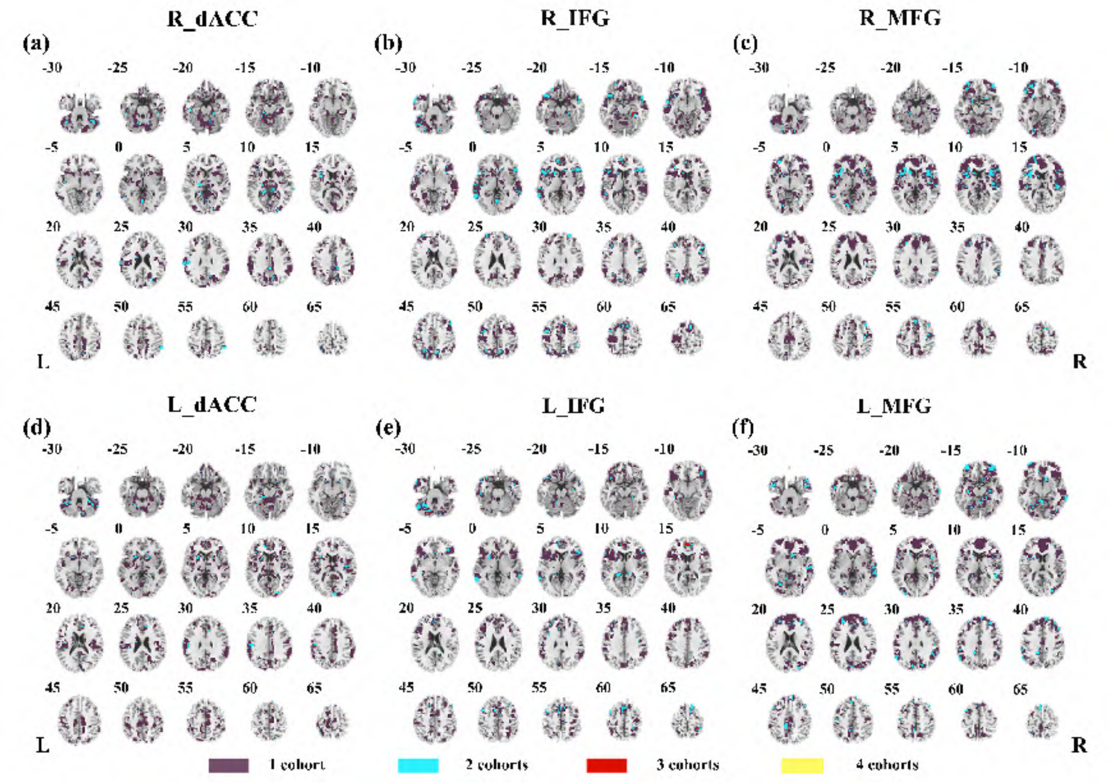

Figure S48. The overlapped SES results of the individual dataset in the ADHD inattention subtype. The threshold of SES was set at 0.8 for each dataset. These indicate the results detected by using R_dACC, R_IFG, R_MFG, L_dACC, L_IFG and L_MFG as the seed region, respectively. Purple indicate the regions detected only in one of the 4 datasets. Mint indicates the regions detected in 2 datasets. Red indicates the regions detected by only 3 datasets. Yellow indicates the regions detected in 4 datasets.

3.5 The Results of connectivity between ECN and DMN

We could not acquire the ECN when the data was not preprocessed by removing global signal effect, because this network was constructed by the calculation of negative connectivity with the seed region of PCC or the public network of DMN, and removing global signal effect is critical for identifying negative functional connectivity. Thus, we directly used the network masks identified in the above analyses of removing global signal effect, and extracted the network time courses based on the data without removing global signal effect. However, we failed to identify the negative connectivity between DMN and ECN. The connectivity between ECN and DMN was positive for both children with ADHD and TDC in each dataset.

3.5.1 The connectivity between ECN and DMN for ADHD group and TDC group by using the public network masks

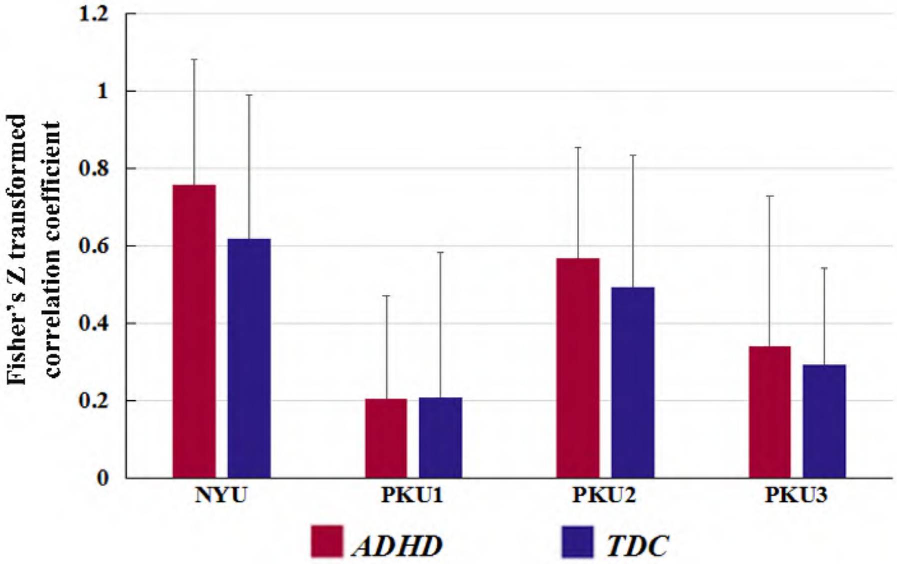

Figure S49. The connectivity between ECN and DMN for children with ADHD and TDC in each dataset.

Table S6. The statistical difference of the network connectivity between ADHD group and TDC group. No significant difference could be preserved after the multiple comparison correction.

Dataset	ADHD Group	TDC Group	*t*	*p*
	Mean ± SD	*Mean ± SD*		
**NYU**	0.76±0.32	0.62±0.37	1.52	0.13
**PKU1**	0.21±0.27	0.21±0.37	-0.02	0.98
**PKU2**	0.57±0.29	0.49±0.34	0.91	0.37
**PKU3**	0.34±0.39	0.29±0.25	0.46	0.65

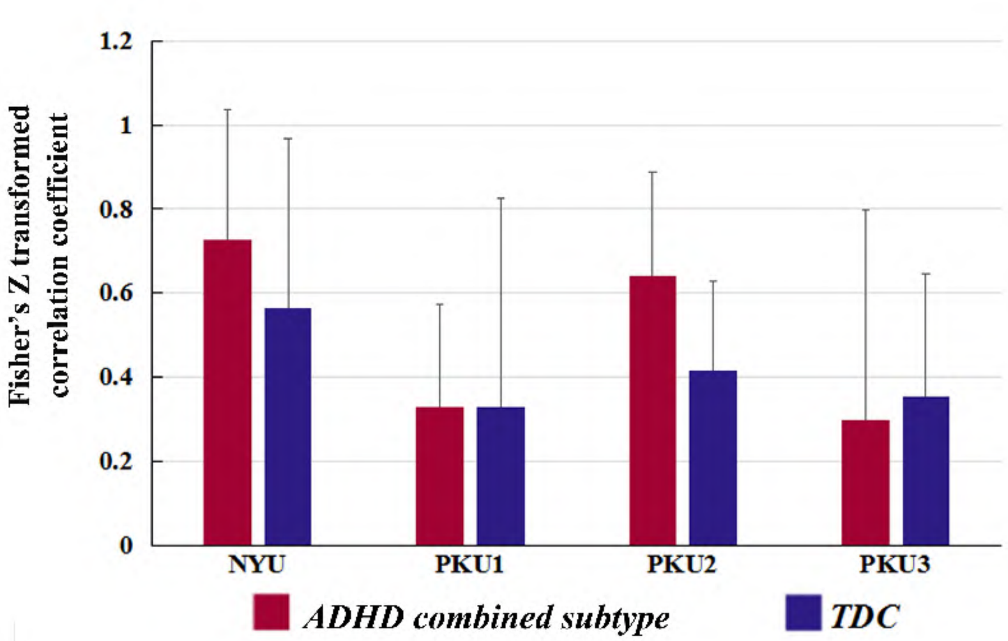

Figure S50. The connectivity between ECN and DMN for children with ADHD combined subtype and TDC in each dataset.

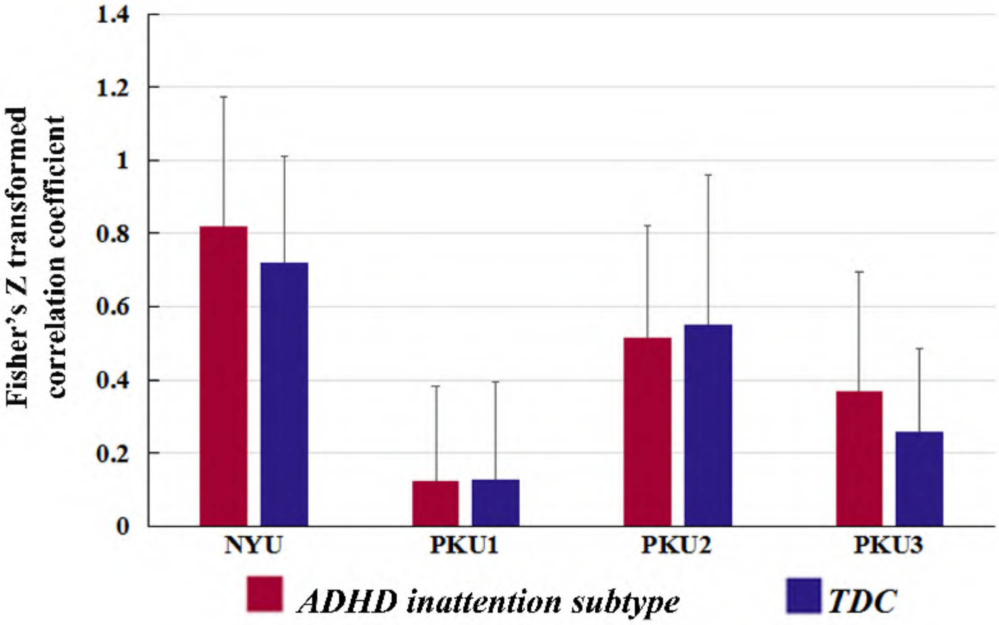

Figure S51. The connectivity between ECN and DMN for children with ADHD inattention subtype and TDC in each dataset.

Table S7. The statistical difference of the network connectivity between ADHD subtype group and TDC group. No significant difference could be preserved after the multiple comparison correction.

**Dataset**	**ADHD Group**	**TDC Group**	***t***	***p***
	Mean ± SD	***Mean ± SD***		
		**ADHD combined subtype**		
**NYU**	0.73±0.31	0.56±0.40	1.37	0.18
**PKU1**	0.33±0.25	0.33±0.50	-0.002	1.00
**PKU2**	0.64±0.25	0.41±0.21	2.40	0.03
**PKU3**	0.30±0.50	0.35±0.29	-0.26	0.80
		**ADHD inattention subtype**		
**NYU**	0.82±0.35	0.72±0.29	0.67	0.51
**PKU1**	0.12±0.26	0.13±0.27	-0.03	0.97
**PKU2**	0.51±0.31	0.55±0.41	-0.28	0.78
**PKU3**	0.37±0.33	0.26±0.23	0.95	0.35

3.5.2 The connectivity between ECN and DMN for ADHD group and TDC group with the network masks constructed by PCC analysis

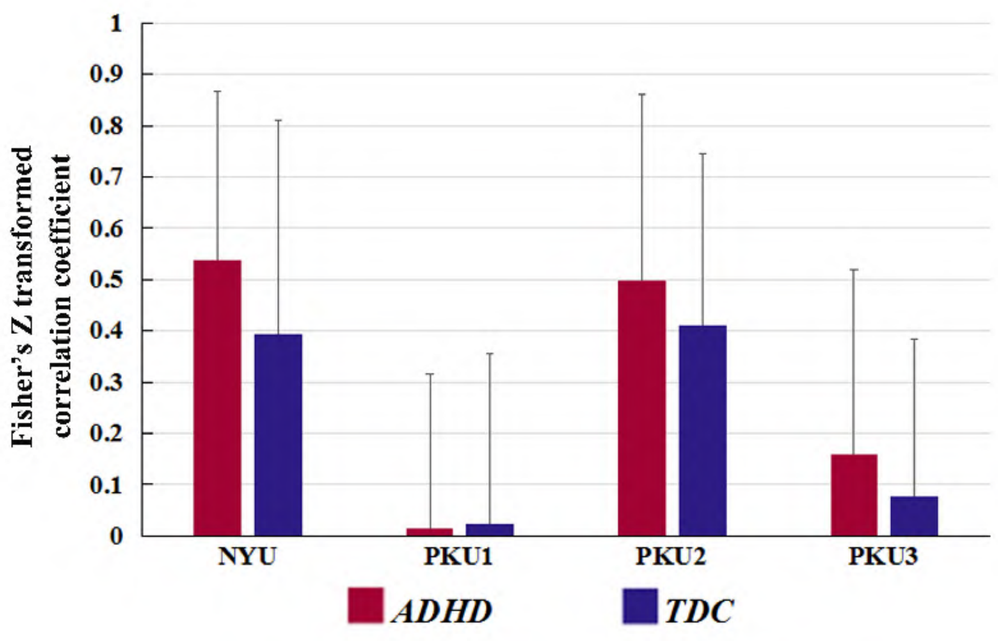

Figure S52. The connectivity between ECN and DMN for children with ADHD and TDC in each dataset.

Table S8. The statistical difference of the network connectivity between ADHD group and TDC group. No significant difference could be preserved after the multiple comparison correction.

Dataset	ADHD Group	TDC Group	*t*	*p*
	Mean ± SD	***Mean ± SD***		
**NYU**	0.54 ± 0.33	0.39 ± 0.42	1.46	0.15
**PKU1**	0.01±0.30	0.02 ± 0.33	-0.09	0.93
**PKU2**	0.50 ± 0.36	0.41 ± 0.33	0.95	0.34
**PKU3**	0.16 ± 0.36	0.08 ± 0.31	0.75	0.46

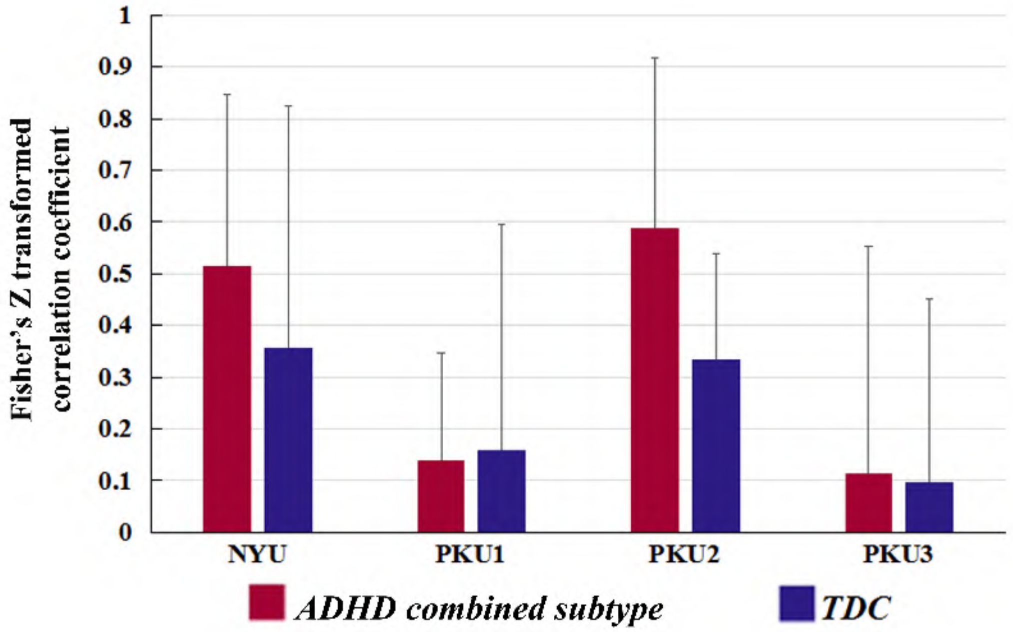

Figure S53. The connectivity between ECN and DMN for children with ADHD combined subtype and TDC in each dataset.

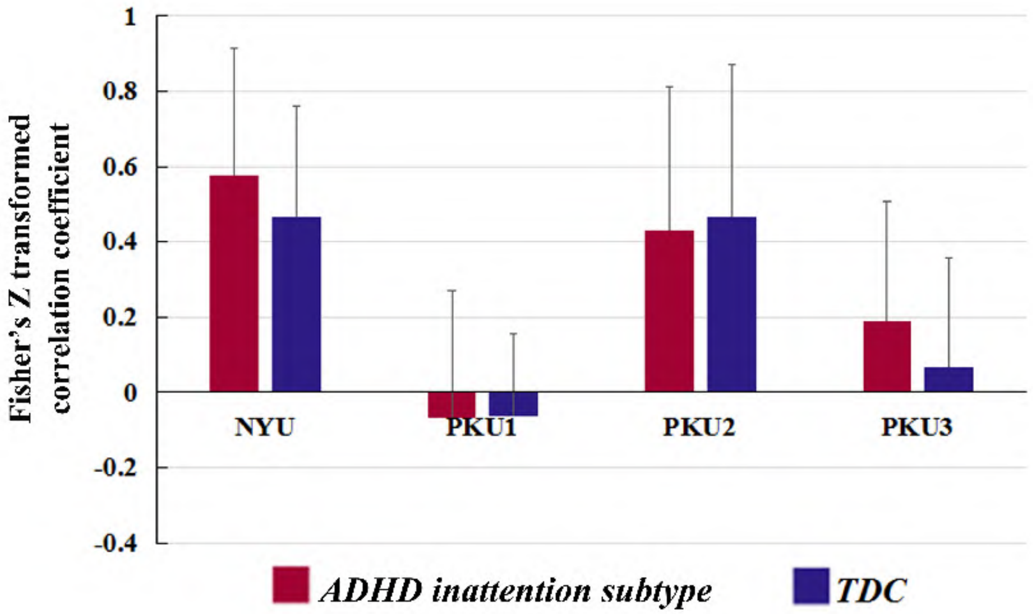

Figure S54. The connectivity between ECN and DMN for children with ADHD inattention subtype and TDC in each dataset.

Table S9. The statistical difference of the network connectivity between ADHD subtype groups and TDC group. No significant difference could be preserved after the multiple comparison correction.

**Dataset**	**ADHD Group**	**TDC Group**	***t***	***p***
	Mean* ± *SD	***Mean* ± *SD***		
**ADHD combined subtype**				
**NYU**	0.51±0.33	0.36±0.47	1.21	0.24
**PKU1**	0.14±0.21	0.16±0.44	-0.10	0.92
**PKU2**	0.59±0.33	0.33±0.20	2.28	0.03
**PKU3**	0.11±0.44	0.10±0.36	0.08	0.94
**ADHD inattention subtype**				
**NYU**	0.58±0.34	0.46±0.30	0.80	0.44
**PKU1**	-0.07±0.34	-0.07±0.22	-0.02	0.98
**PKU2**	0.43±0.38	0.47±0.40	-0.26	0.80
**PKU3**	0.19±0.32	0.07±0.29	0.96	0.35
